# 5′-Chalcogen-Substituted Nucleoside Pyrophosphate and Phosphate Monoester Analogues: Preparation and Hydrolysis Studies

**DOI:** 10.3390/ijms232415582

**Published:** 2022-12-08

**Authors:** Satu Mikkola, Olga Eguaogie, Anu Nieminen, Patrick F. Conlon, David L. Jakeman, Keith Moore, Ian C. Lane, Joseph S. Vyle

**Affiliations:** 1 Department of Chemistry, University of Turku, FIN-20014 Turku, Finland; 2 School of Chemistry and Chemical Engineering, Queen’s University of Belfast, David Keir Building, Stranmillis Road, Belfast BT9 5AG, UK; 3 College of Pharmacy, Dalhousie University, Halifax, NS B3H 4R2, Canada

**Keywords:** hydrolysis, kinetics, nucleotide analogues, pyrophosphate, capillary zone electrophoresis, mechanochemistry, Michaelis-Arbuzov, ^31^P NMR

## Abstract

Novel sulfur and selenium substituted 5′,5′-linked dinucleoside pyrophate analogues were prepared in a vibration ball mill from the corresponding persilylated monophosphate. The chemical hydrolysis of pyrophosphorochalcogenolate-linked dimers was studied over a wide pH-range. The effect of the chalcogeno-substitution on the reactivity of dinucleoside pyrophosphates was surprisingly modest, and the chemical stability is promising considering the potential therapeutic or diagnostic applications. The chemical stability of the precursor phosphorochalcogenolate monoesters was also investigated. Hydrolytic desilylation of these materials was effected in aqueous buffer at pH 3, 7 or 11 and resulted in phosphorus-chalcogen bond scission which was monitored using ^31^P NMR. The rate of dephosphorylation was dependent upon both the nature of the chalcogen and the pH. The integrity of the P-S bond in the corresponding phosphorothiolate was maintained at high pH but rapidly degraded at pH 3. In contrast, P-Se bond cleavage of the phosphoroselenolate monoester was rapid and the rate increased with alkalinity. The results obtained in kinetic experiments provide insight on the reactivity of the novel pyrophosphates studied as well as of other types of thiosubstituted biological phosphates. At the same time, these results also provide evidence for possible formation of unexpectedly reactive intermediates as the chalcogen-substituted analogues are metabolised.

## 1. Introduction

Stimuli-responsive drug delivery systems are well established in the treatment of cancer following the observation that selective uptake of haematoporphyrin (via LDL-derived nanoparticles) into malignant tissue subsequently enabled effective photodynamic therapy in the early 20th century [1]. Side effects associated with the cytotoxicity of such drugs can be ameliorated by covalently linking the API to a tumour-selective targeting moiety such as an antibody [2], aptamer [3], oligopeptides [4] or small molecules [5,6,7]. 

**Figure 1:**
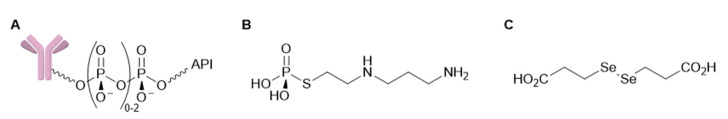
Anticancer agents including ionizable functions which improve solubility: (**A**) antibody-drug conjugates; (**B**). amifostine; (**C**) 3,3′-diselenodipropionic acid.

The nature of the linkages will often be engineered both to alter the solubility of the conjugate and to enable highly localised release of the payload upon interaction with the special pathophysiological conditions of the tumour microenvironment [8]. Recently, enzyme-sensitive conjugates have been described in which phosphate, pyrophosphate or triphosphate moieties (Figure 1A) have been employed as linkers between tumour-specific antibodies and glucocorticoid receptor agonists [9,10] or a proteolysis targeting chimera [11] which enhance their aqueous solubility and liberate their cargo following the action of intracellular, endosomal pyrophosphatases. In contrast, differences between extracellular enzymatic phosphate monoesterase activities in neoplastic and healthy tissue has also been exploited in the application of the well-established adjuvant amifostine as a radioprotectant [12]. More recently, organoselenium compounds such as 3,3′-diselenodipropionic acid (DSePA) have also been reported to act as radioprotectants [13]. However, both the phosphorothiolate monoester group in amifostine and the diselenide function in DSePA can be sensitive towards chemical modification outside of that desired for their therapeutic activities.

Recently, we [14,15] and others [16] have described the synthesis of pyrophosphorothiolate-linked dinucleotide cap analogues which are considerably more resilient towards chemical hydrolysis than the corresponding monoesters, especially at acidic or neutral pH [14,17,18]. Such linkages (and the related pyrophosphoroselenolates) thus have the potential to provide a method for delivering therapeutic thiol and selenol functions which could be metabolically unmasked following pyrophosphatase cleavage.

Understanding the scope for biochemical unmasking of nucleoside chalcogen pharmacophores either following endocytosis or via the action of extracellular enzymes such as tissue non-specific alkaline phosphatase (TNAP) or ecto-nucleotide pyrophosphatase /phosphodiesterases (ENPP’s) [19] is limited by the lack of data relating to the chemical hydrolysis rates of sulfur- or selenium-substituted pyrophosphates or phosphate monoesters at different pH values. Despite their ubiquity during enzymatic selenisation of biomolecules [20,21], phosphoroselenolate monoesters are challenging targets for chemical synthesis with very limited precedent [22,23]. In contrast, the corresponding silyl ethers are known to be relatively stable [23,24]. Likewise, we demonstrated that persilylated phosphorothiolate monoester derivatives of nucleosides were found to be stable for a week at ambient temperature under anhydrous conditions and could be efficiently accessed via Michael-Arbuzov chemistry [14]. Such chemistry therefore provides access to relatively stable and pure materials which can readily be hydrolysed to the labile corresponding chalcogenate monoesters under controlled conditions and in the current report we have exploited this reactivity to examine the labilities of 5′-thionucleoside and 5′-deoxy-5′-selenonucleoside monophosphate analogues under different pH conditions. Furthermore, in the presence of sub-stoichiometric quantities of water, these intermediates undergo phosphate coupling in a ball mill and have thereby enabled the preparation of unprecedented pyrophosphorothiolate or pyrophosphoroselonolate-linked dinucleosides. We show that these have considerably higher chemical resilience towards acid pH than the corresponding monoesters and may thereby provide a mechanism for delivering water-soluble masked chalcogenonucleosides. 

**Figure 2:**
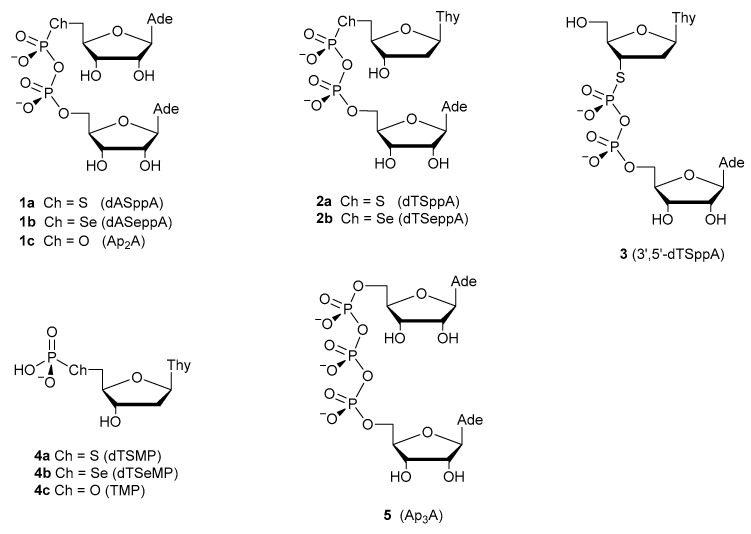
Structures of chalcogen-substituted nucleotide analogues and the natural pyrophosphate-linked dimers, Ap_2_A and Ap_3_A. Key. Ade: *N*^9^-adeninyl. Thy: *N*^1^-thyminyl.

The reactivity of nucleoside 5′-monophosphorochalcogenolates and pyrophosphorochalcogenolate-linked dinucleosides is interesting also from a mechanistic point of view. It is well known that while phosphorothioate-linked RNA model compounds are chemically approximately as reactive as corresponding phosphate linked compounds [25,26], a phosphorothiolate linkage is significantly more reactive [27,28,29,30,31]. Substitution of bridging oxygen atoms in RNA model dimers results in up to a 10^5^-fold rate enhancement following substitution of the leaving group 5′-oxygen [27,28]. The effect of substituting a 3′-oxygen is more modest, but not insignificant [29,30,31]. The magnitude of the effects depends on the conditions and the properties of the nucleophile and leaving group. Studies on the reactivity of pyrophosphates such as **1a**–**b**, **2a**–**b** and **3** (Figure 2), their decomposition products and monophosphates **4a**–**b** offer the possibility to further evaluate the factors that influence the reactivity of different types of chalcogen-substituted nucleotide analogues. 

## 2. Results

### 2.1. Synthesis of Pyrophosphorochalcogenolate-Linked Dinucleosides

**Scheme 1:**
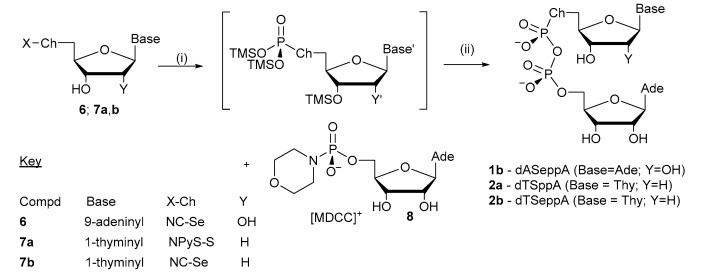
Synthesis of 5′,5′-pyrophosphorochalcogenolate-linked dinucleosides dASeppA (**1b**), dTSppA (**2a**) and dTSeppA (**2b**). Reagents and conditions. (**i**) (TMSO)_3_P, *N*,*O*-bis(trimethylsilyl)acetamide, CDCl_3_; (**ii**) (a) **8**, tetrazole, MgCl_2_.6H_2_O, H_2_O, LAG, 30 Hz. 90 min, (b) H_2_O, CH_3_OH. Key. Base’: Ade; Y′: OTMS (from **6**) or Base’: Thy*^O4^*^TMS^; Y′: H (from **7a**, **7b**). MDCC = *N*,*N*′-dicyclohexylcarboxamidinium; NPyS = 5-nitropyridine-2-sulfenyl.

Previously, we described the application of liquid assisted grinding (LAG) in a vibration ball-mill for the construction of 5′,5′- (**1a**) and 3′,5′- (**3**) pyrophosphorothiolate linked dinucleosides from the corresponding persilylated 5′- or 3′-thionucleoside phosphorothiolate monoesters, respectively [14]. This procedure was adapted to the synthesis of the novel 5′,5′-pyrophosphoroselenolate linked dimers dASeppA (**1b**) and dTSeppA (**2b**) as well the corresponding sulfur-substituted analogue dTSppA (**2a**)—Scheme 1. 

**Figure 3:**
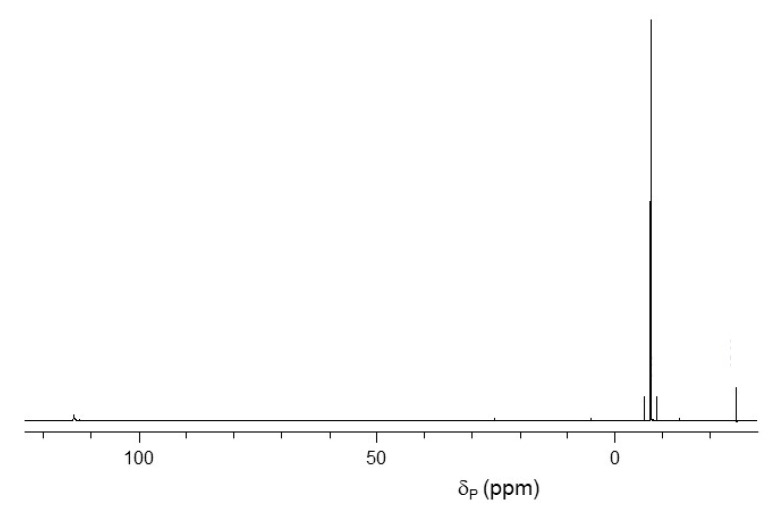
^31^P{^1^H} NMR spectra of crude reaction mixture of 1.1 equiv. (TMSO)_3_P (δ_P_ 113.5 ppm) with 5′-deoxythymidine-5′-selenocyanate (**7b**) in 4:1 CHCl_3_:BSA after 18 h at room temperature (for conditions, see Supplementary Materials).

In a typical reaction, a solution of 5′-deoxythymidine-5′-selenocyanate (**7b**) [32] in 4:1 chloroform:*N*,*O*-bis(trimethylsilyl)acetamide (BSA) was treated with a solution of tris(trimethylsilyl)phosphite (1.1 equiv) in the same solvent. This Michaelis-Arbuzov (MA) reaction mixture was stored overnight at room temperature following which ^31^P NMR analysis showed complete reaction to essentially a single peak at δ −7.8 ppm accompanied by satellite peaks associated with ^77^Se coupling (^1^*J*_PSe_ = 439 Hz) (Figure 3). These values are consistent with those reported by Borecka et al., for (TMSO)_2_P(O)SeMe (δ_P_ −7 ppm; ^1^*J*_PSe_ = 472 Hz) [23] and follows the trend observed with silylation of phosphorothiolate esters which results in upfield chemical shifts of ca. 12–15 ppm compared with the corresponding unsilylated congener. As observed with the phosphorothiolate monoesters, the presence of excess BSA in solutions of nucleoside phosphoroselenolate monoesters enabled these materials to be stored under anhydrous conditions with minimal degradation over 24 h.

**Figure 4:**
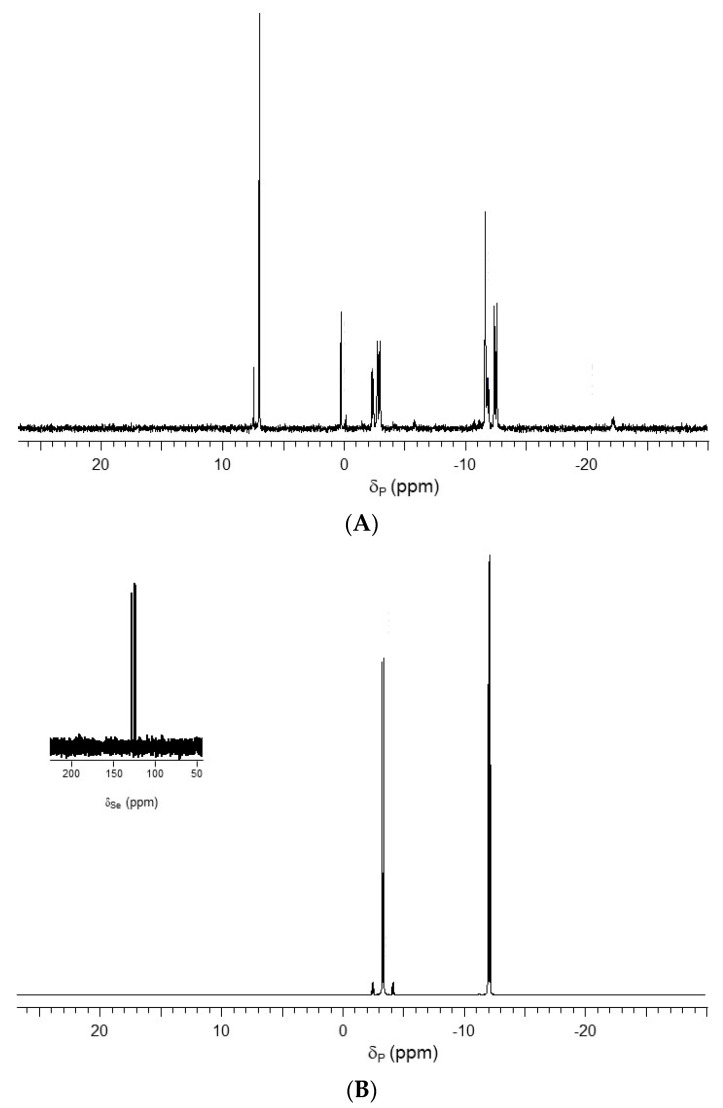
(**A**) ^31^P{^1^H} NMR of crude ball mill reaction mixture of dTSeppA (**2b**); and (**B**) ^31^P{^1^H} and (inset) ^77^Se NMR of **2b** following purification and desalting.

The MA reaction mixtures were subsequently transferred to zirconia-lined vessels, the volatiles removed *in vacuo* and argon was used to equilibrate to atmospheric pressure. In quick succession, acid promoters, water and adenosine 5′-phosphoromorpholidate were added followed by a single zirconia ball to each vessel which was sealed. The vessels were vibrated at 30 Hz for 90 min and allowed to cool to room temperature. Silyl ether functions were cleaved and crude reaction mixtures removed from the vessel as suspensions following washing with water and methanol. The solutions were filtered and immediately analysed by ^31^P NMR (e.g., Figure 4A). Gratifyingly, we observed a new doublet with ^77^Se satellites at δ~−3 ppm accompanied by a doublet at δ~−12 ppm corresponding to the β- and α-phosphoryl groups (of dTSeppA), respectively. A minor peak at δ~7.5 ppm assigned to unconsumed 5′-dTSeMP (**4b**) was found to rapidly disappear upon storage and no related resonance associated with 5′-dASeMP was observed during the synthesis of dASeppA. As found during coupling of the phosphoromorpholidate **8** (AMP-M) with phosphorothiolate monoesters [14], several side products were evident including unreacted **8** (δ 7.0 ppm), AMP (δ~0 ppm), Ap_2_A (δ −11.7 ppm) and the two diastereoisomers of a homocoupled pyrophosphoromorpholidate product Ap(M)pA (δ~−2 ppm and δ~−12 ppm). 

The solutions were reduced in vacuo and then purified using reversed-phase chromatography. The selenium analogues were found to be more hydrophobic than the corresponding thiolates and in order to separate **1b** or **2b** from Ap(M)pA, a gradient using ion pair buffers was employed. During subsequent desalting, the pure materials remained stable during multiple rounds of coevaporation using water. ^31^P NMR analysis of dTSeppA (Figure 4B) showed two doublets (^2^*J*_PP_ 32 Hz) at δ −3.3 ppm (P_β_) and −12.1 ppm (P_α_). The ^77^Se satellites derived from P_α_-Se coupling (^1^*J*_PSe_ 415 Hz) appears intermediate between that observed for internucleotide phosphoroselenolate diesters (ca. 390 Hz) and that observed with triesters (ca. 490 Hz).

### 2.2. Stability of Pyrophorophorochalcogenate-Linked Dinucleosides

**Figure 5:**
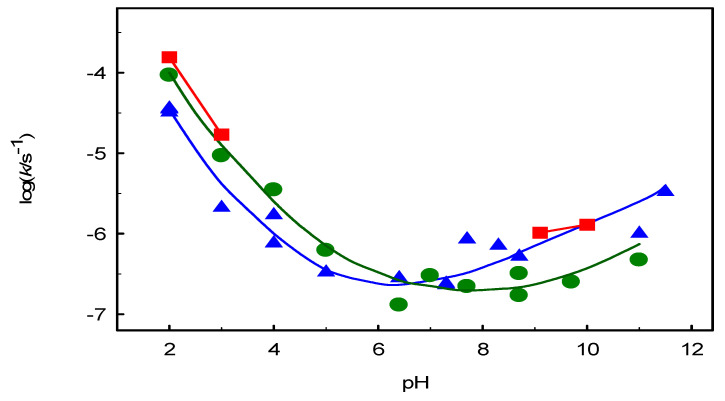
Rate constants for the total disappearance of dinucleoside pyrophosphates **1a** (dASppA: green circles), **1b** (dASeppA: red squares) and **3** (3′,5′-dTSppA: blue triangles) as a function of pH at 363 K. The lines are manually drawn to emphasise the trend.

Reactions of pyrophosphates dASppA (**1a**) and 3′,5′-dTSppA (**3**) were studied at 90 °C. Samples taken from reaction solutions were analysed by two methods: capillary zone electrophores (CZE) and reversed-phase HPLC (RP-HPLC), for detection of polar and neutral reaction components, respectively. The results in Figure 5 show that the thiolate substitution does not significantly enhance the reactivity of pyrophosphates studied under any conditions: Rate constants obtained for **1a** and **3** are of the same order as those reported earlier for diadenosine-5′,5′-di (**1c**) and triphosphate (**5**) [33]. In general, the reactivity of **1a** and **3** is low; the half-lives under neutral conditions are measured in tens of days even at 90 °C. The rate of the total disappearance of thiopyrophosphates **1a** and **3** is practically pH-independent between pH 6 to 9. The reactivity increases at a lower pH; under alkaline conditions, the rate-enhancement is more modest.

The reactivity of the selenopyrophosphate **1b** is not significantly different from that of **1a** and **3** under acidic and slightly alkaline conditions. As will be discussed later, comparable rate constants for the disappearance of **1b** could be obtained only under these conditions since between pH 3 and 9, the reaction did not follow first-order kinetics.

**Scheme 2:**
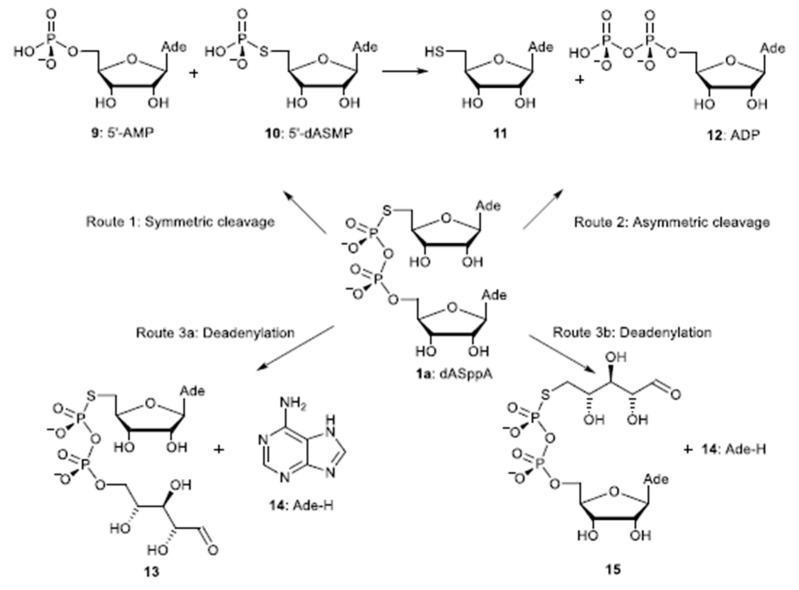
Potential decomposition routes of dASppA (**1a**).

**Scheme 3:**
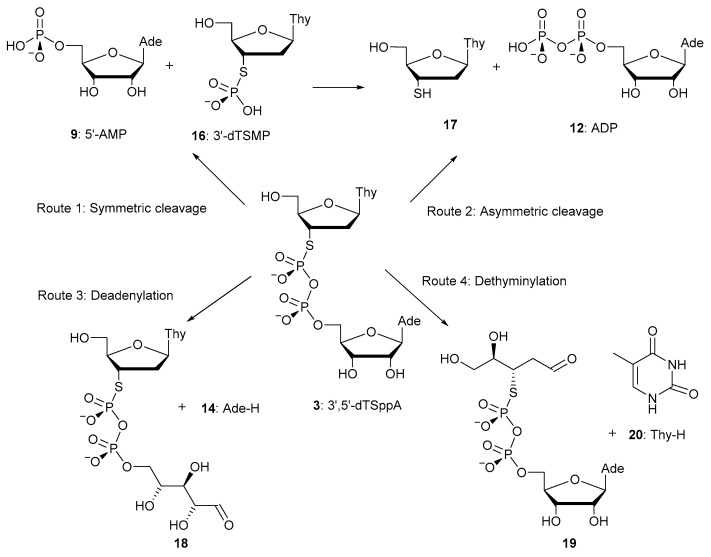
Potential decomposition routes of 3′,5′-dTSppA (**3**).

At pH 2.0 both thiopyrophosphates **1a** and **3** decompose by the hydrolysis of the phosphate bridge (Scheme 2 (dASppA) and **3** (3′,5′-dTSppA), Routes 1 and 2) and of the *N*-glycosidic bond in adenosine or 5′-thioadenosine (Scheme 2 (Routes 3a,b) and Scheme 3 (Route 3)). Route 1 in Scheme 2 describes a symmetric cleavage between the two phosphate groups to yield two monophosphates 5′-AMP (**9**) and 5′-dASMP (**10**). Asymmetric cleavage (Route 2) has not been observed with the oxygen analogue Ap_2_A (**1c**) [33], but considering the better leaving group properties of thionucleoside **11** in comparison to nucleosides, and appearance of ADP (**12**) as a minor reaction product, it is plausible in this case. According to the product analysis with RP-HPLC, the predominant neutral product is adenine (**14**), which shows that under these conditions, deadenylation (Routes 3a and 3b) significantly contributes to the total reactivity.

Polar products observed by CZE in the reaction of **1a** were 5′-AMP (**9**), ADP (**12**), and a product that was tentatively assigned as the depurinated compound **13**. As will be discussed later, only the *O*-linked sugar nucleotide **18** was observed with **3** at pH 3 and above, where two such products (**18** and **19**) could have been expected. 5′-dASMP (**10**) which is initially formed as the other product in the symmetric cleavage, is not observed, as it is rapidly hydrolysed to the corresponding thionucleoside **11**. As is shown by the results collected in Table 1, phosphorothiolate monoester **4a** is hydrolysed within hours under acidic conditions even at 25 °C. Taking the temperature difference into account, up to 10^5^-fold rate difference between dinucleoside pyrophosphates and nucleoside thiolate monophosphates can be estimated. 

The rates observed for the hydrolysis of sulfur and selenium-substituted phosphate monoesters follow the trend described by previous workers [34,35] for *O*-acyl and *O*-aryl phosphate monoester dianions. In these earlier studies, a linear relationship between the log(k) values and pK_a_ of the leaving group was observed and consistant with the more acidic nucleoside selenol, a forty-fold rate enhancement in P-Se cleavage rate is observed compared with P-S cleavage. 

Evidence for a dissociative transition state can be seen in the Eyring parameters derived from variable temperature NMR analysis of the phosphorus-chalcogen bond cleavage kinetics at 29 8K, 308 K and 318 K under neutral pH conditions. Thus, positive ΔS^‡^ values of 79 and 35 J mol^−1^ K^−1^ were determined for 5′-dTSMP (**4a**) and 5′-dTSeMP (**4b**), respectively. Reflecting the relative strengths of the P-Ch bond, a higher ΔH^‡^ was observed in 5′-dTSMP (126.5kJ mol^−1^) than that in 5′-dTSeMP (110.6 kJ mol^−1^). 

**Scheme 4:**
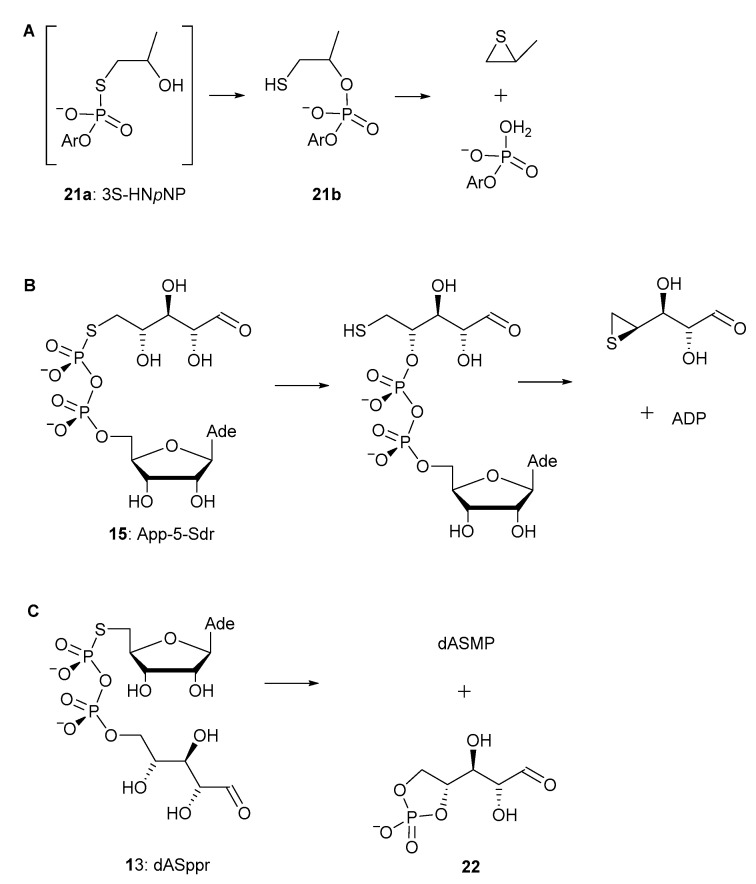
(**A**) Model reaction of 3*S*-HN*p*NP (**21**, Ar = 4-nitrophenyl) [36]; (**B**) Proposed transesterification of App-5-Sdr (**15**) and subsequent pyrophosphate cleavage; (**C**) Proposed cyclisation of dASppr (**13**).

It was surprising that the *S*-linked sugar nucleotides **15** and **19** were not observed. Results obtained with other phosphorothiolate compounds offer, however, a potential explanation. Products **15** and **19** can be contrasted with the reactivity of model thiolate phosphodiesters, such as Iyer and Hengge’s 3*S* phosphorothiolate analogue of HP*p*NP (Scheme 4A), with a flexible nucleophile and a good leaving group [36]. The model 3*S*-HP*p*NP (**21a**) undergoes rapid isomerisation to the corresponding 2*O* phosphate diester **21b** followed by a nucleophilic attack of the thiol on the vicinal carbon resulting in a formation of a thiirane ring and the loss of the phosphate group [36]. *S*-Linked sugar nucleotides **15** and **19** contain the same structural elements, and a similar reaction route, depicted in Scheme 4B, would result in the rapid decomposition of these products. In the case of **15** or **19**, the reaction would result in the release of ADP that is subsequently hydrolysed to 5′-AMP. Thus, ADP observed as a reaction product may be formed also by the decomposition of intermediates **15** and **19**, and is not conclusive evidence of the asymmetric cleavage of the pyrophosphate bridge.

The absence of *S*-linked sugar nucleotides could, in principle, result also from an exceptional stability of an *N*-glycosidic bond in 5′-thionucleoside derivatives. This alternative does not, however, seem likely as deadenylation of 5′-thioadenosine has been observed under mildly acidic conditions [37]. 

The *O*-linked deadenylation product **13** is a thioanalogue of a reducing sugar nucleotide ADP-ribose, which we have studied previously [38]. ADP-ribose and related sugar nucleotides react by intramolecular substitution at the α-phosphate to form a 4,5-cyclic ribose phosphate (**22** in Scheme 4C) and a nucleoside monophosphate [38,39]. In the case of **1a** the deadenylated product **13** decomposes to yield 5′-dASMP (**10**) as an initial product as is shown in Scheme 4C. With **3**, the corresponding reaction gives 3′-dTSMP (**16** in Scheme 3). As mentioned above, **10** and **16** are rapidly hydrolysed to corresponding thionucleosides **11** and **17**, respectively.

The reaction systems are kinetically complicated, and the only product that accumulates in the reaction of **1a** and can confidently be assumed to be formed by one route only, is deadenylation product **13**. Kinetics of parallel and consecutive reactions were applied to its formation, and rate constants of 1.9 × 10^−5^ and 3.1 × 10^−5^ s^−1^ were obtained for the formation and decomposition, respectively. These values are consistent with those reported for the deadenylation of Ap_3_A [33] and decomposition of ADP-ribose [38]. Assuming that the two deadenylation reactions (Routes 3a and 3b) are equally fast, the rate constant of the total disappearance (9.3 × 10^−5^ s^−1^) can be divided into those of deadenylation and phosphate hydrolysis. The value of 5.5 × 10^−5^ s^−1^ obtained for the latter reaction is only six times larger than that reported for the hydrolysis of Ap_2_A (**1c**) under the same conditions [33]. Since it is possible that ADP is formed through the pathway described in Scheme 4B, it is not possible to evaluate to what extent the pyrophosphate bridge is cleaved by the asymmetric route (Scheme 2, Route 2).

Reactions of 3′,5′-dTSppA (**3**) are shown in Scheme 3. Similarly to dASppA, **3** decomposes at pH 2 by the hydrolytic cleavage of the pyrophosphate bridge and the *N*-glycosidic bond. Adenine was the most abundant product observed in the HPLC analysis suggesting that deadenylation is the predominant reaction pathway. CZE-analysis shows the formation of two polar products, one of which was identified as 5′-AMP. The other polar product most probably is the deadenylation product **18.** The migration time in CZE analysis was consistent with a diphosphate containing product, and a UV-maximum of 270 nm indicated that the UV-absorbing moiety is thymine. Applying the kinetics of parallel and consecutive reactions gave rate constants of 2.3 × 10^−5^ s^−1^ and 2.0 × 10^−5^ s^−1^ for the formation and decomposition of the alleged depurination product. These values are consistent with those obtained for the corresponding product on the reaction of **1a**, which lends further support to the product assignment.

The other significant reaction pathway is most probably the symmetric phosphate hydrolysis producing 5′-AMP and 3′-dTSMP (Route 3 in Scheme 3). 3′-dTSMP was not directly observed, because of its rapid hydrolysis to the corresponding thionucleoside 3′-dTSH **17** which undergoes oxidation to the corresponding symmetrical disulfide (3′-dTS)_2_. Consistent with this, a thymine-containing product is observed at a clearly longer retention time. The identity of products assumed to be **17** and its oxidised dimer was confirmed with HPLC-MS-analysis. ADP is not observed among the reaction products, but the possibility of asymmetric phosphate hydrolysis as a minor reaction route cannot be strictly excluded, as the hydrolysis of ADP under acidic conditions is faster than that of **2** [40]. 

As the pH increases the rate of total disappearance of both dASppA (**1a**) and 3′,5′-dTSppA (**3**) decrease. At pH 3, deadenylation is still the predominant reaction pathway with both substrates. However, a slow dethyminylation (Scheme 3, Route 3b) is also observed with **3**; thymine was identified by spiking with authentic compound in RP-HPLC analysis. At pH 4, dethyminylation and deadenylation (Scheme 3, Routes 3b and 3a) are equally fast processes, based on the concentrations of adenine and thymine formed. Only one sugar nucleotide product was observed under these conditions, and the UV-spectrum was consistent with the thymine containing *O*-linked product **18**. 

The reactivity minimum is reached under neutral conditions, where the half-life of the total disappearance of **1a** and **3** is measured in tens of days at 90 °C. The rate constant for the disappearance of **1a** is approximately the same as that obtained for **5** before [33], showing that the effect of thiosubstitution is very modest. The only polar product significantly accumulating in either reaction is 5′-AMP. The decomposition of sugar nucleotides is base-catalysed at neutral pH [38], and *O*-linked sugar nucleotides **13** and **18** are likely to be approximately 100 times more reactive than **1a** or **3** under neutral or slightly alkaline conditions. The RP-HPLC analysis shows also that a significant amount of thymine is formed in the reaction of **3**. A rate constant of (2.2 ± 0.2) × 10^−7^ s^−1^ was obtained for dethyminylation by a non-linear regression applied to the formation of thymine. Thymine may be released at different stages of the reaction, and, hence, this method is not theoretically fully correct, and the value obtained is, therefore, rather approximate. It is, however, consistent with that reported by Ora et al. [41] under the same conditions. The value obtained suggests that dethyminylation is the predominant reaction of **3** at neutral pH. 

In addition to thymine, the only neutral products observed under neutral conditions were adenosine, thionucleosides **11** and **17**, and their oxidized dimers. The product distributions thus give little information on the reaction routes. In the absence of any evidence for other processes, it can be proposed that **1a** decomposes by the symmetric cleavage of the pyrophosphate bridge (Scheme 2, Route 1). In the case of **3**, the symmetric phosphate cleavage is accompanied by dethyminylation that is the predominant reaction pathway. 

The rate of the total disappearance of **1a** and **3** increases only modestly as the pH increases from 7 to 10. However, a number of additional polar products at a low concentration were observed. These products can be attributed to two reactions that have been observed previously with Ap_3_A (**5**) under slightly alkaline conditions [33]: intramolecular nucleophilic attack by 3’-OH on the phosphate group resulting in a formation of adenosine 3′,5′-cyclic monophosphate [42], and a base-catalysed opening of the imidazole ring [43] in the adenine base that eventually leads to the release of adenine. 

The seleno analogue dASeppA (**1b**) was studied less thoroughly because it appeared that the reaction system was much more complicated than in the case of **1a** and **3**. Under acidic conditions the decomposition of **1b** followed first-order kinetics and the rate constant of the total disappearance (1.55 ± 0.04) × 10^−4^ s^−1^ at pH 2 was obtained for **1b**. This value is only slightly higher than that obtained for the thioanalogue **1a** under the same conditions. 

As the pH increased, the total disappearance of **1b** no longer followed first-order kinetics, but the rate of disappearance increased as the reaction proceeded. The ln *x***1b**) vs. time plots were typically linear in the beginning of the reaction, but after a short period the reaction rate began to increase. The phenomenon was most pronounced under neutral conditions. The initial linear plot gave rate constants that were 5 to 20 times larger than those obtained for **1a**. The difference was largest under neutral conditions. The behaviour was tentatively attributed to a formation of a reactive seleno species. It is known that compared to sulfur, the chemistry of selenium is much more diverse. Selenols are good leaving groups, and also good nucleophiles, particularly under neutral conditions [44]. In additions to selenols, other selenium compounds are nucleophilic, as well, and can induce phosphate diester cleavage [45]. 

**Figure 6:**
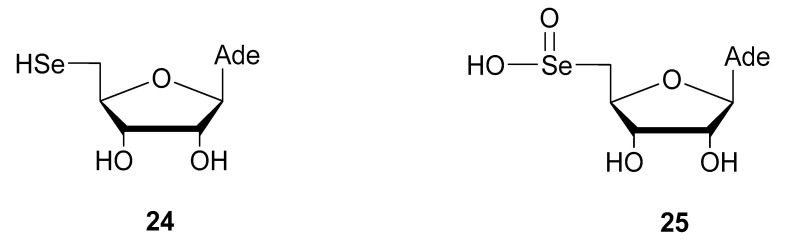
Se-containing products formed in the hydrolysis of dASeppA (**1b**).

However, we failed to detect any reactive intermediates or reaction products resulting from their reactions. The product analysis offered little information to explain the unexpected behaviour. 5′-AMP, adenosine, selenonucleoside **24** (in Figure 6) and adenine were the predominant products observed under slightly acidic and neutral conditions. Under slightly basic conditions a new peak, that was identified as a seleninonucleoside **25** by HPLC-MS analysis, was observed as one of the products. At pH 11, 5′-AMP and **25** were the main products observed.

### 2.3. pKa Titrations and SVPDE Cleavage of Pyrophosphorochalcogenolate-Linked Dinucleosides

In order to further assess the potential for chalcogen-substituted pyrophosphate linkages to enable targeted drug delivery, the p*K*_a_ values of the phosphoryl moieties were assessed using ^31^P NMR titration. Upon acidification of aqueous solutions of dASppA and dASeppA, upfield shifts of ca. 0.4 ppm were apparent in the P_β_ resonances (Supplementary Materials) from which the p*K*_a_ ‘s were calculated as 3.0 (**1a**) and 3.3 (**1b**). Similar values were determined for dTChppA (3.2 for **2a**; 3.4 for **2b**) although maximal shifts of 0.2 ppm were observed and a second inflection at ca. pH 8.5 may indicate that the shifts reflect nucleobase ionisation [46] as significant intramolecular base stacking occurs in AppA and other dinucleoside pyrophosphates [47]. However, the titrations’ inflections represent a maximum p*K*a value and therefore indicate that these moieties will be fully charged at physiological pH. We therefore briefly compared the cleavage of these pyrophosphates using snake venom [48] monitoring the reactions of NADH, dTSppA (**2a**) and dTSeppA (**2b**) by ^31^P NMR. Over 95% consumption of the native pyrophosphate substrate was observed consumed within one hour. In contrast, ca. 25% and 20% of the sulfur- and selenium-substituted analogues were cleaved during this timeframe. Continued divergence between the substrate behaviours of **2a** and **2b** was found over the subsequent 14 h during which time, complete consumption of dTSppA occurred. In contrast, digestion of dTSeppA plateaued at ca. 45% which may result from denaturation of the protein following release of 5′-dTSeMP and subsequent spontaneous hydrolysis to the corresponding selenol and its subsequent interaction with the active site zinc dyad and disulfide linkages [49]. 

## 3. Discussion

The results provide insight on the reactivity of the novel pyrophosphates studied and on other types of thiosubstituted biological phosphates. It is clear that the reactivity of dinucleoside pyrophosphates is influenced only modestly when the bridging 5′- or 3′-oxygen is substituted by sulfur. The modest effect is somewhat surprising considering that with phosphodiester substrates a substitution of sulfur for the remnant group oxygen (e.g., 3′-oxygen in RNA model dISpU), may significantly enhance the nucleophilic substitution at phosphorus, particularly under neutral conditions [29,30,31]. In those cases the rate enhancement has been attributed to enhanced nucleophilic attack on the phosphorus, or to the properties cyclic thiophosphorane intermediate. The fact that significant rate enhancement is not observed in this work, when an intermolecular nucleophilic attack takes place, and the reaction proceeds through an acyclic phosphorane, is consistent with the latter alternative. Thiosubstitution as such does not, hence, significantly enhance the nucleophilic attack on a phosphate group.

Hydrolysis of the *N*-glycosidic bond of **1a** and **3** yields products that are thioanalogs of reducing nucleotide sugars, such as ADP-ribose. As can be expected, thiosubstitution at the leaving nucleotide does not affect the reactivity as is shown by the rate constant for the decomposition of **13**. In contrast to that, the thiolinked sugar nucleotides **15** and **19** are apparently significantly more reactive than their natural counterparts. The reaction most likely involves a phosphate migration from sulfur to oxygen and a nucleophilic attack by a thiol group on the carbon resulting in a formation of a thiirane ring and release of a phosphate group as shown in Scheme 4A. Such a reaction seems to be possible only when the nucleophile is flexible and there is a good leaving group in the molecule. Ribonucleoside derivatives [29,30,31] or compounds with a poor leaving group [36] do not react similarly.

Similarly to other phosphorylated thiols [50,51], thionucleoside monophosphates are several orders of magnitude more reactive than their natural counterparts. The reactivity difference between 5′-dTSMP (**4a**) and 5′-TMP (**4c**) at pH 3 was estimated to be approximately 700,000-fold on the basis of results in Table 1. The pH-dependence of the reactions are similar: the reactivity decreases as the pH increases. It is commonly accepted, that the dephosphorylation is a unimolecular process that results in the release of a resonance stabilised metaphosphate anion [52,53,54]. The large reactivity difference between μ-monothiopyrophosphate and pyrophosphate has been attributed to the weakness of a P-S bond that allows the release of a metaphosphate anion [53,55]. The stability of the metaphosphate monoanion apparently plays a significant role; dinucleoside pyrophosphates studied in the present work do not react, to any significant extent, by the cleavage of a P-S bond.

The inherent reactivity of the selenium substituted pyrophosphate dASeppA (**1b**) or nucleotide 5′-dTSeMP (**4b**) are not markedly different from that of their thioanalogues. The irregular kinetics observed with **1b** between pH 3 and 9 most likely result from a reaction between an intact pyrophosphate and a reactive selenium species formed as a secondary product. The hydrolysis of selenonucleoside monophosphate **4b** is faster by several orders of magnitude than that of **1b** and, hence, similar behaviour was not observed. However, the pH-dependence of the hydrolysis of the selenonucleotide **4b** is different from that of the thio (**4a**) and oxo (**4c**) analogues suggests, however, that the metaphosphate ion release is not the only reaction route with the selenonucleotide. The less positive activation entropy may well reflect this difference, as well. 

## 4. Materials and Methods

Kinetic experiments with dinucleoside pyrophosphates **1a,b** and **3** have been carried out using methods that have been reported previously [33,38], and are only briefly described below. Other experimental methods are described in detail in the Supplementary Materials (Figures S1 and S2, Tables S1 and S2).

Capillary zone electrophoretic (CZE) analysis was carried on an HP ^3D^CE or on a Beckman Coulter P/ACE MDQA equipment. A fused silica capillary (57 cm effective length, 75 μm i.d.) was used. The background electrolyte was a 25 mM potassium phosphate buffer (pH 7.2). A voltage of 25–30 kV was applied and the compounds were detected at 260 nM (HP) or 254 nm (Beckman).

RP-HPLC analysis was carried on an Agilent 1100 HPLC equipped with a DAD-detector. A Supelcosil LC-18 column (25 × 0.4 cm, particle size 5 μm) was used to separate reaction components. Eluents were mixtures of acetonitrile (MeCN) (Honeywell, Germany) and acetate buffer (0.05 M, pH 4.3) containing 0.1 M ammonium chloride (Sigma-Aldrich, Seelze, Germany). The gradient program used in routine analysis was as follows: 0–10 min isocratic 5% MeCN, 10–20 min a linear gradient to 33% MeCN, 20–30 min isocratic 33% MeCN, 30–35% a linear gradient to 5% MeCN. Flow rate was 1 mL/ min. UV-active compounds were detected at 260 nm.

HPLC-MS –analysis was carried out on an Agilent 1260 Infinity HPLC coupled with 6120 Quadrupole mass analyzer. The column was Supelcosil C18 (25 × 0.4 cm, particle size 5 μm), and the eluents mixtures of 5 mM ammonium acetate and acetonitrile. 

Rate constants were calculated by applying the integrated first-order rate law to the disappearance of the starting material. Rate constants for the formation and decompositions of products were calculated by applying the rate law of parallel and consecutive reactions [33,38]. In the case of 2, the concentration of the starting material and products were determined using calibration curves based on authentic samples. Diphenyl phosphate was used as an internal standard in reactions. 

## 5. Conclusions

The results obtained in the present study show that the effect of substitution of sulfur for a phosphate oxygen depends significantly on the structure of a biological phosphate compound, and consequently, on the reaction mechanism. While the hydrolysis of thionucleoside monophosphates, that involves a cleavage of a P-S bond and a release of a metaphosphate anion, is several orders of magnitude faster than that of nucleoside monophosphates, the reactivity difference between corresponding dinucleoside pyrophosphates is very modest. In addition to the known reactivity of phosphorothiolate diesters, structures with a flexible intramolecular nucleophile and a good leaving group decompose rapidly as a result of a phosphate migration and a nucleophilic attack of thiol on the neighbouring carbon.

The reactivity of selenium substituted analogues does not seem to be inherently significantly different from that of corresponding sulfur compounds, but it seems that in the former case, reactive intermediates that react with intact substrates, may be formed, contributing to their overall reactivity. In considering the potential for biochemical unmasking of pharmacologically active selenols following the action of *extracellular* pyrophosphatases and phosphatases, the observed attenuation of SVPD activity may point to the potential for inhibiting ENPP—recently identified as a target in treating relapsing breast cancer [56]. 

The chemical stability of the pyrophosphate substrates studied is promising considering the potential therapeutic or diagnostic applications. However, the results show also that the chemistry of thiosubstituted biomolecules is more diverse than that of their natural counterparts, and unexpected reactivity may be encountered with new types of compounds. The effects of selenium substitution seem to be much more drastic in this respect but we anticipate that by identifying the products of chemical hydrolysis of these materials in the current study, we will be able to better understand the metabolism of from related materials currently under investigation in ongoing studies in vivo.

## Figures and Tables

**Table 1:** Rate constant for the hydrolysis of nucleotides under acidic, neutral and alkaline conditions.

Substrate	Conditions	*k*/10^−6^ s^−1^
5′-dTSMP (**4a**)	pH 3, 298 K	152
	pH 7, 298 K	5.19
	pH 7, 308 K	17.1
	pH 7, 318 K	58.4
	pH 11, 298 K	0.34
5′-dTSeMP (**4b**)	pH 3, 298 K	53.1
	pH 7, 298 K	16.8
	pH 7, 303 K	75.1
	pH 7, 313 K	297
	pH 11, 298 K	14.4
5′-TMP (**4c**)	pH 3, 363 K	2.60

## Data Availability

The data presented in this study are available in the article and in the Supplementary Materials.

## Supplementary Materials

The following supporting information can be downloaded at https://www.mdpi.com/article/10.3390/ijms232415582/s1.
