# Supplementary material for: 5′-Chalcogen-Substituted Nucleoside Pyrophosphate and Phosphate Monoester Analogues: Preparation and Hydrolysis Studies
Source: Int J Mol Sci. 2022 Dec 8;23(24):15582. doi: 10.3390/ijms232415582 (PMC9778984; doi:10.3390/ijms232415582)
Supplement: Supplementary file 1 [file ijms-23-15582-s001.zip › ijms-2038105-supplementary.pdf]

Electronic Supplementary Information (ESI)

5'-Chalcogen-substituted nucleoside pyrophosphate and phosphate monoester analogues: preparation and hydrolysis studies

Satu Mikkola [1]*†, Olga Eguaogie [2] †, Anu Nieminen [1], Patrick F. Conlon [2,3], David L. Jakeman [3], Keith Moore [2], Ian C. Lane [2], and Joseph S. Vyle [2]

[1] *Department of Chemistry, University of Turku, FIN-20014 Turku, Finland.*
[2] *School of Chemistry and Chemical Engineering, Queen's University of Belfast, David Keir Building, Stranmillis Road, Belfast BT9 5AG, UK.*
[3] *College of Pharmacy, Dalhousie University, Halifax, Nova Scotia B3H 4R2, Canada.*

E-mail: [1] satkuu@utu.fi; [2] j.vyle@qub.ac.uk

General information .... - 2 -

Experimental procedures and material characterisation .... - 4 -

5′-deoxy-5′-selenoadenosine 5′-pyrophosphate (P′ →5′) adenosine (1a) - dASeppA .... - 4 -
5'-thiothymidine 5'-pyrophosphate (P' →5') adenosine (2a) – dTSppA .... - 5 -
5′-deoxy-5′-selenothymidine 5′-pyrophosphate (P′ →5′) adenosine (2b) - dTSeppA .... - 6 -
5′-deoxy-5′-(5-nitropyridyl-2-disulfanyl)thymidine (6a) –NPySSdT .... - 7 -
pH titrations and pKa determination of pyrophosphorochalcogenate-linked dinucleotides .... - 7 -
dT(Ch)MP degradation studies .... - 8 -

Raw analytical data .... - 9 -

M-A reaction of NCSedA (5) .... - 9 -
NPySSdT – 6a .... - 43 -
Table 2: Model fits to ^{31}P NMR data in nucleoside compounds dAChppA .... - 46 -
Table 2: Model fits to ^{31}P NMR data in nucleoside compounds dTChppA .... - 47 -
Table S2: Summary of first order .... - 51 -
Snake venom digestion: .... - 53 -

General information

Reagents or solvents were purchased from commercial suppliers (Aldrich or Fluka) and used without further purification unless otherwise stated. Discussions in the text are based upon the use of *N*,*O*-bis(trimethylsilyl)acetamide (Aldrich 128910) which was 95% pure. Anhydrous chloroform and $CDCl_3$ were dried by storing over phosphorus pentoxide and subsequently filtered under gravity through activated basic alumina immediately prior to use. Otherwise, acid-free chloroform was obtained following filtration through activated basic alumina immediately prior to use. Dichloromethane was distilled from calcium hydride and stored in the absence of light over activated 3Å molecular sieves for no more than one week prior to use.
Davisil silica gel 60 Å was used for flash chromatography. TLC was performed using Merck Kieselgel 60 F254 plates and materials visualized using UV (254nm) illumination, 0.1% (w/v) Ellman's reagent in 1:1 EtOH : aqueous 0.45 M Tris·HCl (pH 8.5) (for (thiol(ate)s, selenol(ate)s) and 3% (w/v) phenol in 95:5 (v/v) ethanol:conc. H_2SO_4 (for sugar-containing materials). Where appropriate, the plates were subsequently heated at high temperature (*ca.*100 – 200 °C). Whatman 13 mm diameter, 0.45µm pore size syringe filters with polypropylene housing (Aldrich WHA 67831304) were used for chalcogen nucleoside monophosphate degradation studies and snake venom (*Crotalus atrox* (Western Diamondback Rattlesnake)) (Sigma - V700) was used for digestion of pyrophosphorochalogenolate substrates.

All ball mill reactions were performed using a Retsch Mixer Mill MM 400[1] using a 15mm zirconia ball (10.70 g) [in a zirconia-lined vessel (25mL internal volume)] according to the conditions described below.

<u>HPLC</u>

HPLC was performed on a ThermoFinnigan SpectraSYSTEM modular HPLC system consisting of a P2000 binary gradient pump and UV1000 sample detector. Samples were injected manually via a Rheodyne injection valve. The HPLC was interfaced via an SN4000 controller (Thermo Scientific) to a Windows PC running ChromQuest 5.0 data acquisition software (Thermo Scientific).
Buffers were prepared using H_2O purified to 18.2 MΩ by reverse osmosis (Barnstead NANOpure Diamond water purification system), acetonitrile (Aldrich 34851) triethylamine (Aldrich 471283), acetic acid (Aldrich 320099), tetrabutylammonium hydrogen sulfate (TCI I0368) and CO_2 generated by sublimation of the solid compound.
Analytical HPLC was performed using a Phenomenex Clarity 5µm Oligo-RP (150 x 4.60 mm) column eluting at 1 mL min^{-1}, monitoring at 260 nm using gradients G1.
Preparative HPLC was performed using a Phenomenex Clarity 5µm Oligo-RP – (250 x 21.2 mm) column eluting at 8mL min^{-1}, monitoring at 280 nm using gradients G2 and G3.
Ion pair (IP) buffers were prepared from solutions containing a mixture of tetrabutylammonium hydrogen sulfate (final concentration 6 mM) and acetic acid (final concentration 30 mM) in H_2O following neutralisation with triethylamine to pH 6.3 and suitable dilution with pure water (Buffer A) or to give 50% (v/v) MeCN (Buffer B).

Desalting (TEAB) buffers were prepared from 1 M stock solutions of triethylammonium bicarbonate in H_2O. These were prepared by bubbling CO_2 through a sintered frit into a mixture of triethylamine and H_2O at 0°C to give homogenous solutions. Stock solutions were stored at 4°C until required (up to 2 days) and then further diluted as required to give: 100 mM TEAB (aq.), pH 7.8 (Buffer A); or 100 mM TEAB in 65:35 (v/v) MeCN:H_2O, pH 8.2 (Buffer B).

Gradient G1 (analytical; IP buffers): 0-5 min, 20% Buffer B; 5-25 min, 20-60% Buffer B; 25-30 min, 60% Buffer B; 30-35 min, 60-20% Buffer B; 35-45 min, 20% Buffer B.

Gradient G2 (preparative; IP buffers): 0-13 min, 20% Buffer B; 13-63 min, 20-60% Buffer B; 63-75 min, 60% Buffer B; 75-83 min, 60-20% Buffer B; 83-113 min 20% Buffer B.

Gradient G3 (preparative; TEAB buffers): 0-10 min, 0% Buffer B; 10-60 min, 0-30% Buffer B; 60-80 min, 30-100% Buffer B; 80-90 min, 100% Buffer B; 90-110 min, 100-0% Buffer B; 110-120 min, 0% Buffer B.

^{1}H, ^{13}C or ^{31}P NMR spectra were recorded on a Bruker III-400 MHz or 600 MHz at 300K. ^{77}Se NMR (proton-decoupled) were recorded on a Bruker Ascend-600 MHz at 300 K using an insert containing 0.25 M KSeCN in D_2O as external standard (−329.00).

Mass spectra were recorded using a VG Quattro II Triple Quadrupole Mass Spectrometer (Electrospray). Mass spectrometry was performed by Analytical Services and Environmental Projects (ASEP) at Queen's University Belfast.

Experimental procedures and material characterisation

5′-deoxy-5′-selenoadenosine 5′-pyrophosphate (P′ →5′) adenosine (1a) - dASeppA

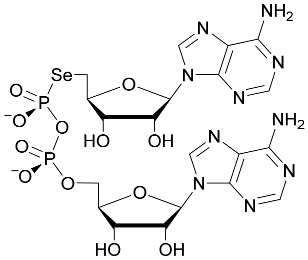

A suspension of 5′-deoxyadenosinyl-5′-selenocyanate (Ref. 32 in the main text) (**5**) (53.0 mg, 0.15 mmol) in 4:1 anhydrous chloroform:BSA (2.0 mL) under argon was sonicated for 20 minutes after which time a clear solution had formed and was left to stir at ambient temperature under argon for a further 30 minutes. To this stirred solution was added a solution of $(TMSO)_3P$ (55.0 µL, 0.165 mmol, 1.1 eq.) in 4:1 anhydrous chloroform:BSA (0.65 mL) and these conditions maintained for 18 hours. ^{31}P NMR indicated complete reaction and the solution was left overnight at room temperature under inert conditions. The reaction mixture was analysed by ^{31}P NMR. The reaction mixture was then transferred into a zirconia-lined vessel under argon and the residues rinsed from both the reaction flask and NMR tube were rinsed with anhydrous chloroform (2 x 0.1 mL). The vessel was stored at ambient temperature in a desiccator under vacuum for four hours until volatiles had been removed and the residue had the consistency of a paste. After equilibrating to atmospheric pressure with argon, the jar was charged sequentially with AMP-morpholidate (**5**) (319 mg, 0.45 mmol, 3.0 eq.), tetrazole (22 mg, 0.315 mmol, 2.1 eq.), $MgCl_2•(H_2O)_6$ (46 mg, 0.225 mmol, 1.5 eq.), H_2O (32.0 µL, 1.80 mmol, 12 eq.) and a 15 mm zirconia ball. The vessel was sealed and vibrated at 30 Hz for 90 minutes and allowed to cool to room temperature. The crude reaction mixture was extracted from the vessel using both physical fracturing of the solid residues (with a polypropylene automatic pipette tip) and successive rinsing with H_2O (3 x 1 mL) and finally methanol (1 mL) into eppendorf tubes. The combined suspensions were sonicated, filtered in 1 mL aliquots through a Spin-X cellulose acetate centrifuge filter (0.45 µm) at 12,000 rpm and the solids rinsed with H_2O (0.5 mL). The combined extracts were immediately analysed by ^{31}P NMR. The reaction mixture was purified by C18 RP-HPLC using IP buffers (gradient G2) from which a single peak corresponding to pure dASeppA (**6**) was collected. Combined pure fractions were concentrated *in vacuo* and subject to desalting following HPLC isolation using TEAB buffers (gradient G3) and repeated coevaporation with deionised water. Isolated yield of pure: dASeppA •~1.8 (Et_3NH) (**1a**): 1256 OD^{260nm} units (0.050 mmol, 33%). t_R (G1) = 12.4 min.

^1H NMR (600 MHz, D_2O) δ_H = 8.24 (1H, s, H2), 8.03 (1H, s, H2), 7.93 (1H, s, H8), 7.91 (1H, s, H8), 5.90 (1H, d, $^3J_{HH}$ = 5.1 Hz, H1′-rA), 5.80 (1H, d, $^3J_{HH}$ = 5.4Hz, H1′-dA), 4.56 (1H, t, $^3J_{HH}$ = 5.3Hz, H2′-dA), 4.53 (t, 1H, $^3J_{HH}$ = 5.0 Hz, H2′-rA), 4.43 (1H, t, $^3J_{HH}$ = 4.7 Hz, H3′-rA), 4.36-4.29 (3H, m, H4′-dA, H3′-dA, H4′-rA), 4.25-4.18 (2H, m, H5′,H5″-rA), 3.26-3.13 (2H, m, H5′,H5″-dA).

^{13}C NMR (151 MHz, D_2O) δ_C = 154.75, 154.67, 152.30, 152.20, 148.17, 148.15, 139.55, 139.43, 117.97, 117.83, 87.22, 87.09, 83.54 (d, $^3J_{PC}$ = 6.0 Hz), 83.41 (d, $^3J_{PC}$ = 9.1 Hz) 74.70, 74.01, 72.40, 70.01, 65.25 (d, $^2J_{PC}$ = 6.0 Hz), 26.69 (d, $^2J_{PC}$ = 4.5 Hz).

^{31}P NMR (243 MHz, D_2O) δ_P = -3.30 (d, $^2J_{PP}$ = 31.6 Hz; $^1J_{PSe}$ = 409 Hz P_β), -12.02 (d, $^2J_{PP}$ = 28.9 Hz, P_α).

^{77}Se NMR (114 MHz, D_2O, external 0.25M KSeCN standard in D_2O: -329 ppm) δ_{Se} = 137.88 (d, $^{1}J_{PSe}$ = 409 Hz).
HRMS (ESI, negative ion). Calculated m/z for ($C_{20}H_{26}N_7O_{13}P_2Se$) [M + H]$^{-}$: 739.0294, found 739.0340.

5'-thiothymidine 5'-pyrophosphate (P' →5') adenosine (2a) – dTSppA

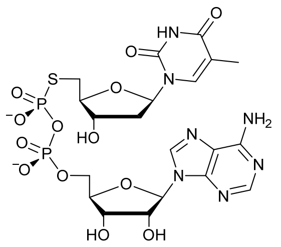

A suspension of 5′-deoxy-5′-(5-nitropyridyl-2-disulfanyl)thymidine (**6a** - see below) (62.0 mg, 0.15 mmol) in 4:1 anhydrous chloroform:BSA (2.0 mL) under argon was sonicated for 20 minutes after which time a clear solution had formed and was left to stir at ambient temperature for a further 30 minutes. To this stirred solution was added a solution of $(TMSO)_3P$ (55.0 µL, 0.165 mmol, 1.1 eq.) in 4:1 anhydrous chloroform:BSA (0.65 mL) and these conditions maintained for 18 hours. The reaction mixture was analysed by ^{31}P NMR. The reaction mixture was transferred into a zirconia-lined vessel under argon and the residues from both the reaction flask and NMR tube were rinsed with anhydrous chloroform (2 x 0.1 mL). The vessel was stored at ambient temperature in a desiccator under vacuum for four hours until volatiles had been removed and the residue had the consistency of a paste. After equilibrating to atmospheric pressure with argon, the jar was charged sequentially with AMP-morpholidate (**5**) (319 mg, 0.450 mmol, 3.0 eq.), tetrazole (22 mg, 0.315 mmol, 2.1 eq.), $MgCl_2$•$(H_2O)_6$ (46 mg, 0.225 mmol, 1.5 eq.), H_2O (32.0 µL, 1.80 mmol, 12 eq.) and a 15 mm zirconia ball. The vessel was sealed and vibrated at 30 Hz for 90 minutes and allowed to cool to room temperature. The crude reaction mixture was extracted from the vessel using both physical fracturing of the solid residues (with a polypropylene automatic pipette tip) and successive rinsing with H_2O (2 x 1 mL), methanol (1 mL) and finally H_2O (1 mL) into eppendorf tubes. The combined suspensions were sonicated, filtered in 1 mL aliquots through a Spin-X cellulose acetate centrifuge filter (0.45 µm) at 12,000 rpm and the solids rinsed with H_2O (0.5 mL). The combined extracts were immediately analysed by ^{31}P NMR. The reaction mixture was purified by C18 RP-HPLC using IP buffers (gradient G2) from which a single peak corresponding to pure dTSppA (**x**) was collected. Combined pure fractions were concentrated *in vacuo* and subject to desalting following HPLC isolation using TEAB buffers (gradient G3) and repeated coevaporation with deionised water. Isolated yield of dTSppA•~1.7 (Et_3NH) (**2a**): 1578 OD^{260nm} units (0.067 mmol, 45%). HPLC retention time t_R (gradient G1) = 13.2 min.

^{1}H NMR (400 MHz, D_2O) δ_H = 8.39 (1H, s, H2), 8.05 (1H, s, H8), 7.19 (1H, s, H6-dT), 5.99 (1H, dd, $^{3}J_{HH}$ = 6.4 Hz, 6.8 Hz, H1′-dT), 5.97 (1H, d, $^{3}J_{HH}$ = 6.0 Hz, H1′-rA), 4.63 (1H, ψt, $^{3}J_{HH}$ = 5.5 Hz, H2′-rA), 4.44 (1H, dd, $^{3}J_{HH}$ = 5.1 Hz, 3.9 Hz, H3′-rA), 4.35 (1H, m, H3′-dT), 4.30 (1H, m, H4′-rA), 4.25-4.14 (2H, m, H5′, H5″-rA), 4.04 (1H, m, H4′-dT), 3.06-2.98 (2H, m, H5′, H5″-dT), 2.23-2.15 (1H, m, H2′-dT), 2.23-2.13-2.05 (1H, m, H2″-dT), 1.66 (3H, s, C<u>H</u>$_3$).

^{13}C NMR (101 MHz, D_2O) δ_C = 165.95, 155.18, 152.51, 151.16, 148.79, 139.86, 136.79, 118.29, 111.17, 86.86, 85.47 (d, $^3J_{PC}$ = 6.0 Hz), 84.91, 83.72 (d, $^3J_{PC}$ = 9.1 Hz), 74.39, 72.39, 70.26, 65.36 (d, $^2J_{PC}$ = 4.5 Hz), 37.92, 32.42 (d, $^3J_{PC}$ = 4.5 Hz), 11.51.

^{31}P NMR (162 MHz, D_2O) δ_P = 7.32 (d, $^2J_{PP}$ = 29.2 Hz, P_β), -11.95 (d, $^2J_{PP}$ = 29.2 Hz, P_α).
HRMS (ESI, negative ion). Calculated m/z for ($C_{20}H_{26}N_7O_{13}P_2S$) $[M + H]^-$: 666.0785, found 666.0717.

5′-deoxy-5′-selenothymidine 5′-pyrophosphate (P′ →5′) adenosine (2b) - dTSeppA

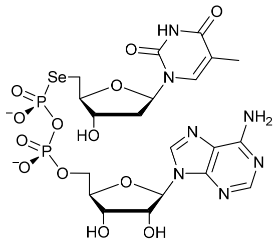

A suspension of 5′-deoxythymidinyl-5′-selenocyanate (Ref. 32 in the main text) (**6b**) (50.0 mg, 0.15 mmol) in 4:1 anhydrous chloroform:BSA (2.0 mL) under argon was sonicated for 20 minutes after which time a clear solution had formed and was left to stir at ambient temperature for a further 30 minutes. To this stirred solution was added a solution of $(TMSO)_3P$ (55.0 µL, 0.165 mmol, 1.1 eq.) in 4:1 anhydrous chloroform:BSA (0.65 mL) at room temperature and these conditions maintained for 18 hours. The reaction mixture was analysed by ^{31}P NMR. The reaction mixture was then transferred into a zirconia-lined vessel under argon and the residues from both the reaction flask and NMR tube were rinsed with anhydrous chloroform (2 x 0.1 mL). The vessel was stored at ambient temperature in a desiccator under vacuum for four hours until volatiles had been removed and the residue had the consistency of a paste. After equilibrating to atmospheric pressure, the jar was charged sequentially with AMP-morpholidate (**5**) (319 mg, 0.45 mmol, 3.0 eq.), tetrazole (22 mg, 0.315 mmol, 2.1 eq.), $MgCl_2•(H_2O)_6$ (46 mg, 0.225 mmol, 1.5 eq.), H_2O (32.0 µL, 1.80 mmol, 12 eq.) and a 15 mm zirconia ball. The vessel was sealed and vibrated at 30 Hz for 90 minutes and allowed to cool to room temperature. The crude reaction mixture was extracted from the vessel using both physical fracturing of the solid residues (with polypropylene automatic pipette tip) and successive rinsing with H_2O (3 x 1 mL) and with methanol (1 mL) into eppendorf tubes. The combined suspensions were sonicated, filtered in 1 mL aliquots through a Spin-X cellulose acetate centrifuge filter (0.45 µm) at 12,000 rpm and the solids rinsed with H_2O (0.5 mL). The combined extracts were immediately analysed by ^{31}P NMR. The reaction mixture was purified by C18 RP-HPLC using IP buffers (gradient G2) from which a single peak corresponding to pure dTSeppA (**7**) was collected. Combined pure fractions were concentrated *in vacuo* and subject to desalting following HPLC isolation using TEAB buffers (gradient G3) and repeated coevaporation with deionised water. Isolated yield of dTSeppA•~1.8 (Et_3NH) (**2b**): 1714 OD^{260nm} units (0.073 mmol, 49%). t_R (G1) = 14.2 min.

^1H NMR (600 MHz, D_2O) δ_H = 8.41 (1H, s, H2), 8.06 (1H, s, H8), 7.19 (1H, s, H6-dT), 6.01 (1H, ψt, $^3J_{HH}$ = 6.9 Hz, H1′-dT), 5.98 (1H, d, $^3J_{HH}$ = 5.6 Hz, H1′- rA), 4.64 (1H, t, $^3J_{HH}$ = 5.4 Hz, H2′-rA), 4.45 (1H, dd, $^3J_{HH}$ = 5.1 Hz, 4.0Hz, H3′-rA), 4.37-4.34 (1H, m, H3′-dT), 4.32-4.30 (1H, m, H4′- rA), 4.22-4.16 (2H, m, H5′,H5″-rA), 4.11-4.08 (1H, m, H4′-dT), 3.04-2.99 (2H, m, H5′,H5″-dT), 2.23-2.17 and 2.15-2.09 (2H, 2 x m, H2′,H2″-dT), 1.67 (3H, s, CH_3).
^{13}C NMR (101 MHz, D_2O) δ_C = 165.97, 155.14, 152.46, 151.18, 148.80, 139.92, 136.83, 118.29, 111.20,

86.89, 85.77 (d, ${}^3J_{PC}$ = 4.5 Hz), 84.93, 83.73 (d, ${}^3J_{PC}$ = 9.1 Hz), 74.40, 72.99, 70.28, 65.34 (d, ${}^2J_{PC}$ = 6.0 Hz), 37.88, 26.46 (d, ${}^3J_{PC}$ = 4.5 Hz), 11.51.

^{31}P NMR (243 MHz, D_2O) δ_P = -3.32 (d, ${}^2J_{PP}$ = 31.6 Hz; ${}^1J_{PSe}$ = 409 Hz P_β), -12.09 (d, ${}^2J_{PP}$ = 31.6 Hz, P_α).

^{77}Se NMR (114 MHz, D_2O, external 0.25M KSeCN standard in D_2O: -329 ppm) δ_{Se} = 141.22 (d, ${}^1J_{PSe}$ = 415 Hz).

HRMS (ESI, negative ion). Calculated m/z for ($C_{20}H_{26}N_7O_{13}P_2Se$) $[M + H]^-$: 714.0229, found 714.0195 – mass error 4.8 ppm.

5′-deoxy-5′-(5-nitropyridyl-2-disulfanyl)thymidine (6a) –NPySSdT

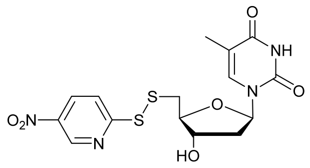

To a mixture of 5′-deoxy-5′-(4-methoxybenzylthio)thymidine (378 mg, 1.0 mmol) and 2,2′-dithiobis(5-nitropyridine) (403 mg, 1.3 mmol, 1.3 eq.) was added trifluoroacetic acid / thioanisole (39/1 – 10.2 mL) and the solution stirred under argon at room temperature for two hours. The reaction mixture was concentrated under vacuum, diluted with dichloromethane and purified by silica gel chromatography eluting with 5 - 10% methanol in dichloromethane. Appropriate fractions were reduced *in vacuo* to yield pure **S1** as a pale yellow solid (326 mg, 0.79 mmol, 79%).

^{1}H NMR (400 MHz, D_6-DMSO) δ_H = 11.30 (1H, s, -NH), 9.23 (1H, s, ArH), 8.57 (1H, d, ${}^3J_{HH}$ = 8.6 Hz, ArH), 8.08 (1H, d, ${}^3J_{HH}$ = 9.0 Hz, ArH), 7.47 (1H, s, H6), 6.15 (1H, ψt, ${}^3J_{HH}$ = 7.0 Hz, H1′), 5.42 (1H, s, -OH) 4.20 (1H, s, H4′), 3.89 (1H, s, H3′), 3.30-3.15 (2H, m, H5′, H5″), 2.34-2.20 (1H, m, H2′), 2.11-1.99 (1H, m, H2″), 1.77 (3H, s, $-CH_3$).

^{13}C NMR (101 MHz, D_6-DMSO) δ_C = 167.32, 163.62, 150.43, 144.75, 142.21, 136.23, 132.51, 119.62, 109.77, 84.06, 84.01, 72.49, 41.31, 37.74, 12.07.

HRMS (ESI, positive ion). Calculated m/z for $C_{15}H_{16}N_4O_6S_2K$ $[M+K]^+$: 451.0148; found 451.0160 – mass error 2.7 ppm.

pH titrations and pKa determination of pyrophosphorochalcogenate-linked dinucleotides

Pyrophosphorochalcogenate-linked dinucleotides (dASppA (**1a**) (Ref. 14 in the main text), dASeppA (**1b**), dTSppA (**2a**), dTSeppA (**2b**) and) where diluted to a final volume of 700μL (10 mM) in 10% D_2O solution. The solution was adjusted to pH ~10 with 0.2M NaOH and then titrated with 2.5 – 10μL HCl (0.2M). After each addition, the sample was thoroughly mixed, the pH measure using a Hach H160 portable pH meter equipped with a PH47-SS probe, and the ^{31}P NMR (202 MHz) recorded. The NMR spectra were processed using Topspin software and the chemical shifts automatically peak picked by Topspin. Chemical shift data was plotted against pH, and a best fit was obtained using the curve fit subroutine of the python scipy package.

dT(Ch)MP degradation studies

A suspension of 5′-deoxy-5′-(5-nitropyridyl-2-disulfanyl)thymidine) (**6a**) (185 mg, 0.45 mmol)5′-deoxythymidinyl-5′-selenocyanate[3] (**6b**) (149 mg, 0.45 mmol) or in 4:1 anhydrous chloroform:BSA (6.0 mL) was sonicated for 20 minutes and left to stir at ambient temperature under argon for 30 minutes. To this stirred solution was added a solution of $(TMSO)_3P$ (165 µL, 0.495 mmol, 1.1 equiv) in 4:1 anhydrous chloroform:BSA (1.95 mL) and these conditions maintained for one hour. ^{31}P NMR was performed and showed completed reaction. The MA stock reaction mixture was quickly dispensed in 0.5 mL aliquots (each containing 0.0283 mmol of putative persilylated product) into a small glass vial located in argon saturated desiccator and left overnight at ambient temperature under vacuum protected from light.

The residual dried MA-RM aliquots were redissolved using degassed 1M buffer (pH 3, 7 or 11) (see below) (0.53 mL) following sonication for 30 seconds to give a final concentration of 53 mM. The insoluble material (symmetrical nucleoside disulfide (5′,5′)-dTSSdT or diselenide (5′,5′)-dTSeSedT respectively) was filtered off using 45 µm PTFE syringe filter and the degradation was monitored hourly using ^{31}P NMR (242 MHz) at appropriate temperature (298K at variable pH and 298K/308K/318K at pH 7).

Following buffers were used for the nucleoside monophosphorothiolate and monophosphoroselenolate degradation studies:

pH 3.0	1M Gly•HCl 100 mM NaCl
pH 7.0	1M HEPES•NaOH 100 mM NaCl
pH 11.0	1M CAPS•NaOH 100 mM NaCl

Raw analytical data

M-A reaction of NCSedA (5)
^{31}P NMR 162 MHz
D_2O (external lock)

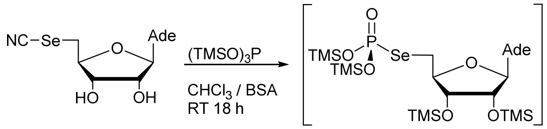

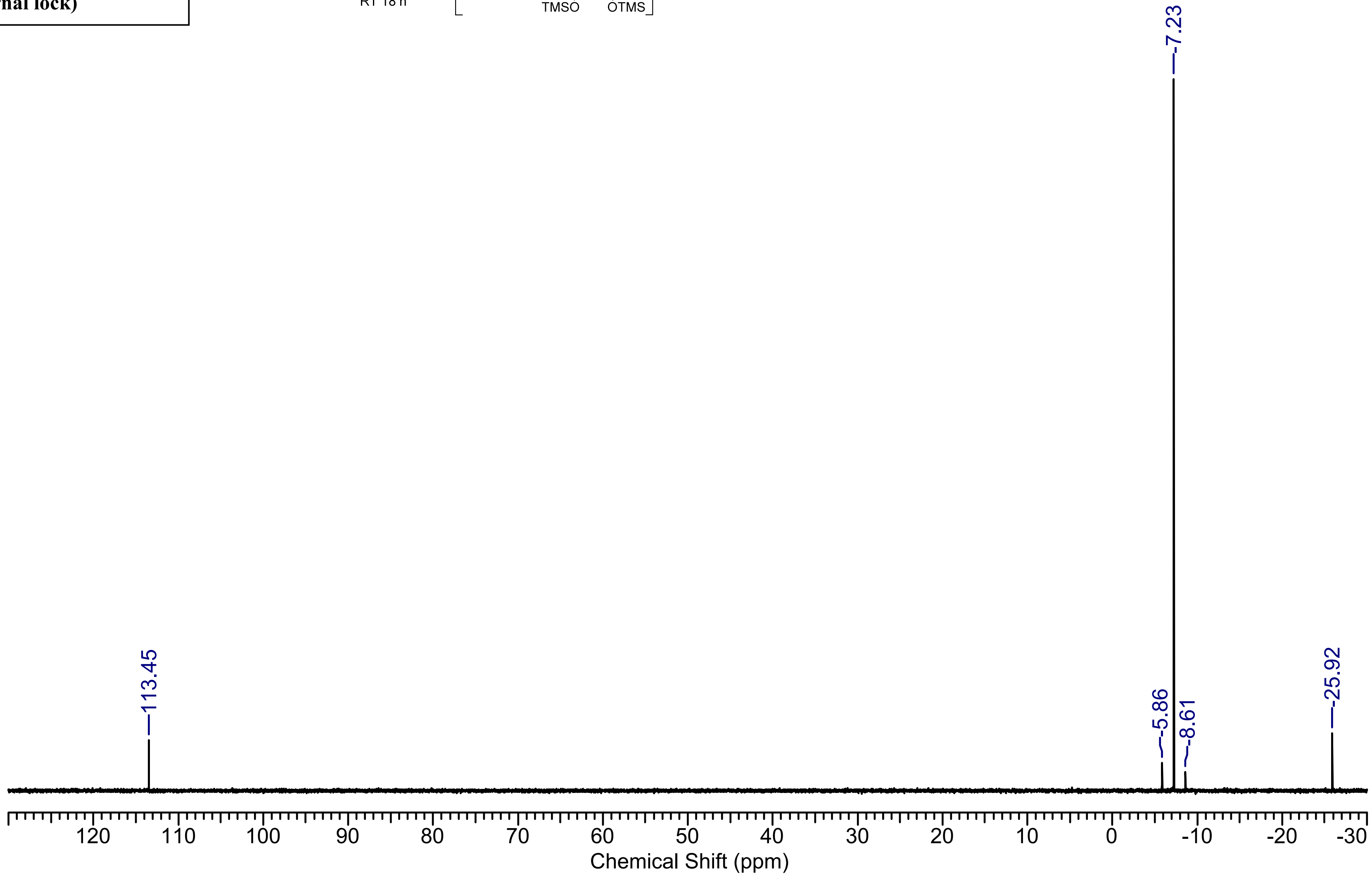

Crude phosphate coupling reaction (dASeppA: 1b)
^{31}P NMR 243 MHz
D_2O (external lock)

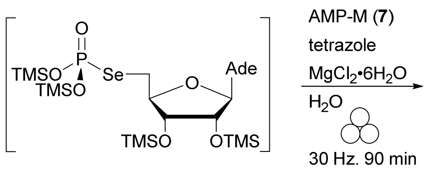

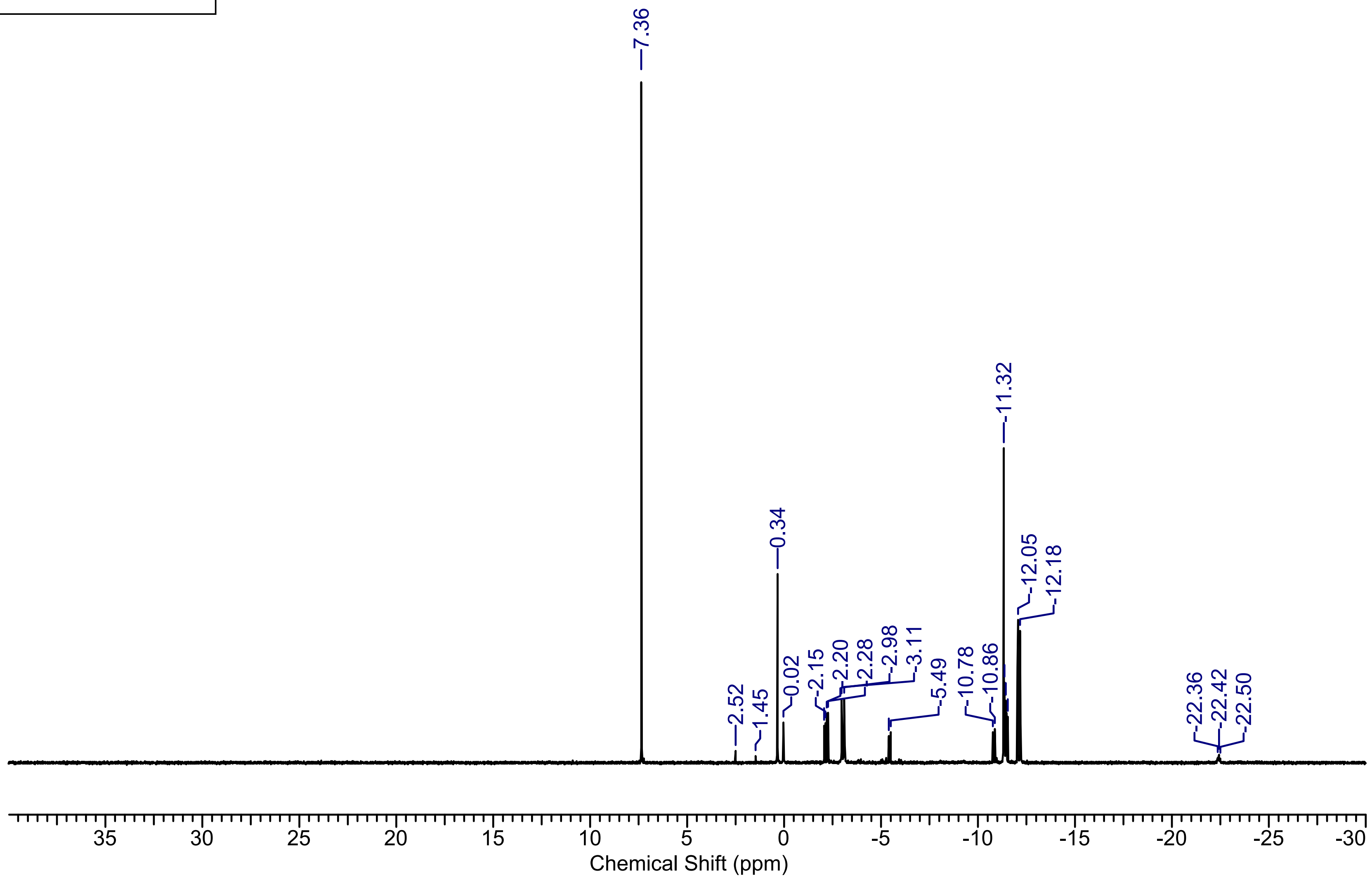

Analytical C18 RP-HPLC of crude phosphate coupling reaction mixture (dASeppA) - gradient G1

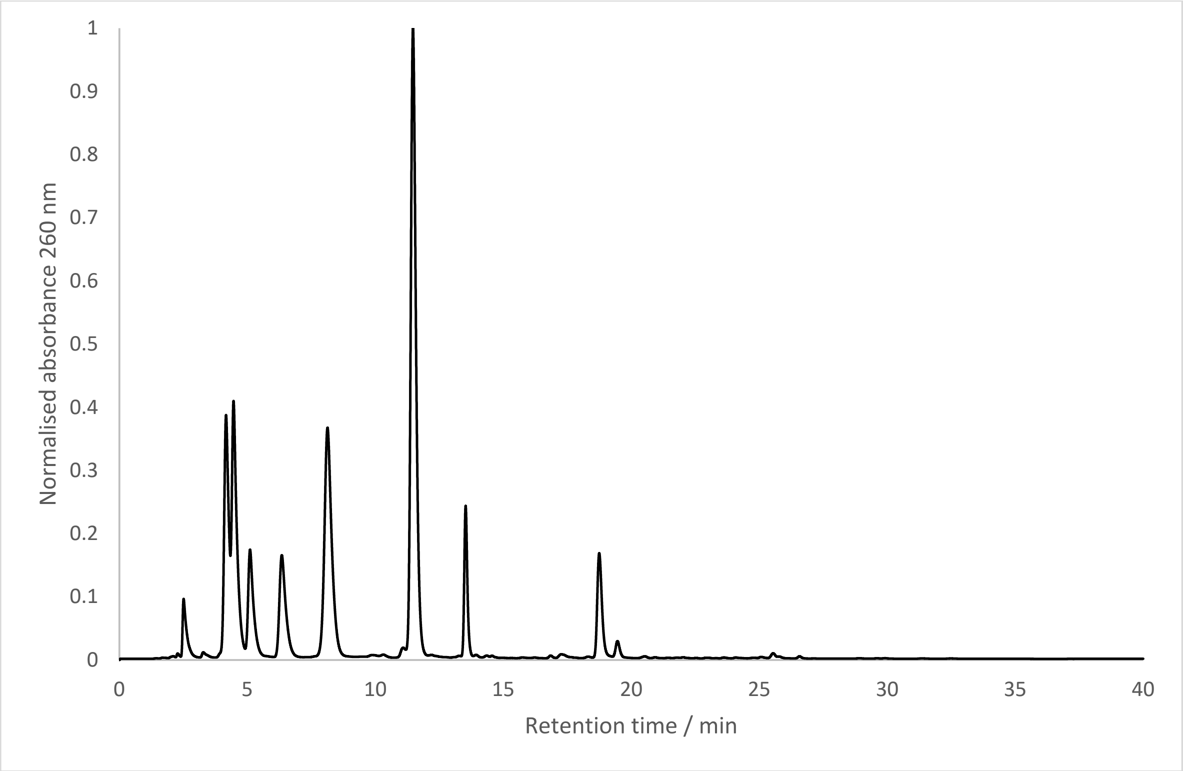

Analytical C18 RP-HPLC of pure dASeppA (1b) – gradient G1

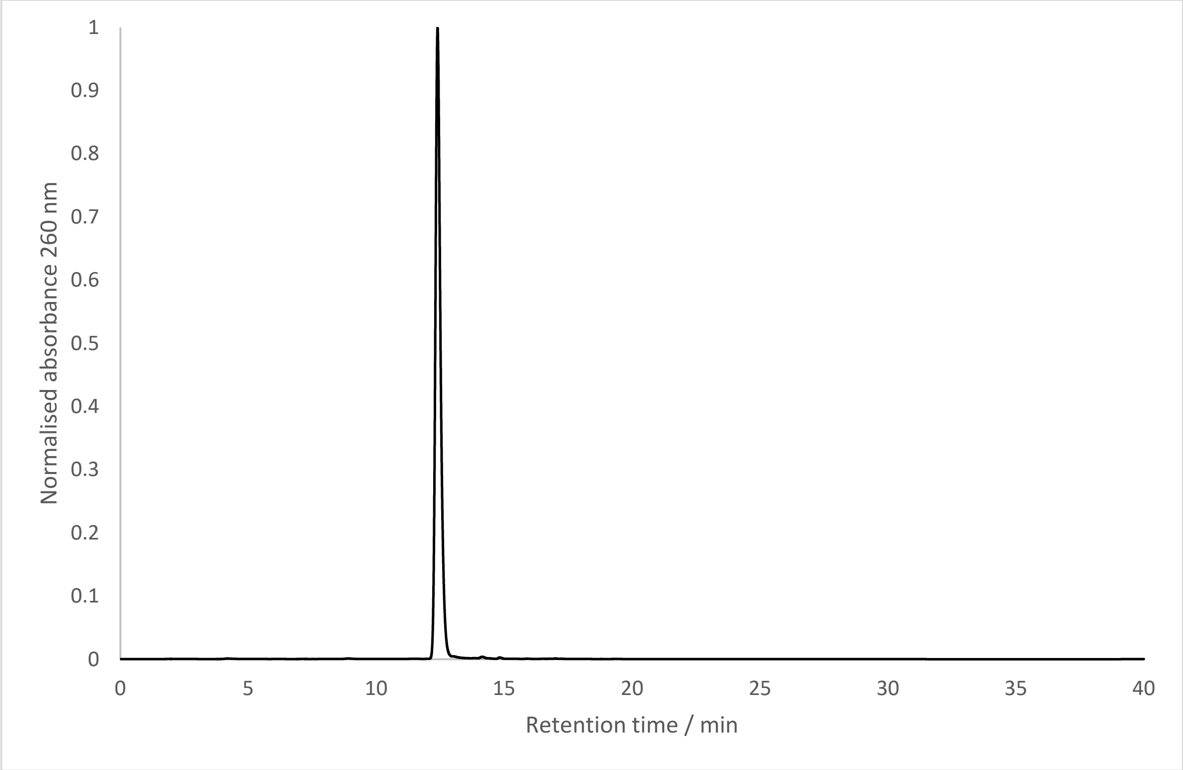

dASeppA – 1b

^{1}H NMR 600 MHz

D_2O

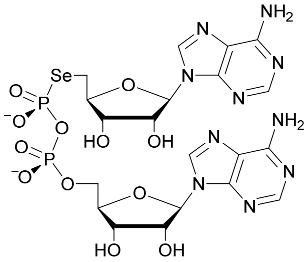

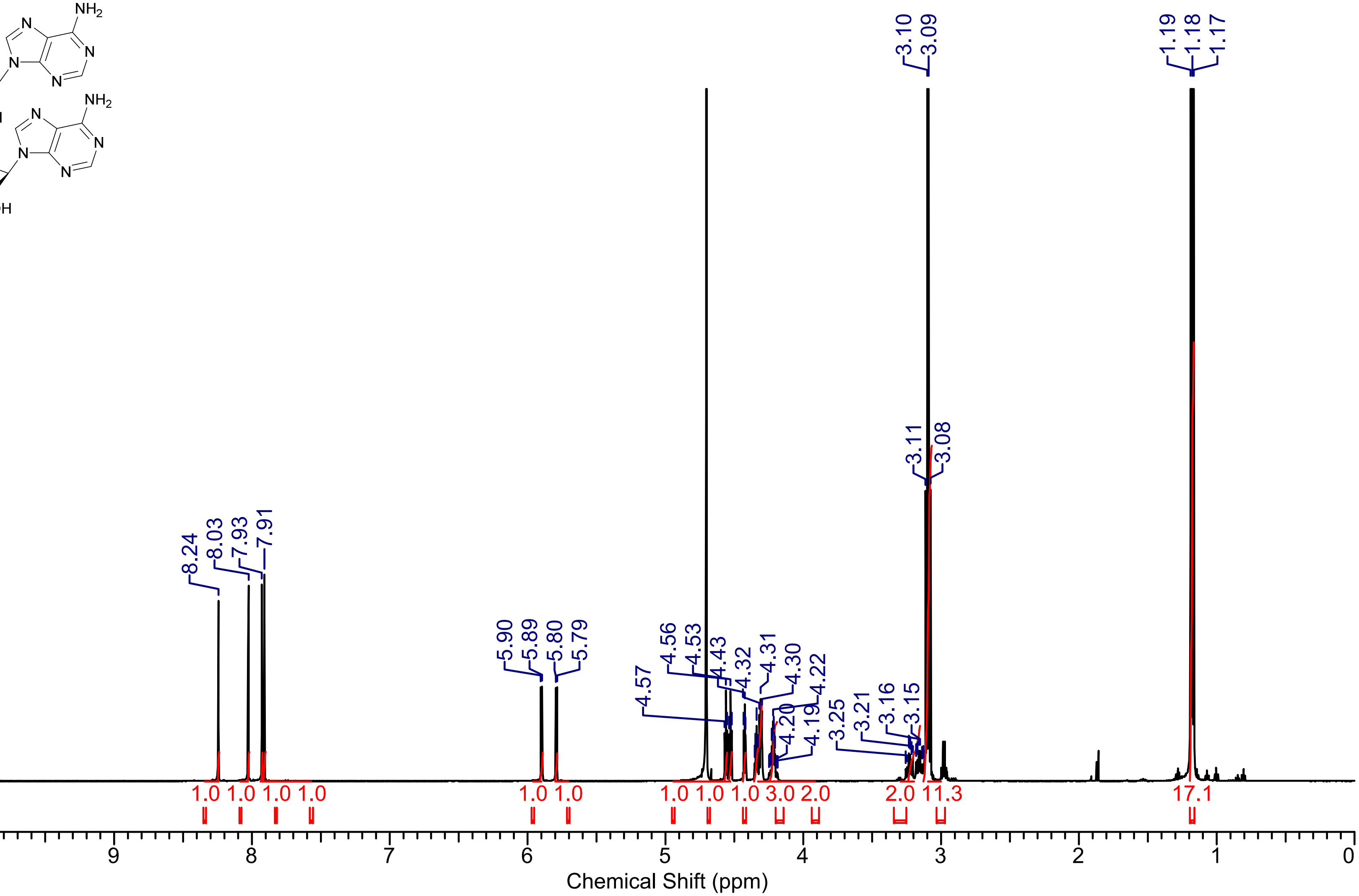

dASeppA – 1b

^{1}H –^{1}H COSY 600 MHz

D_2O

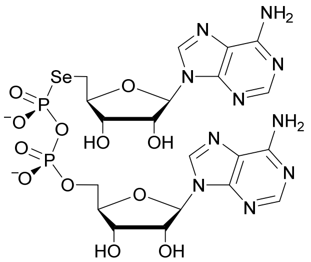

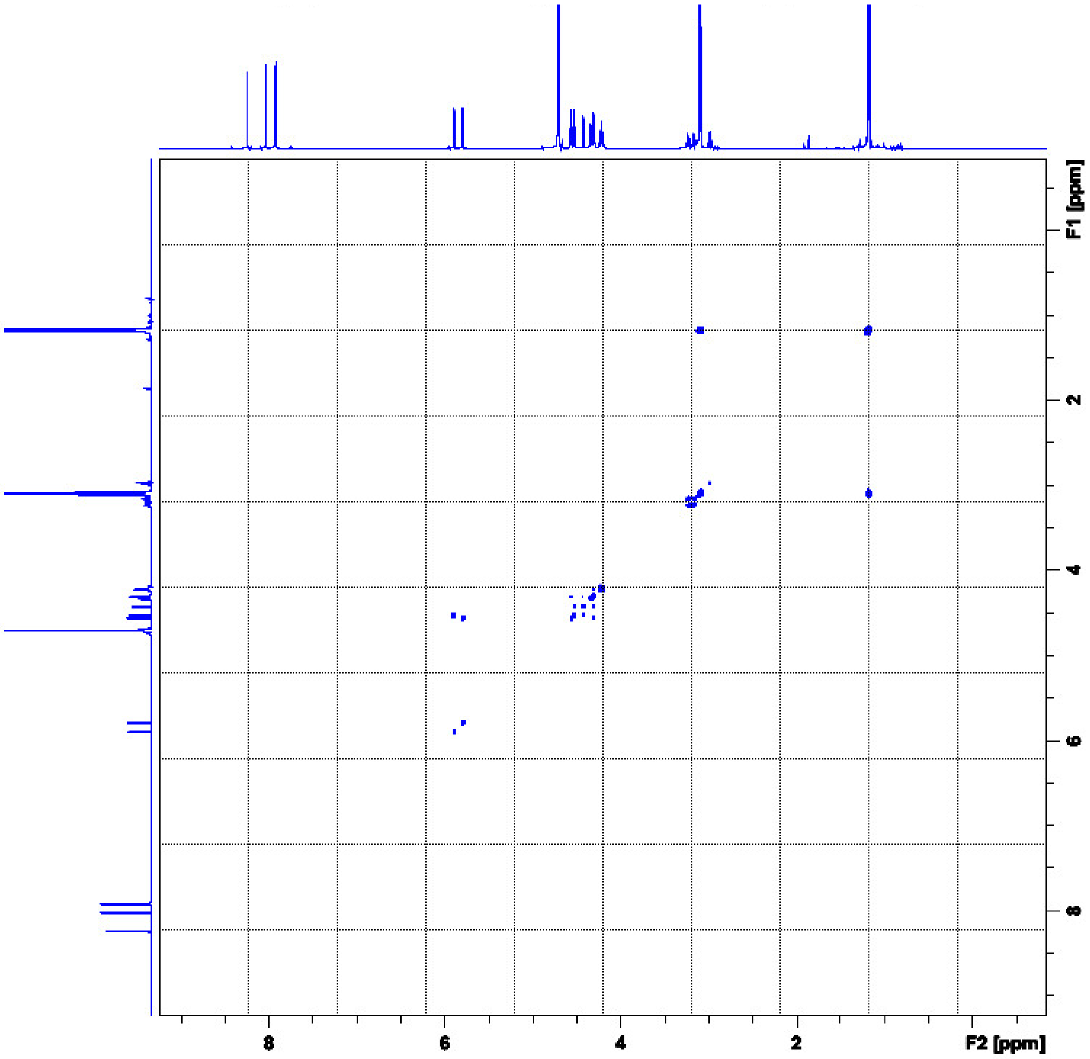

dASeppA – 1b

^{1}H-^{1}H COSY 600 MHz D$_2$O (7.0 – 2.0 ppm expansion)

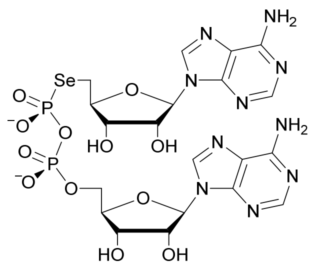

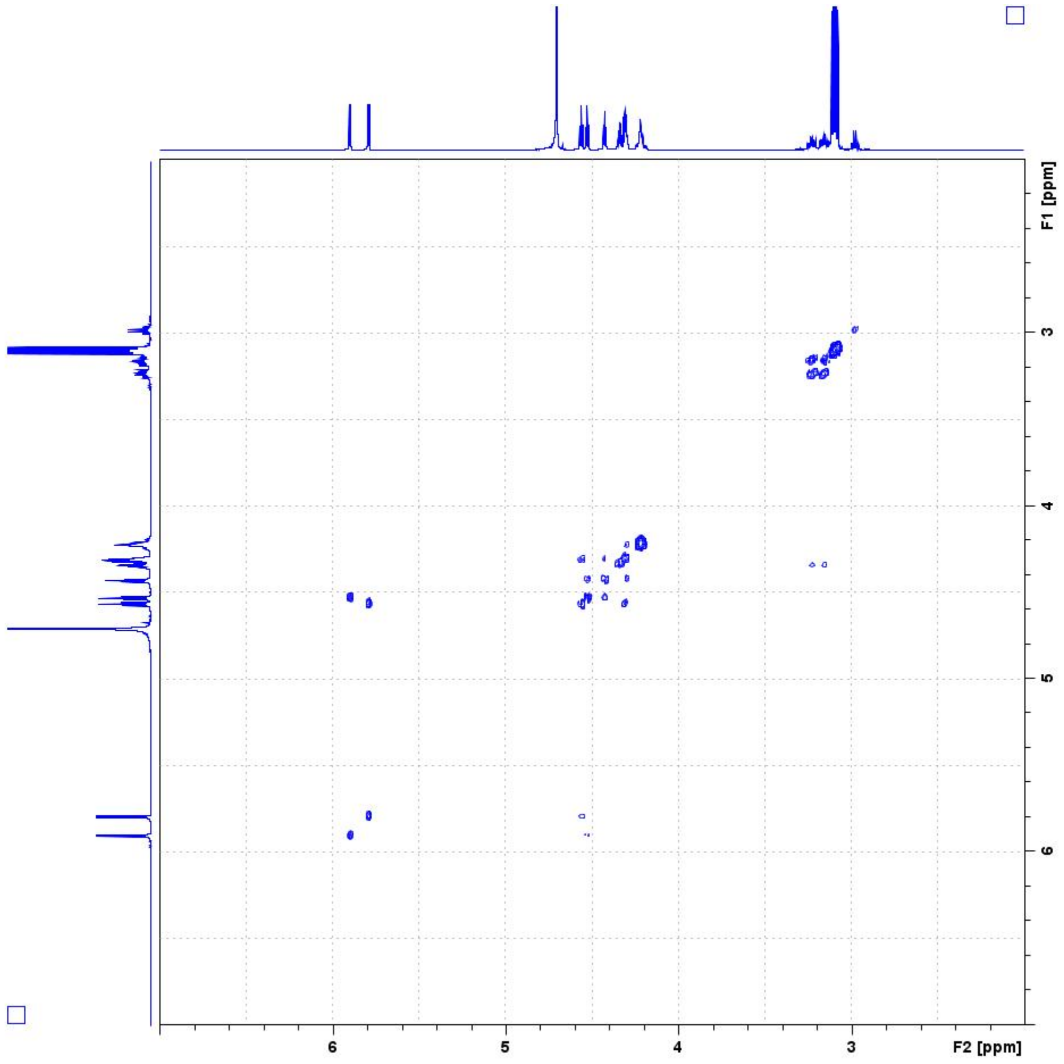

dASeppA – 1b

^{13}C NMR 151 MHz

D_2O

(expansions showing P-C couplings and overlapping resonances)

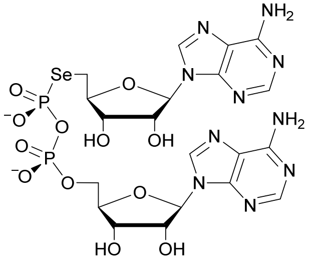

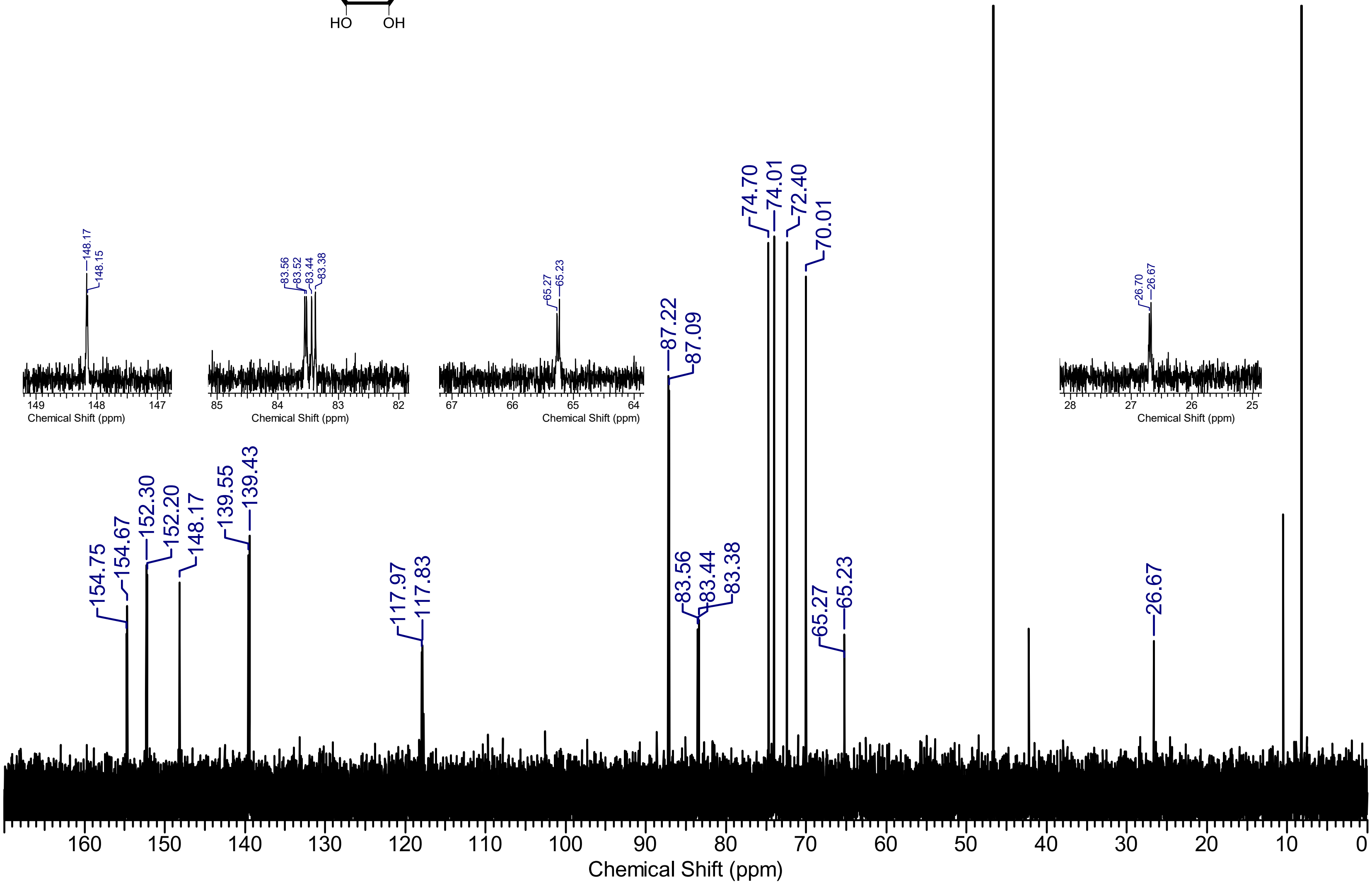

dASeppA – 1b

^{13}C NMR DEPT135

151 MHz

D_2O

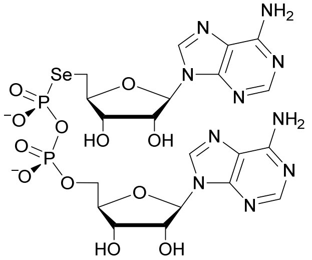

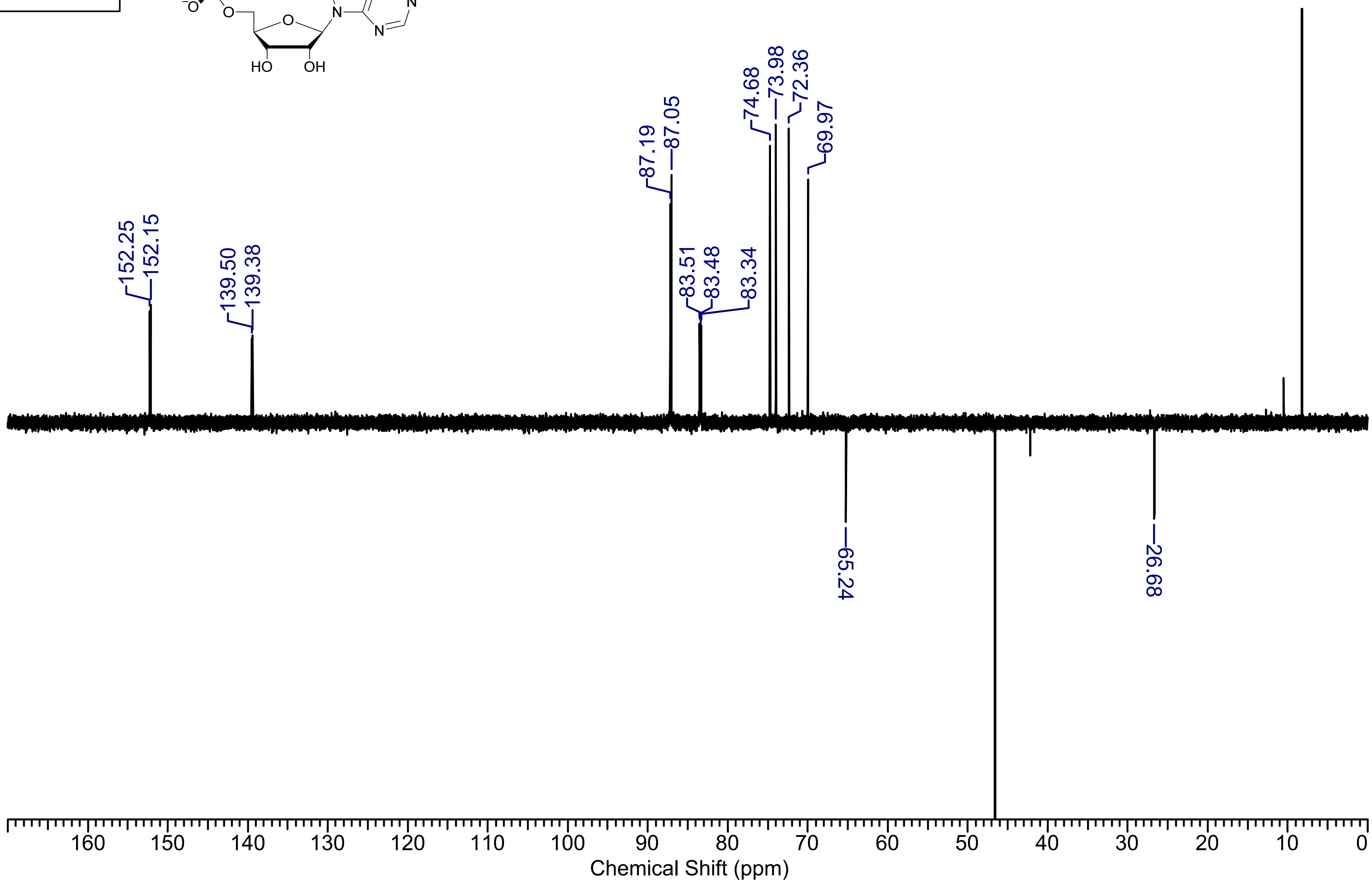

dASeppA – 1b

^{13}C-^{1}H HSQC 600 MHz

D_2O

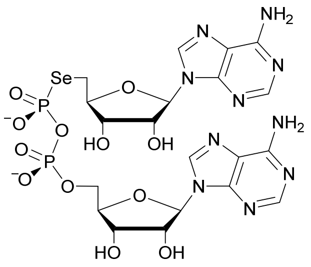

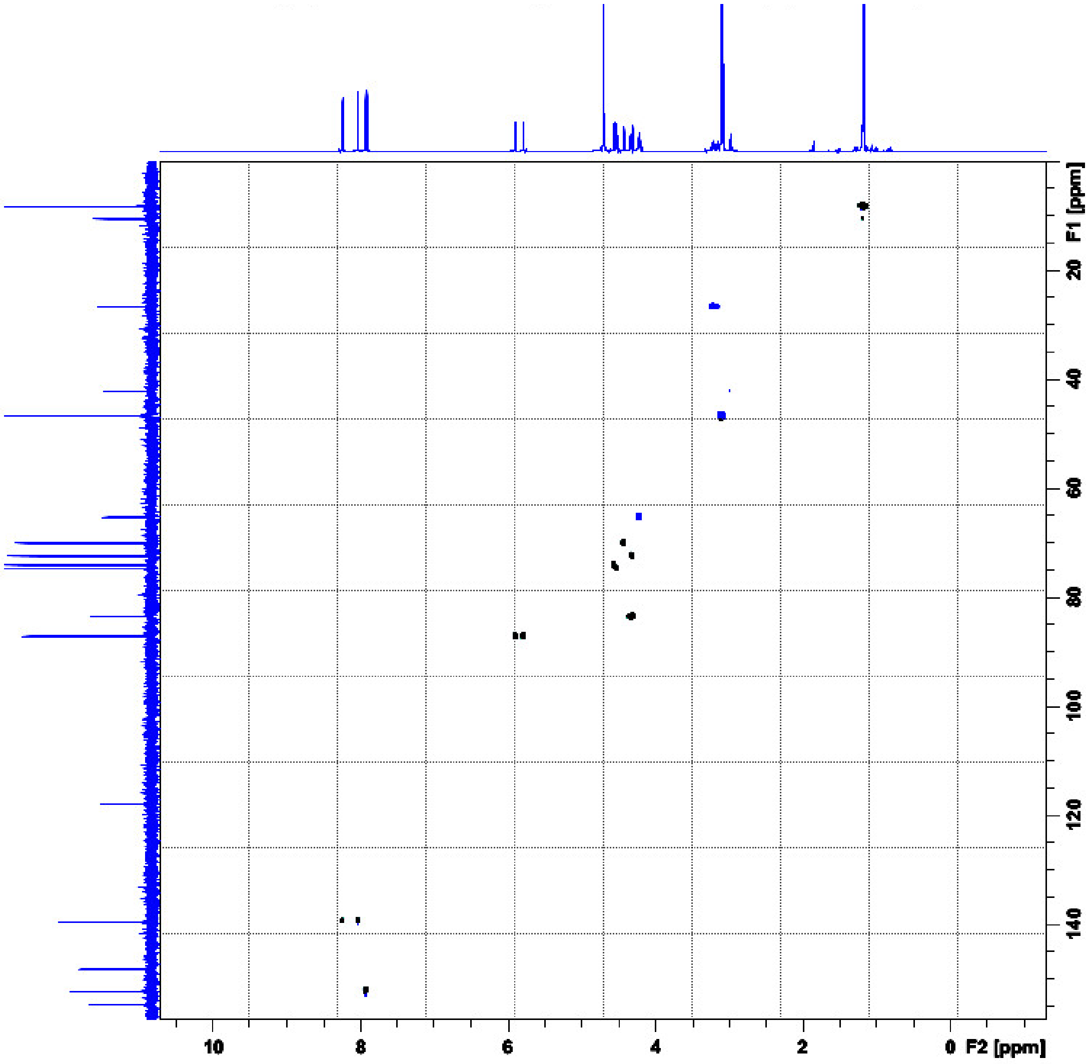

dASeppA – 1b

^{31}P NMR 243 MHz

D_2O

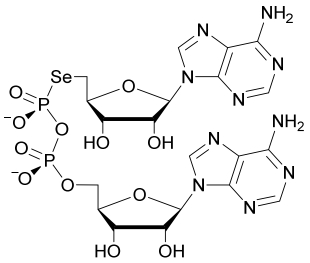

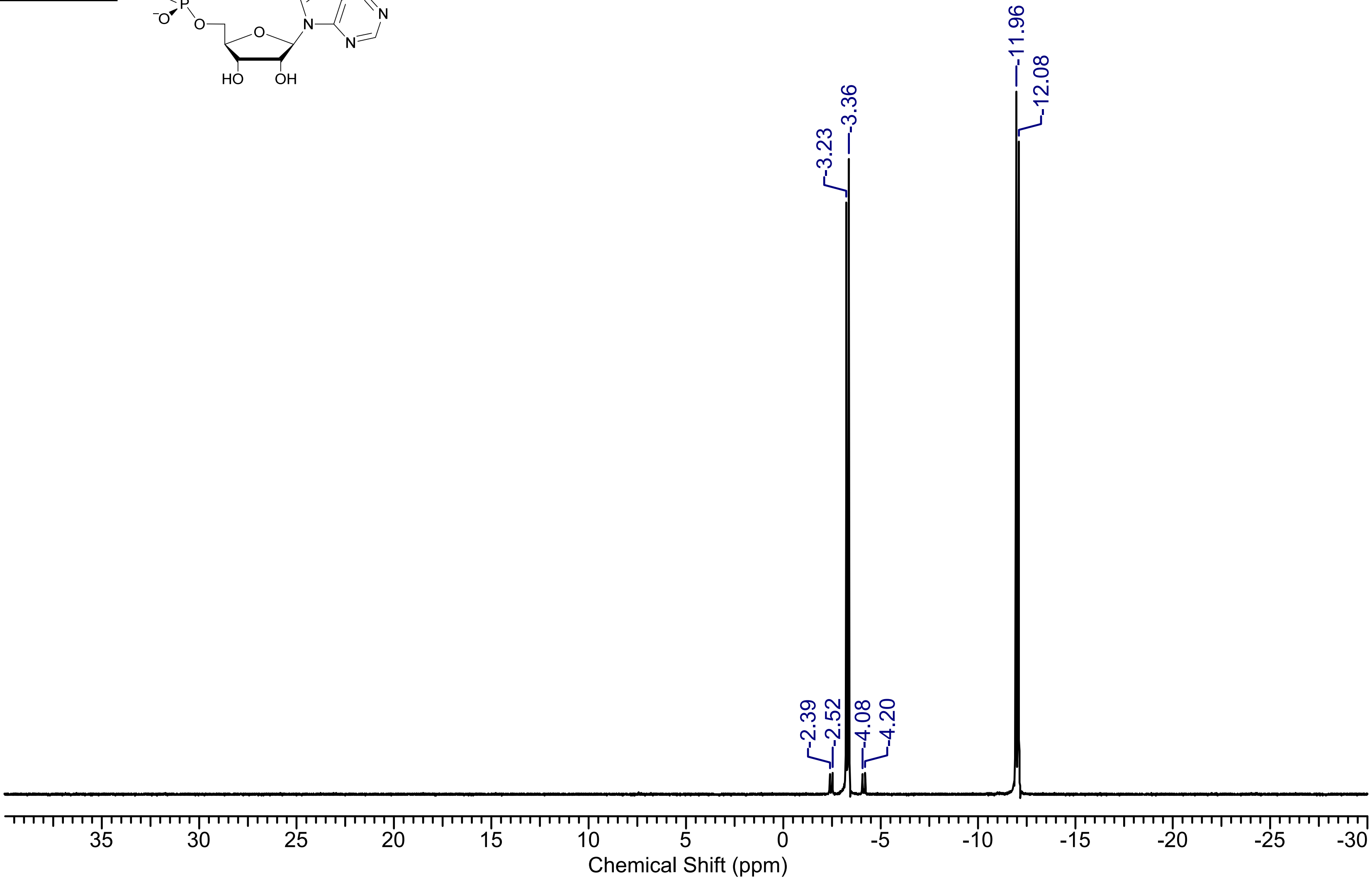

dASeppA – 1b

(+0.25M KSeCN in D_2O)

^{77}Se NMR 114 MHz

D_2O

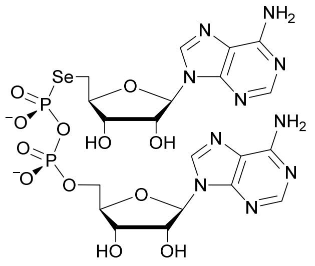

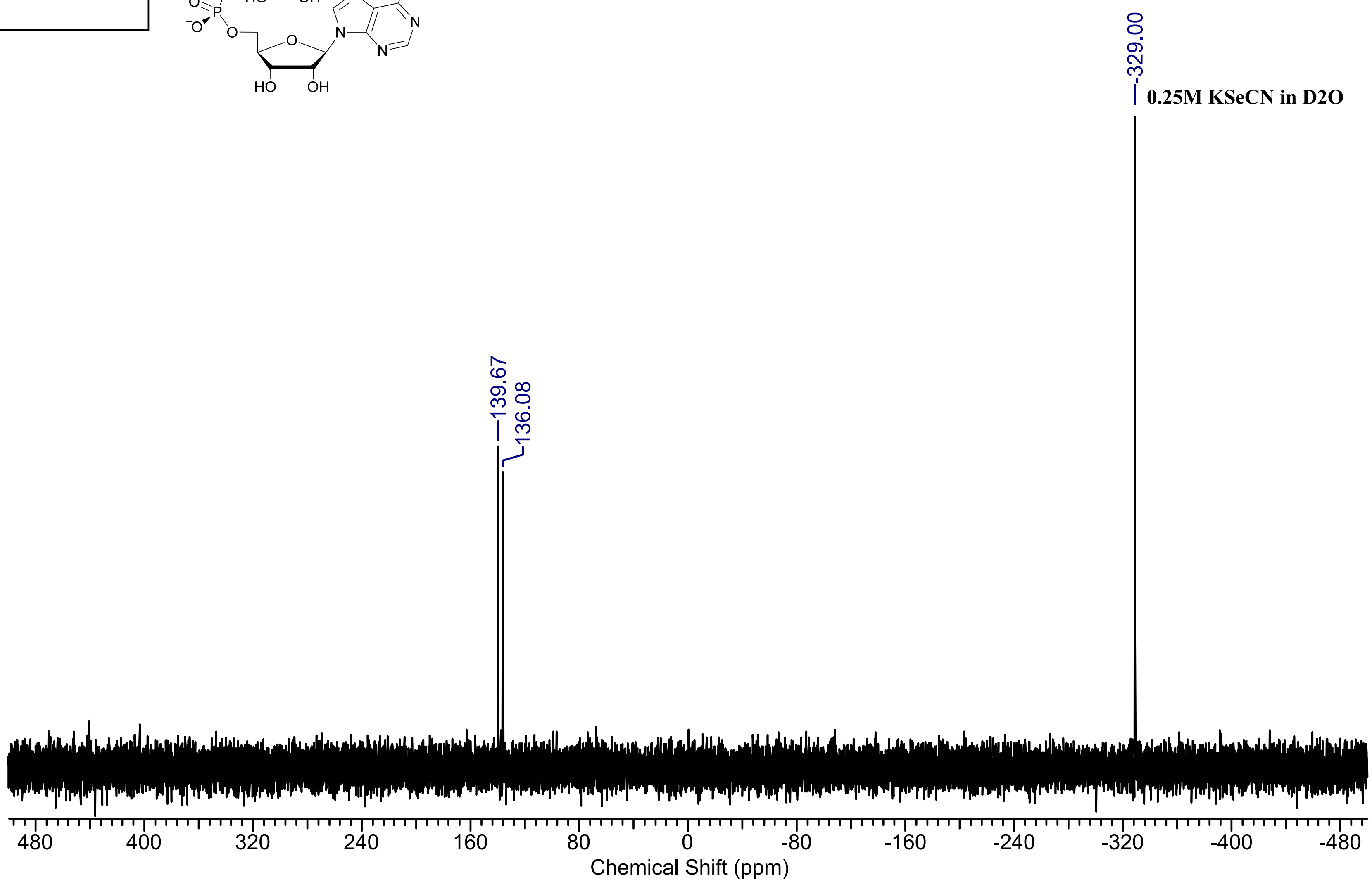

dASeppA – 1b

HRMS (ESI, negative ion)

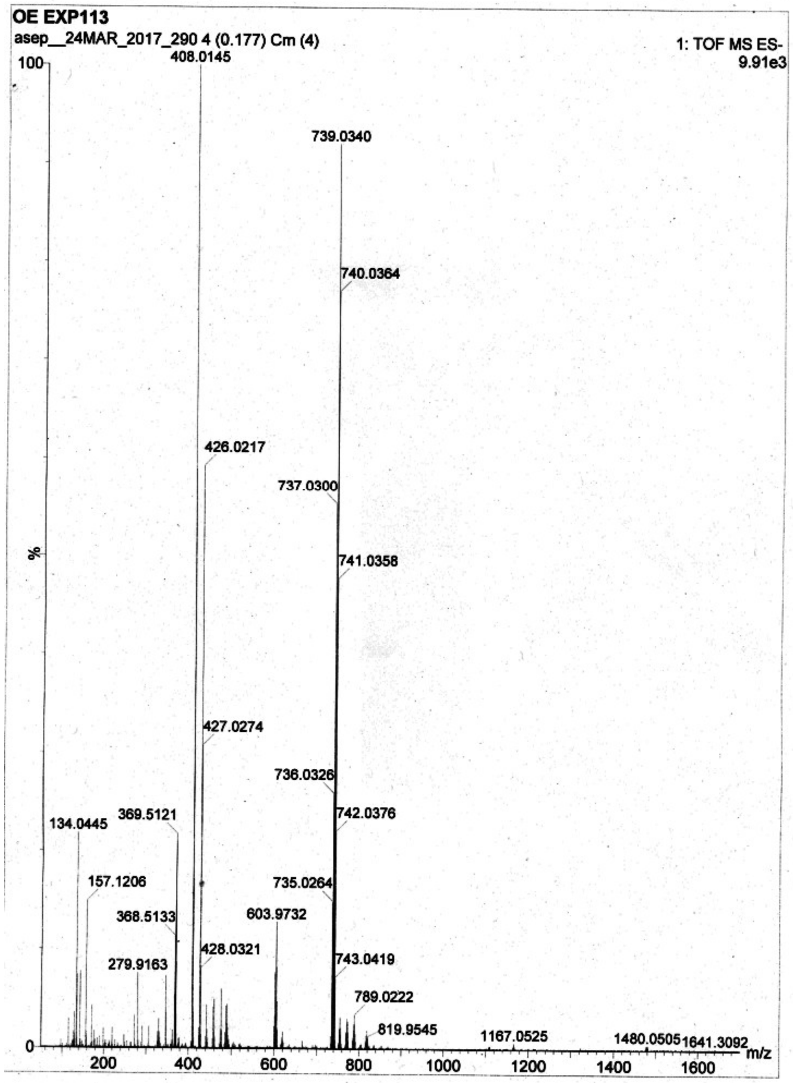

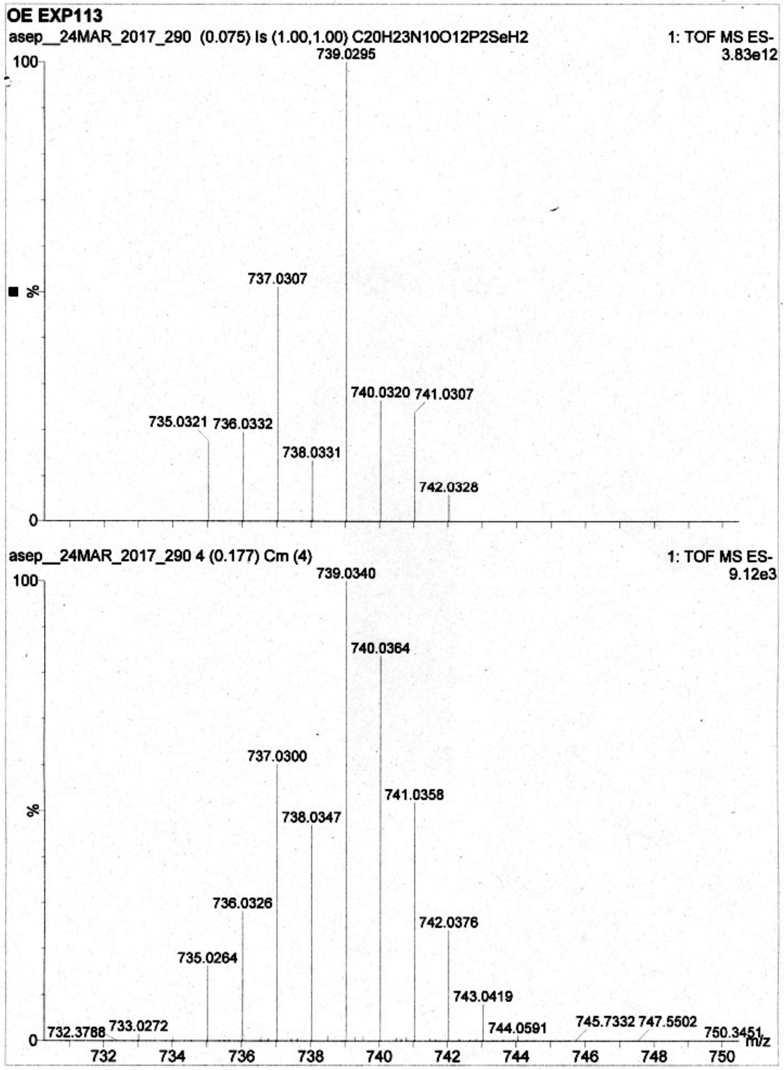

M-A reaction of NPySSdT (6a)

^{31}P NMR 162 MHz

D_2O (external lock)

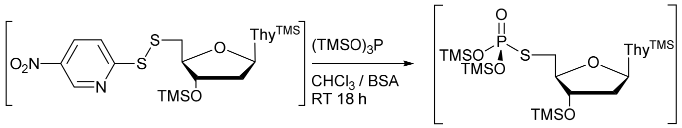

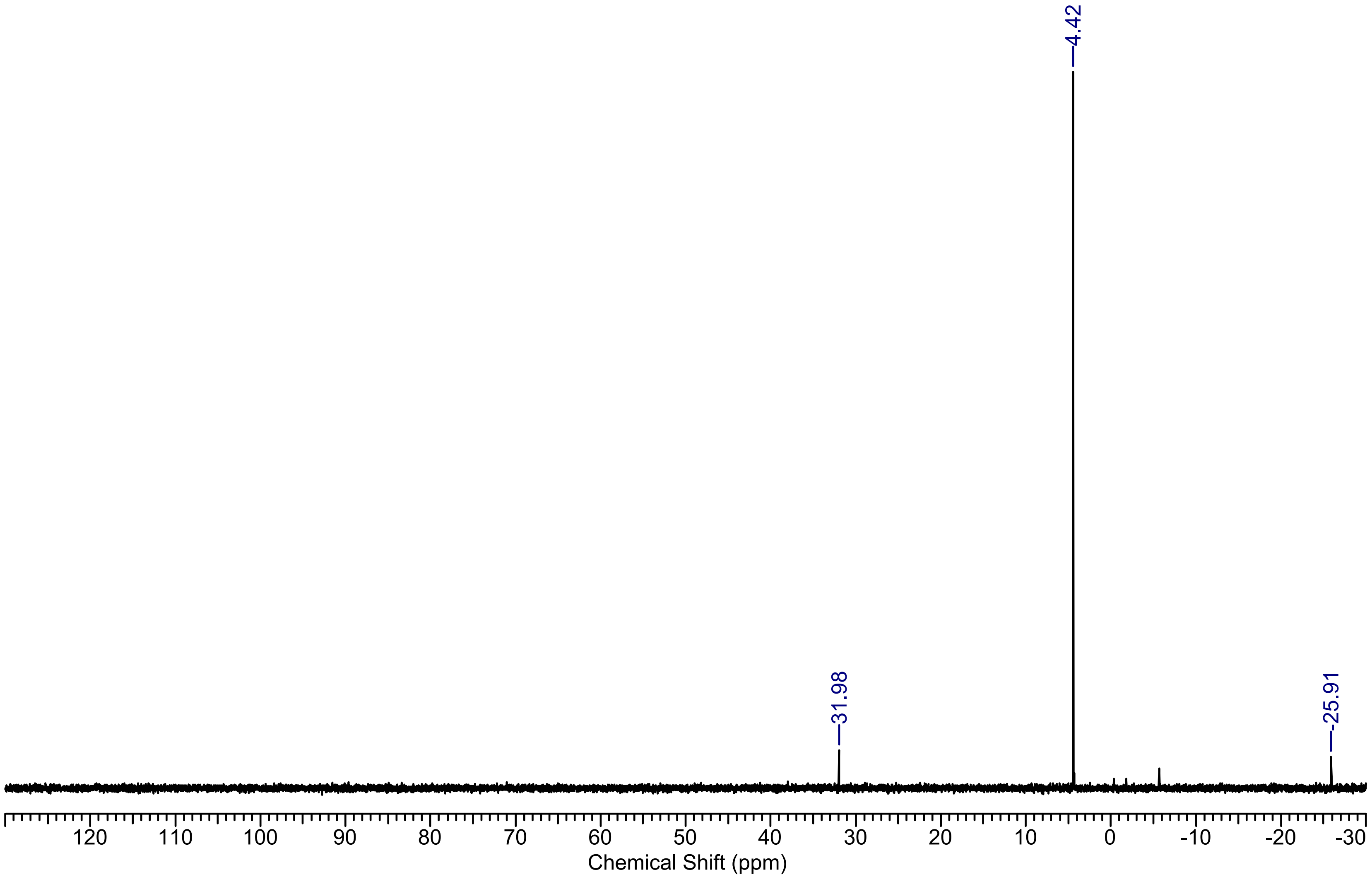

Crude phosphate coupling reaction (dTSppA: 2a)
^{31}P NMR 162 MHz
D_2O (external lock)

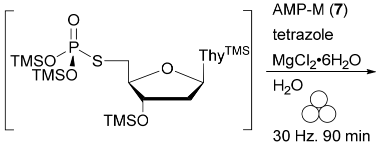

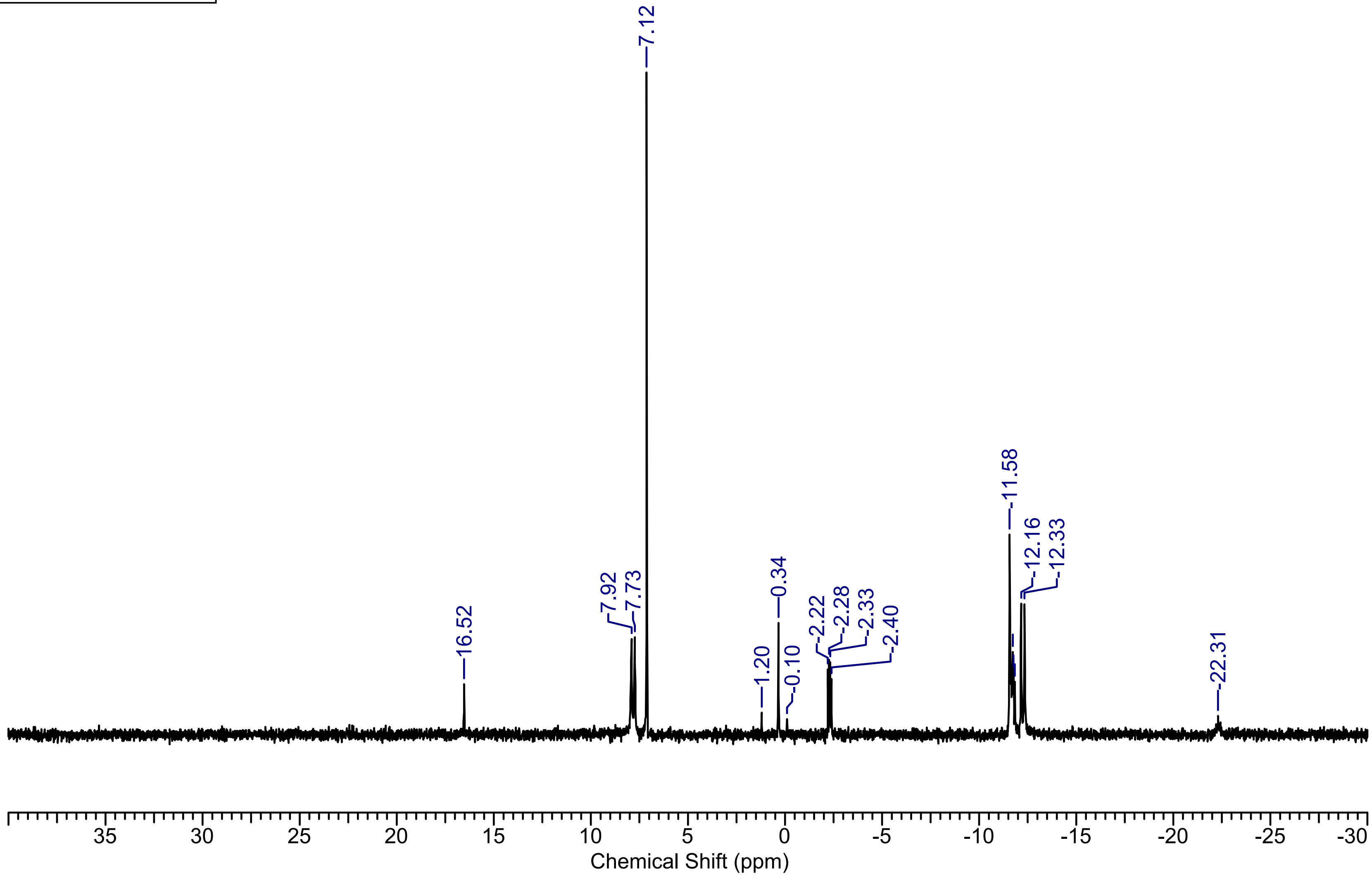

Analytical C18 RP-HPLC of crude dTSppA reaction mixture - gradient G1

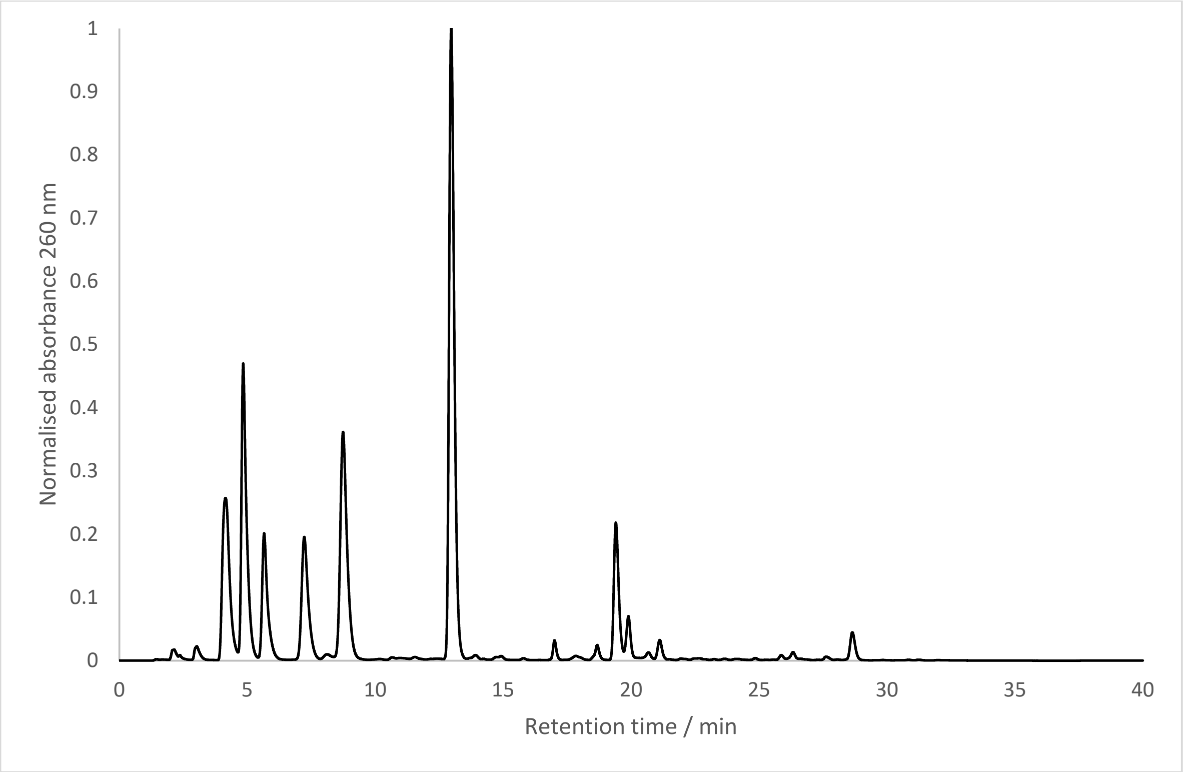

Analytical C18 RP-HPLC of pure dTSppA (2a) - gradient G1

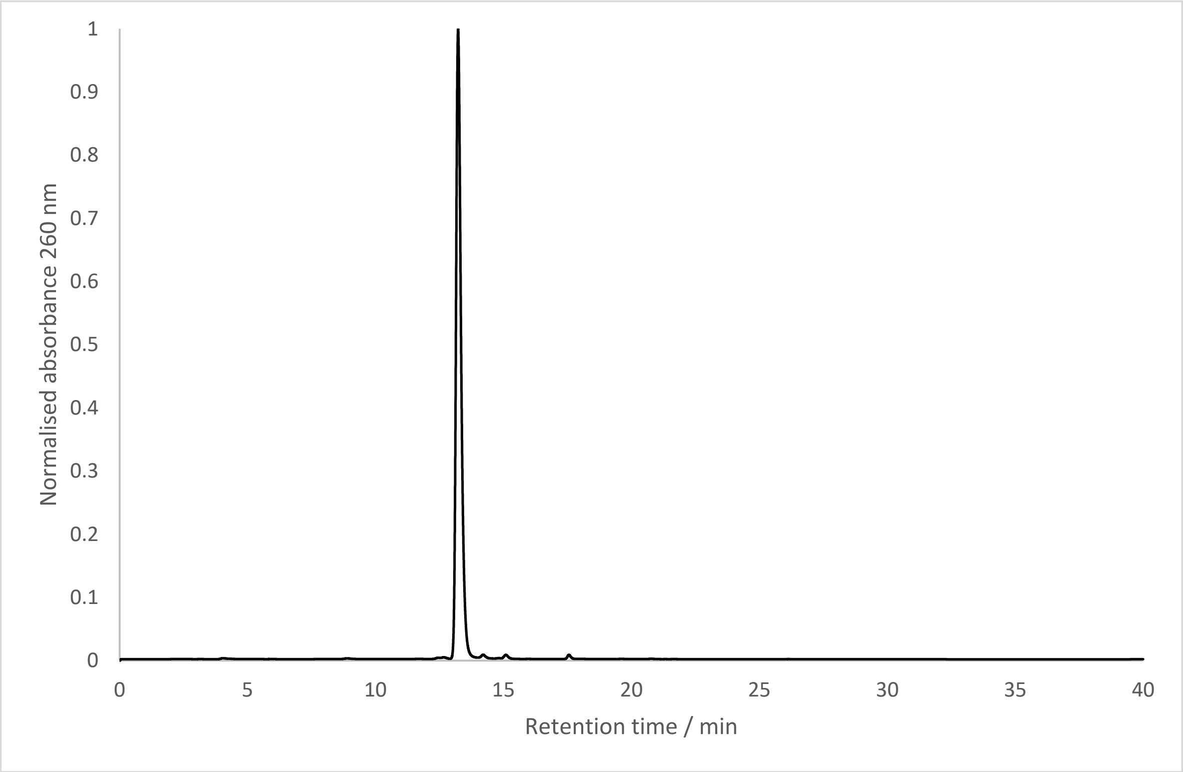

dTSppA – 2a

^{1}H NMR 400 MHz

D_2O

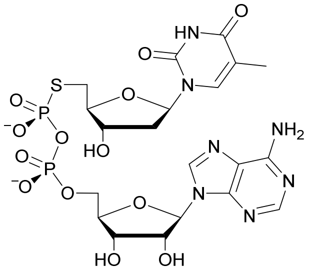

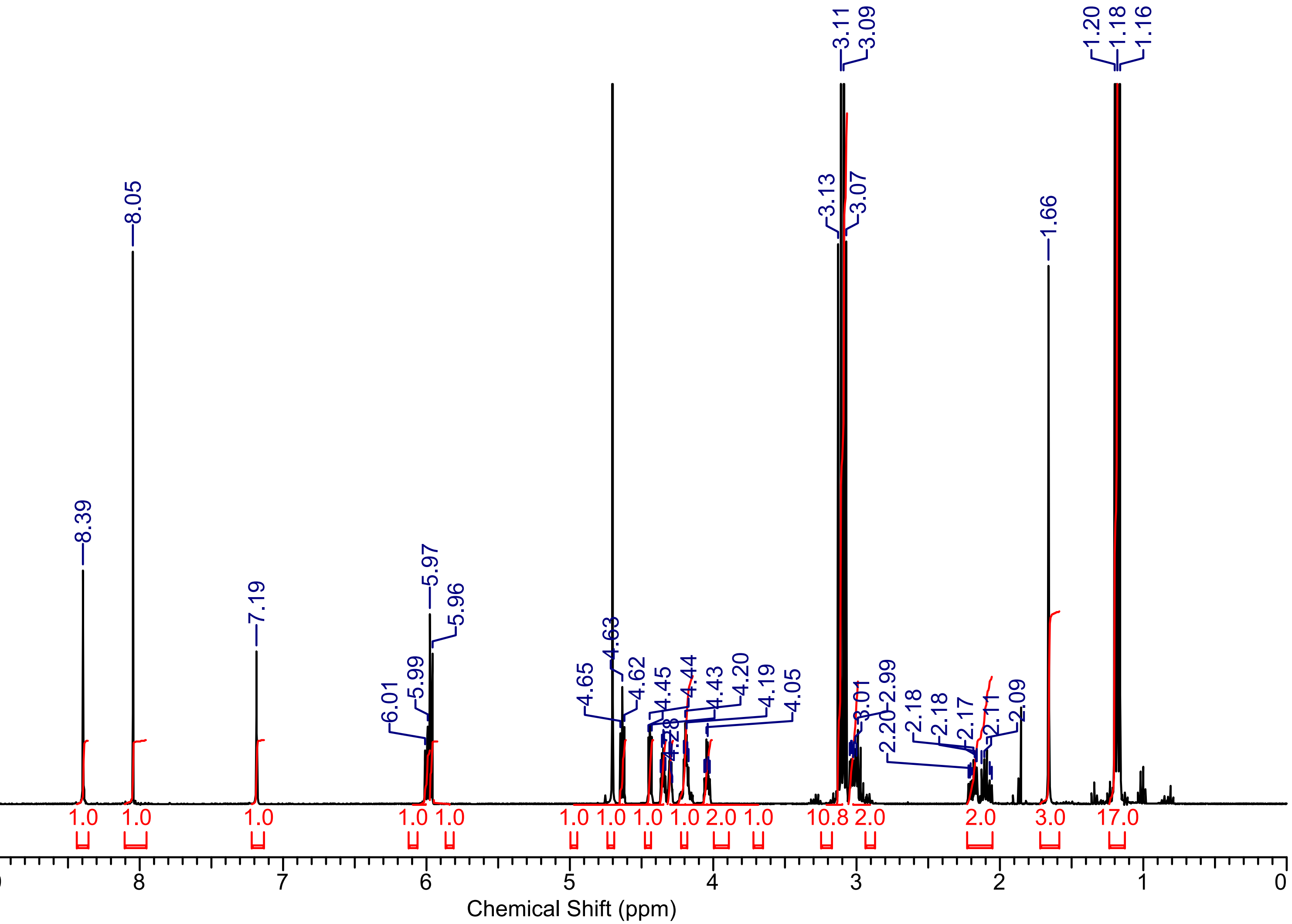

dTSppA – 2a

^{1}H-^{1}H COSY 400 MHz

D_2O

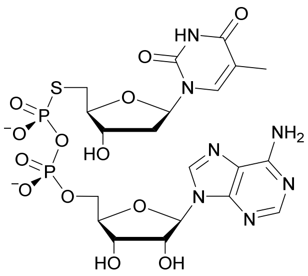

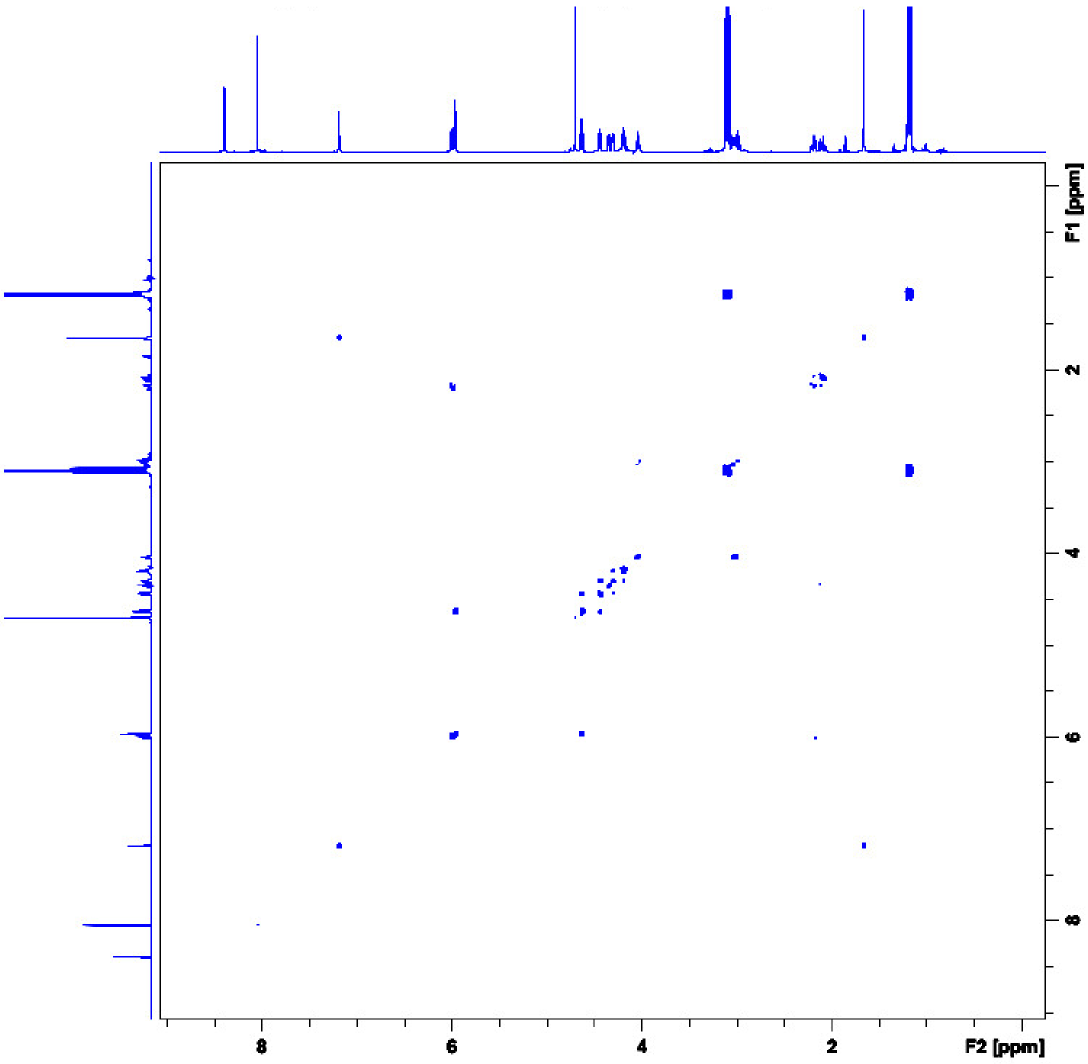

dTSppA – 2b

^{13}C NMR 151 MHz

(with expansions to show P-C couplings)

D$_2$O

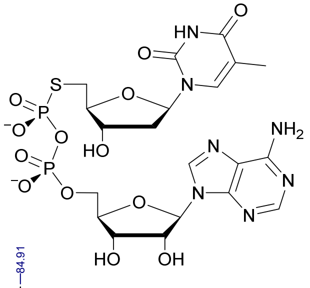

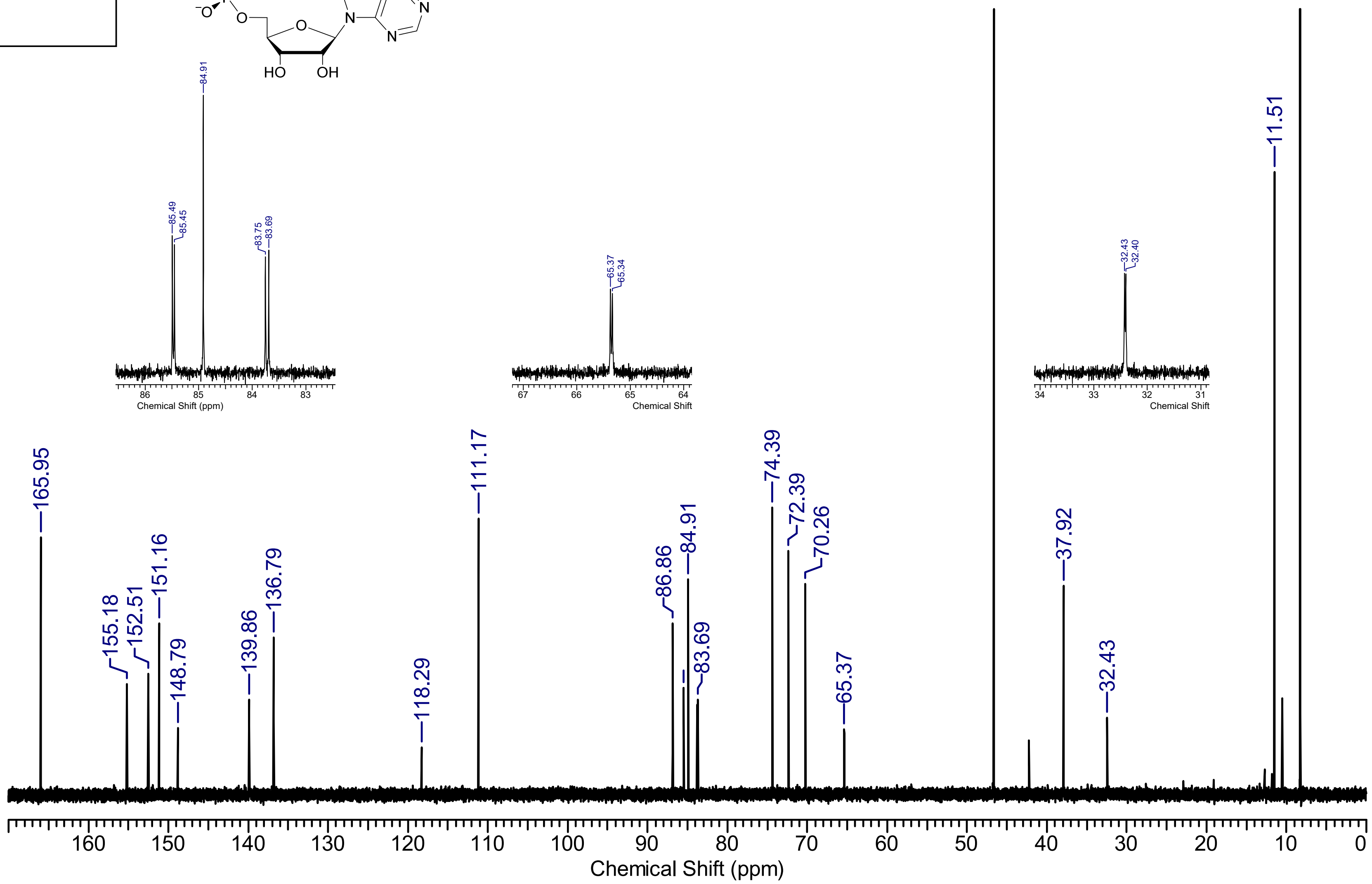

dTSppA –2b

^{13}C NMR DEPT135 151 MHz

D_2O

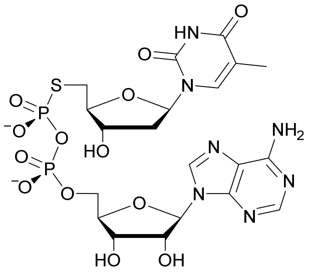

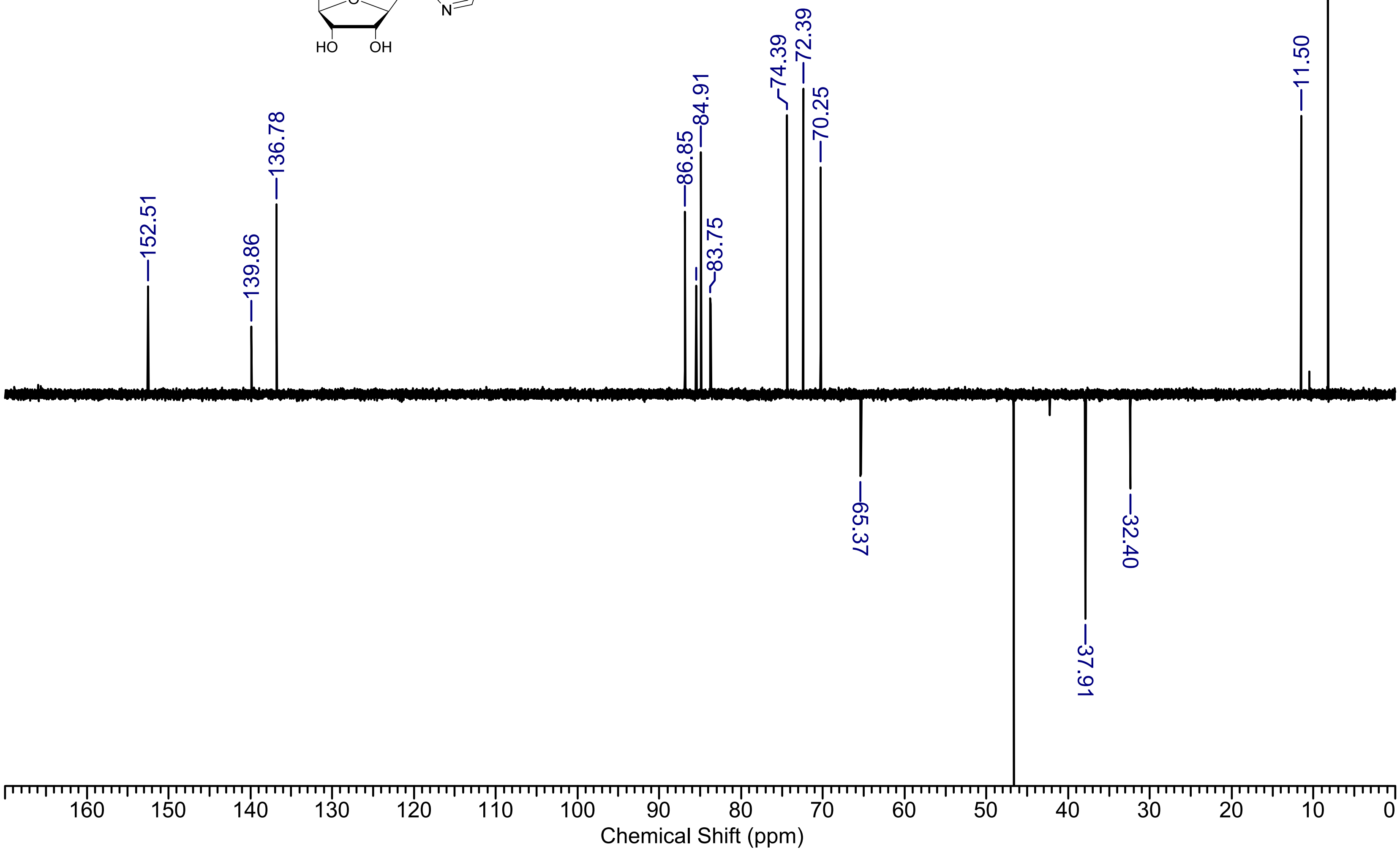

dTSppA – 2b

^{13}C-^{1}H HSQC 400 MHz

D_2O

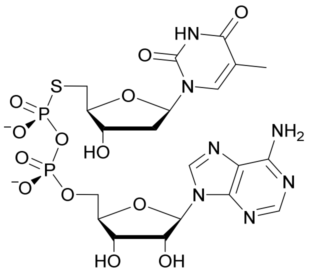

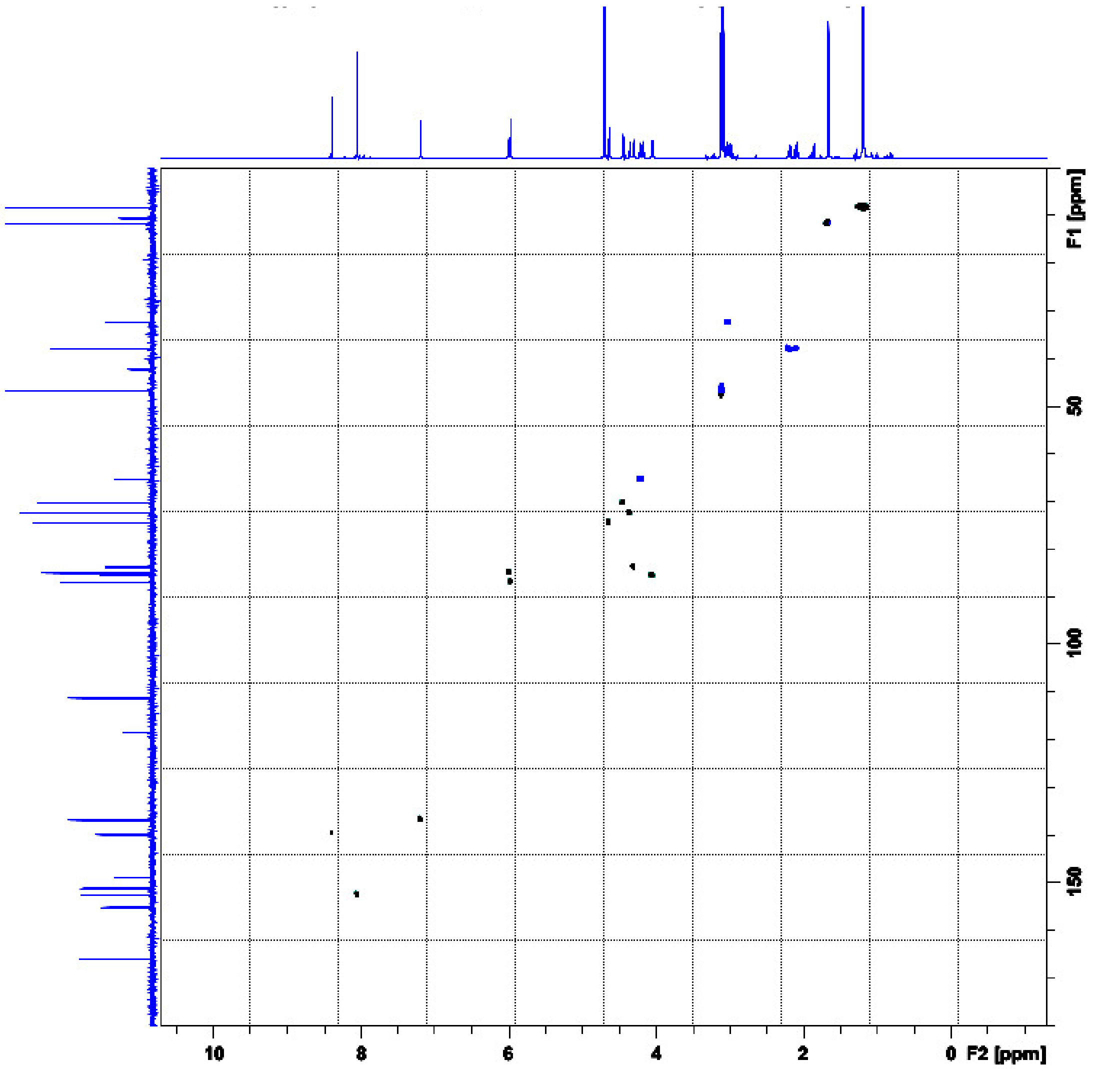

dTSppA – 2b

^{31}P NMR 162 MHz

D_2O

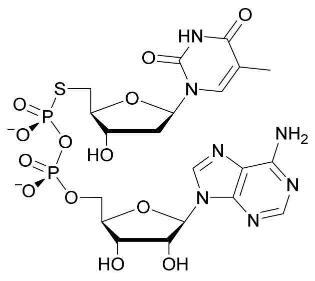

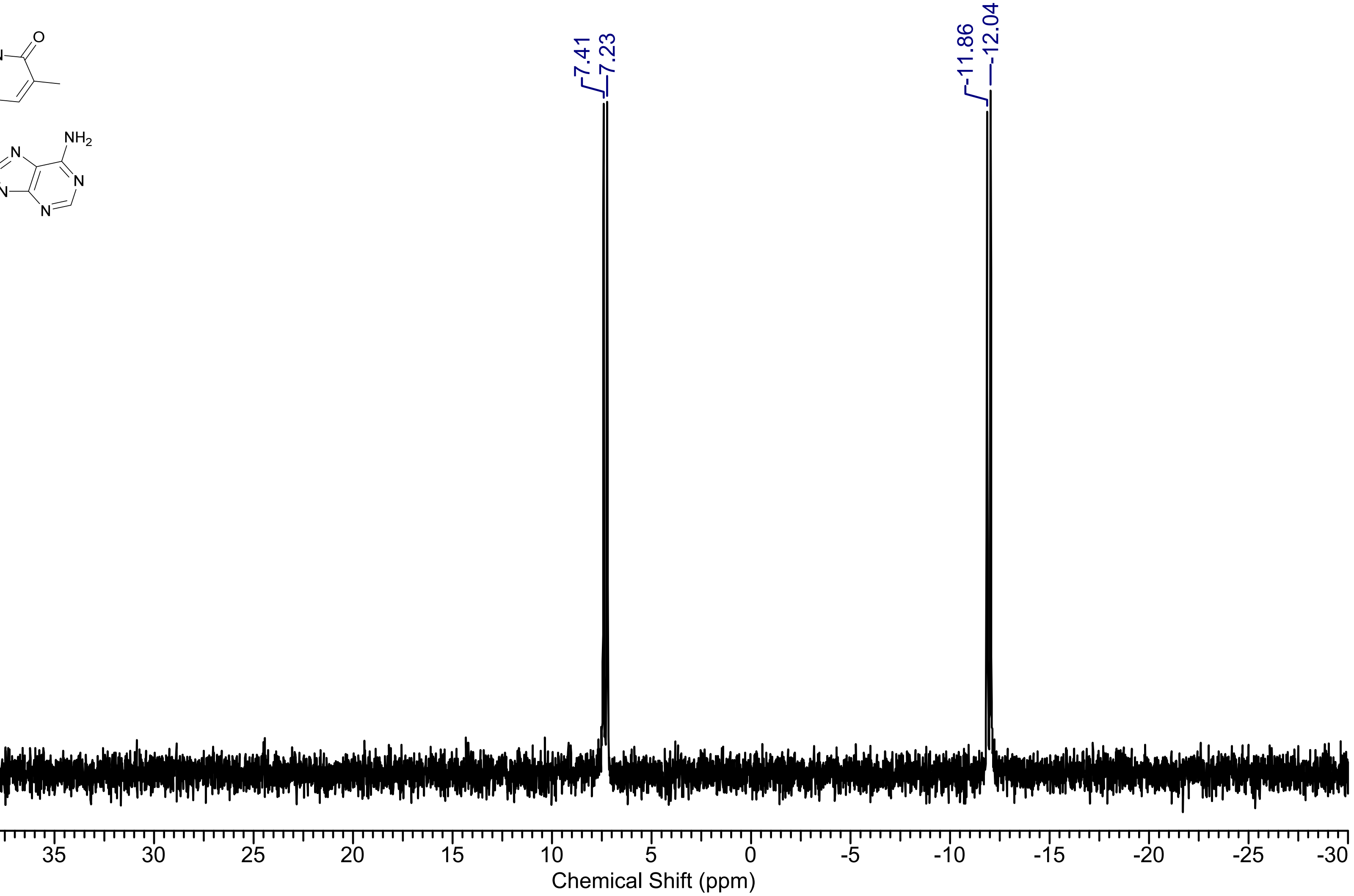

dTSppA – 2b

HRMS (ESI, negative ion)

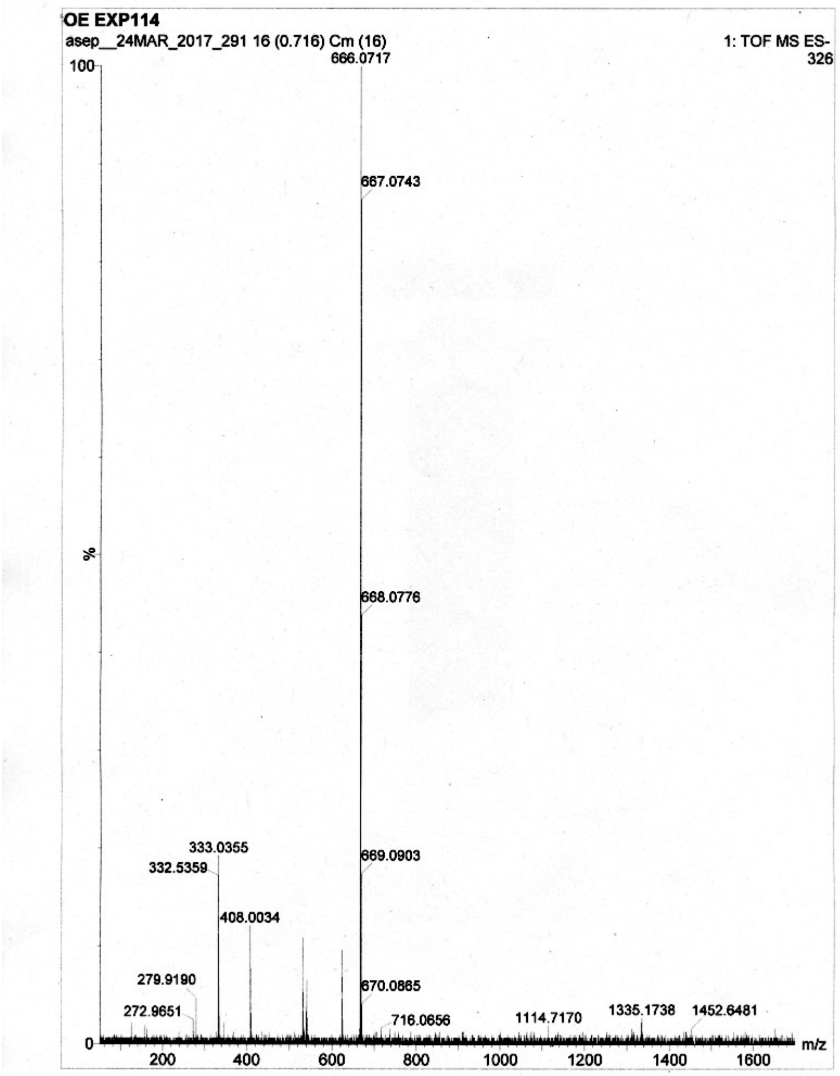

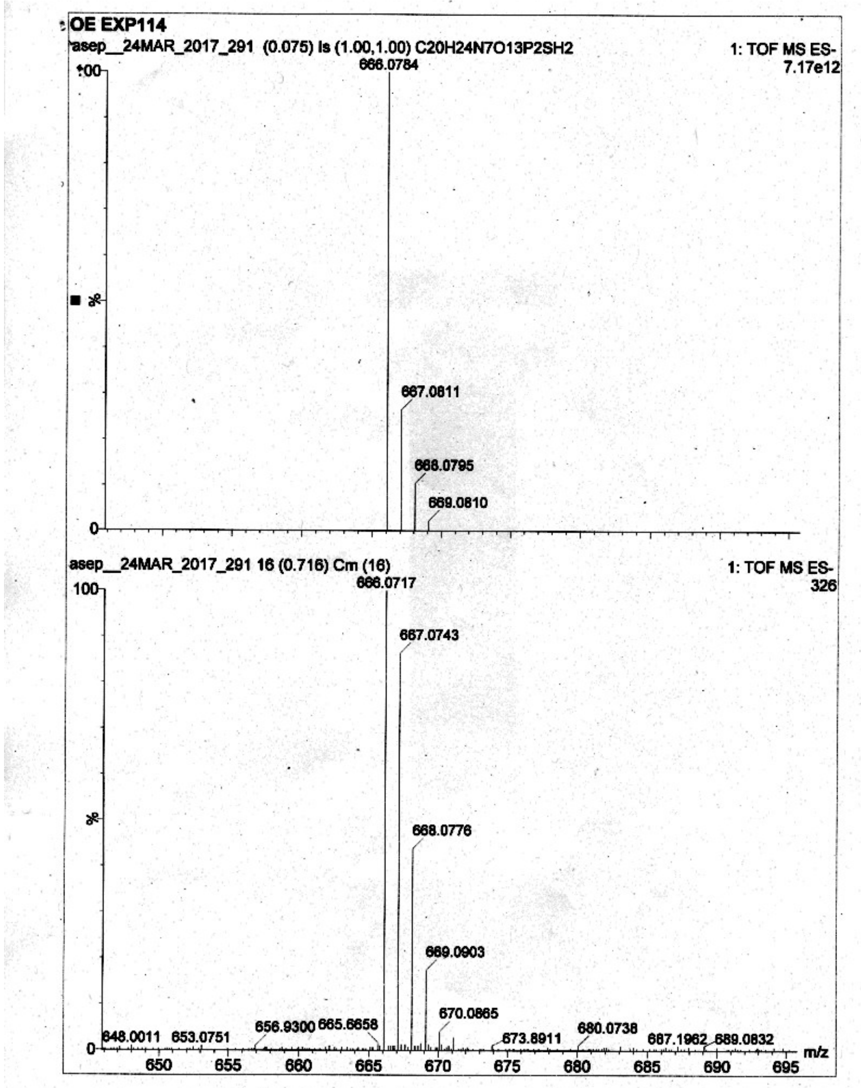

M-A reaction of NCSedT (6b)

^{31}P NMR 162 MHz

D_2O (external lock)

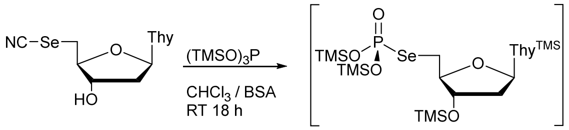

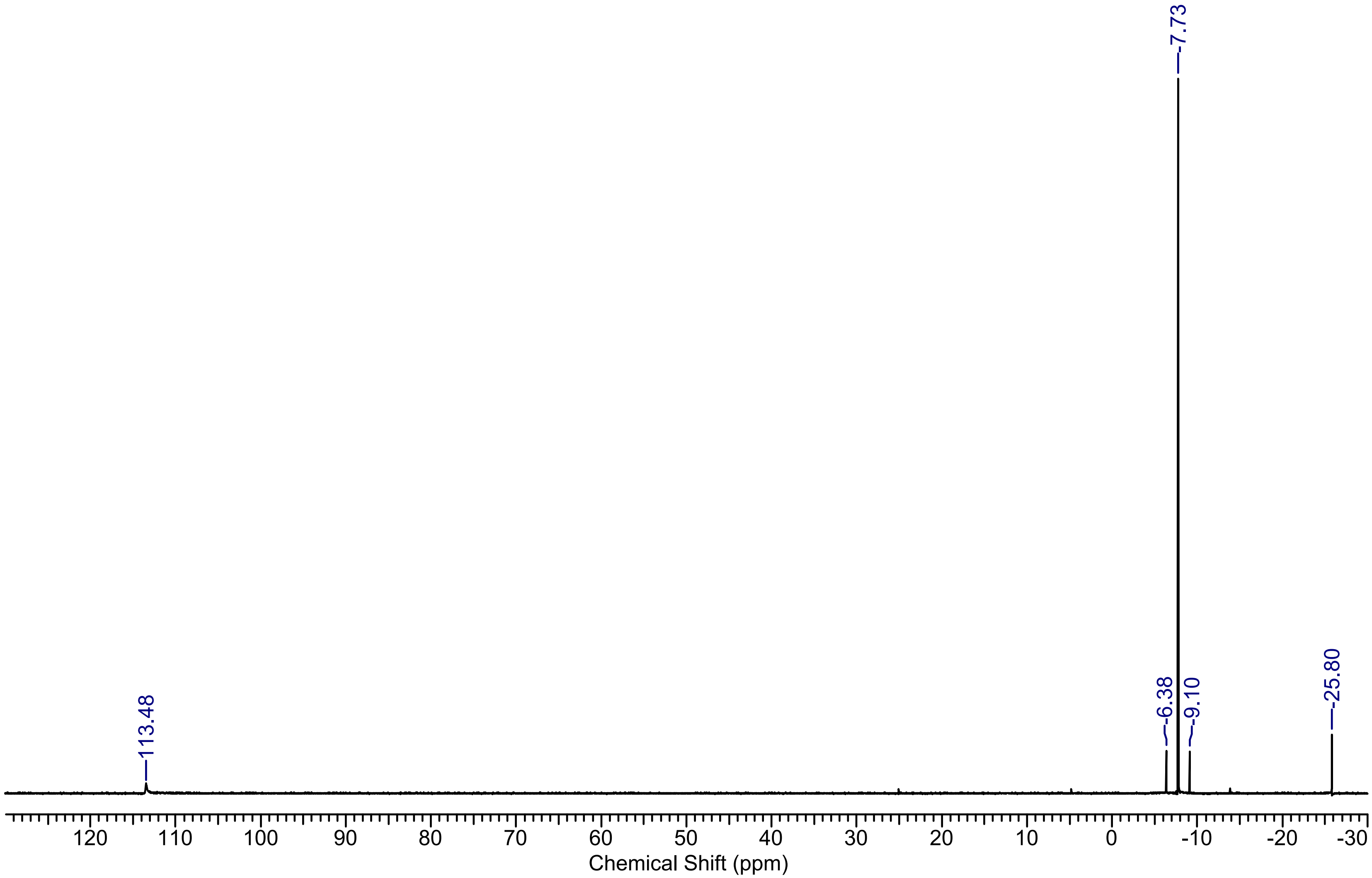

Crude phosphate coupling reaction (dTSeppA: 2b)

^{31}P NMR 162 MHz

D_2O (external lock)

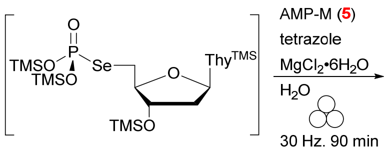

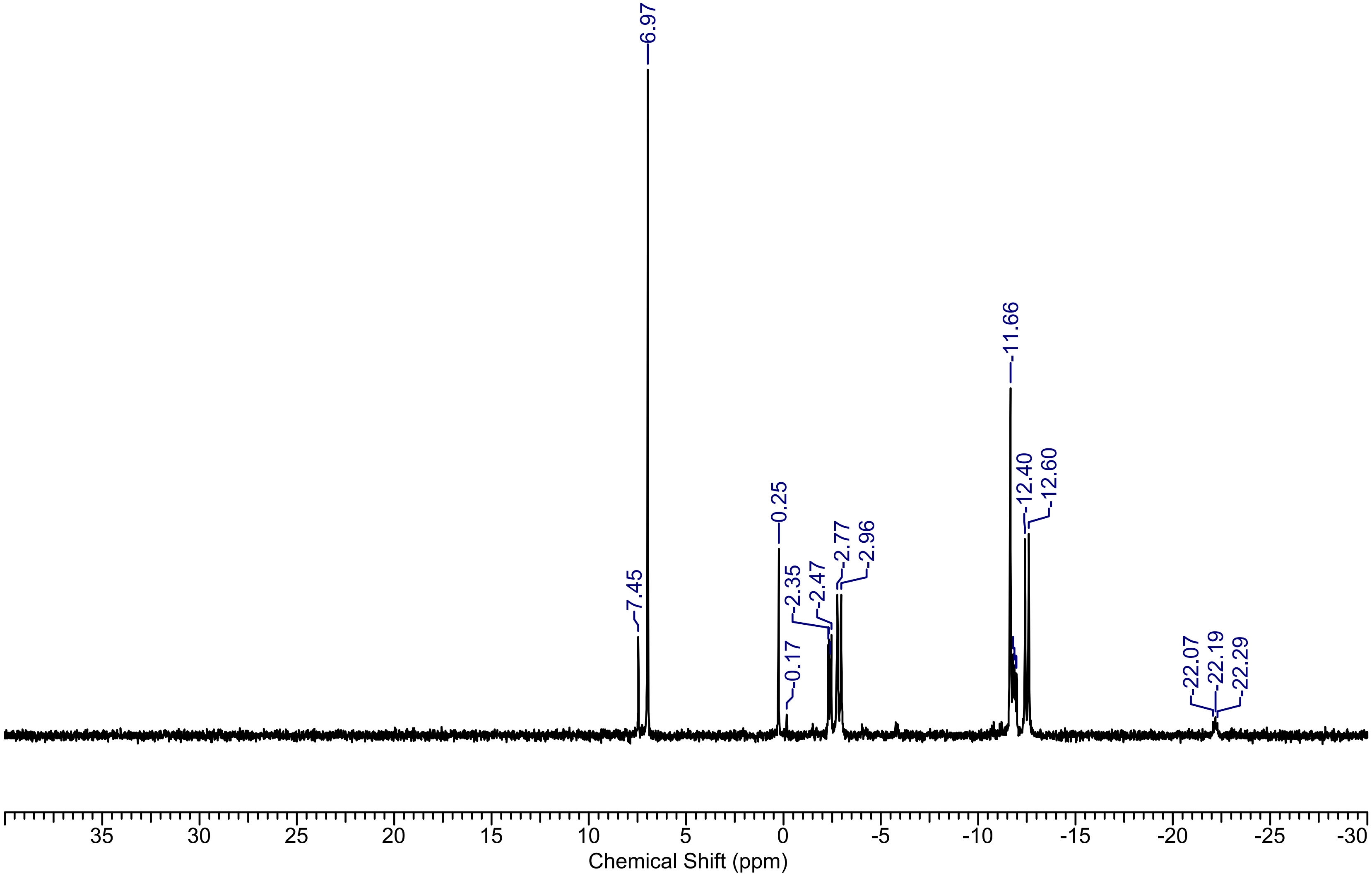

Analytical C18 RP-HPLC of crude dTSeppA reaction mixture - gradient G1

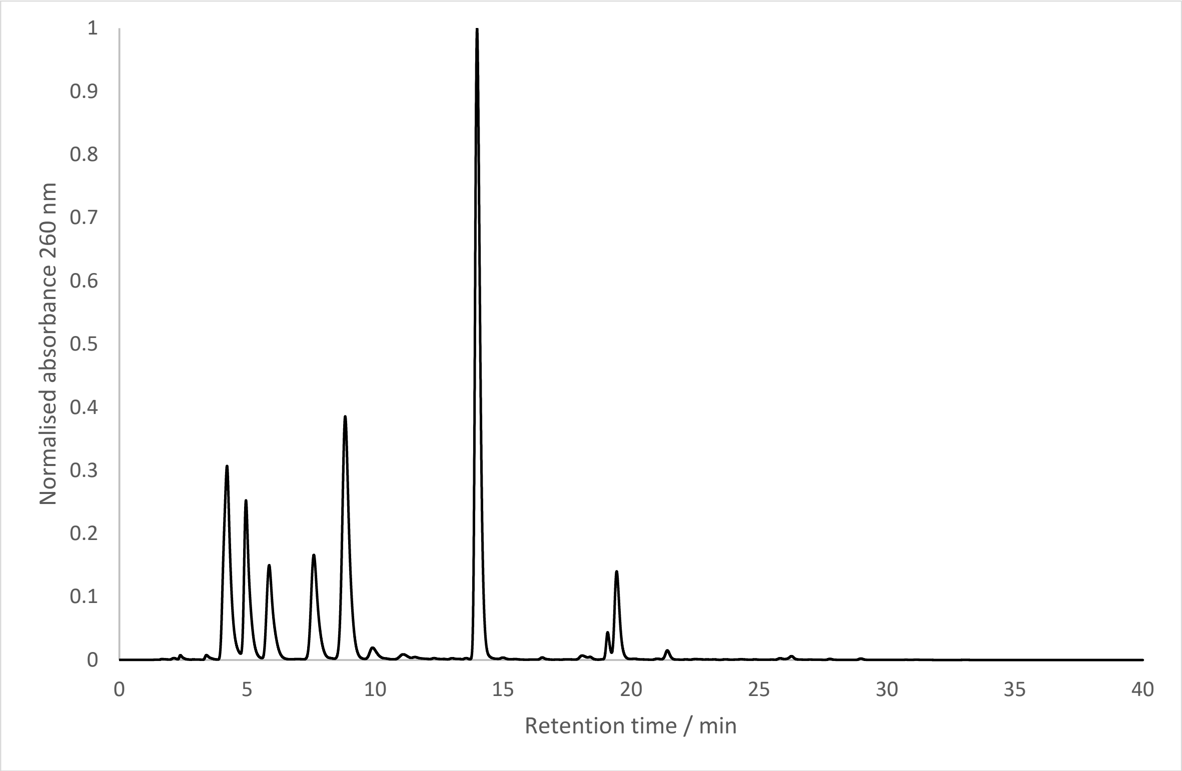

Analytical C18 RP-HPLC of pure dTSeppA (2b) – gradient G1

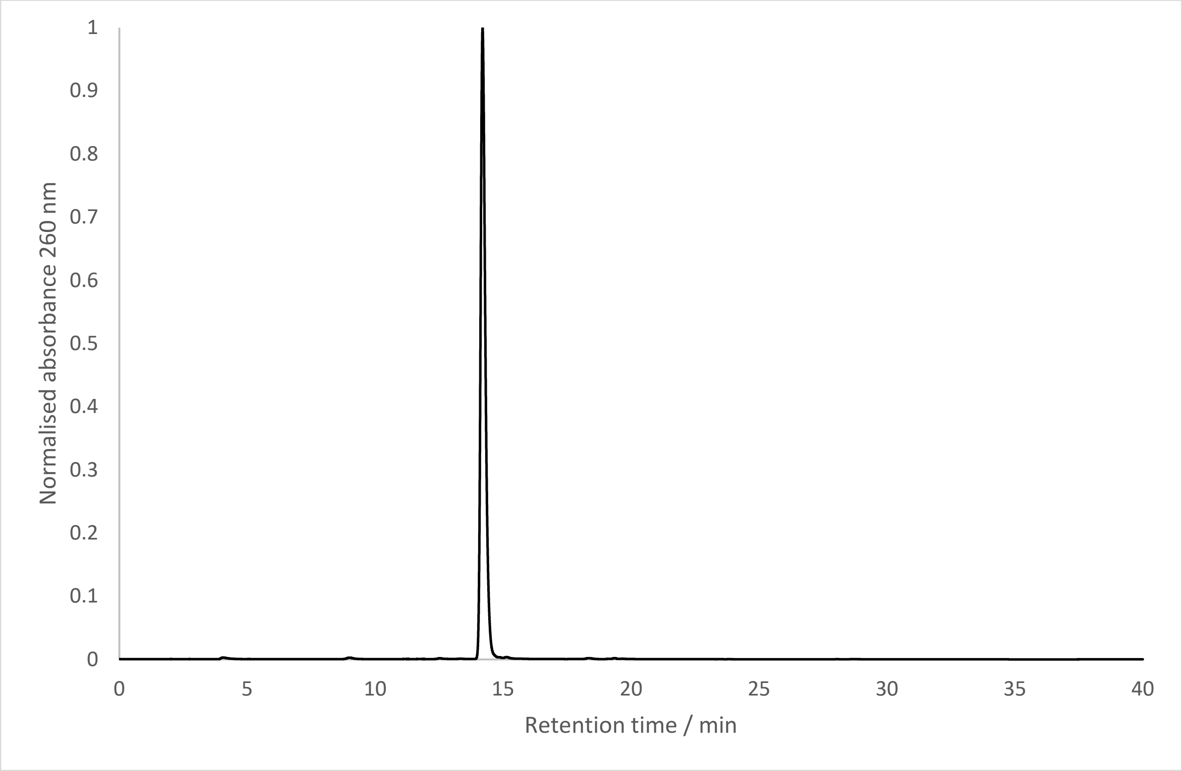

dTSeppA – 2b

^{1}H NMR 600 MHz

D_2O

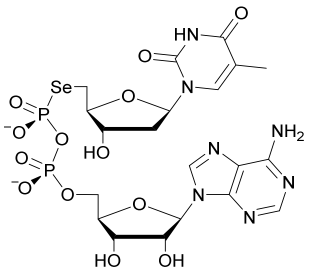

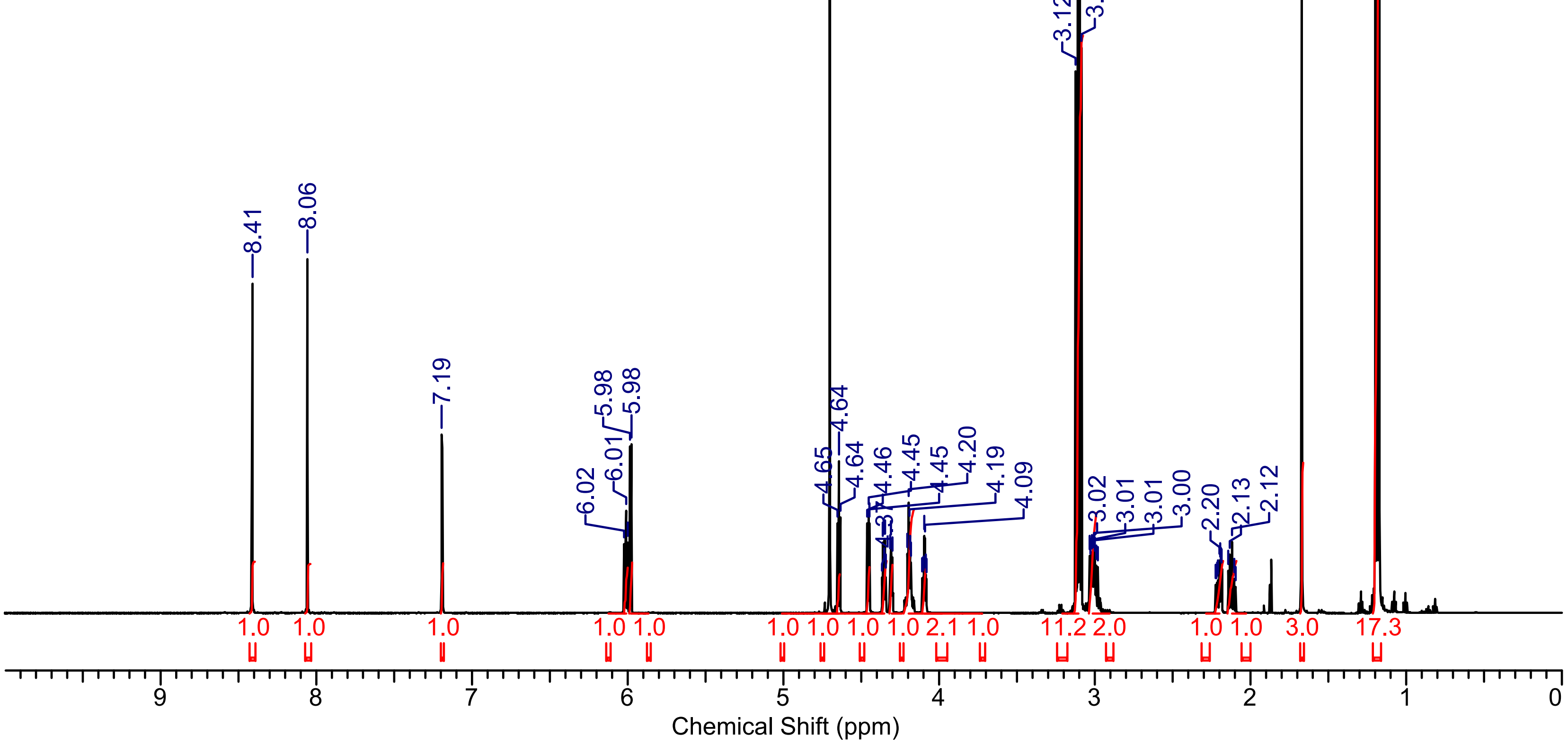

dTSeppA – 2b

^{1}H-^{1}H COSY 600 MHz

D_2O

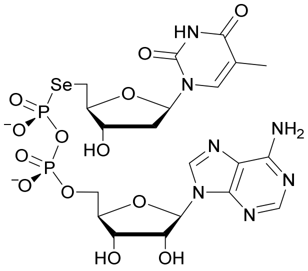

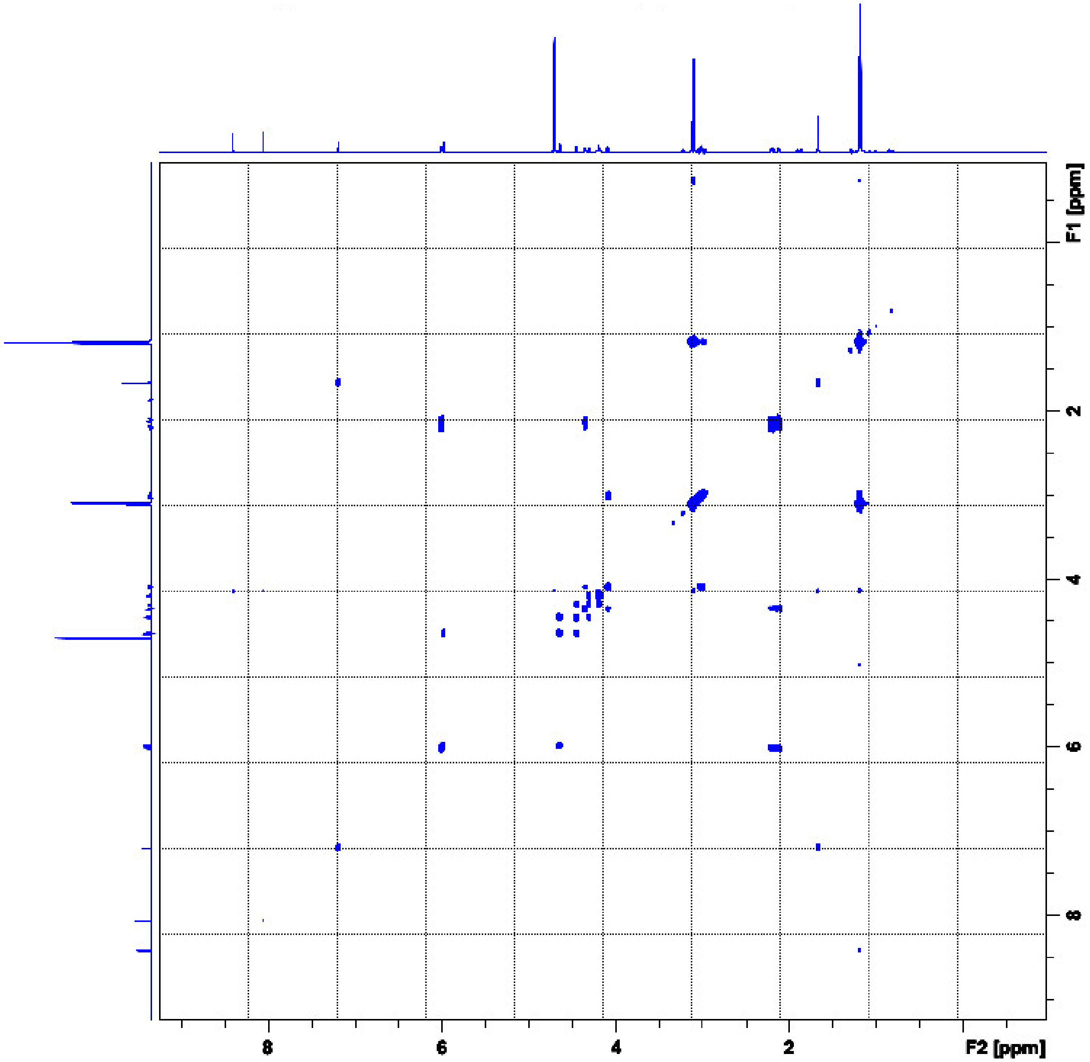

dTSeppA – 2b

^{1}H-^{1}H COSY 600 MHz D$_2$O

(7.0 – 2.0 ppm expansion)

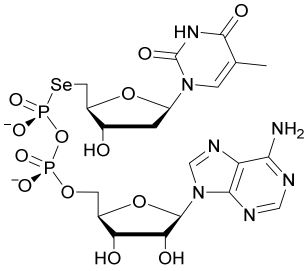

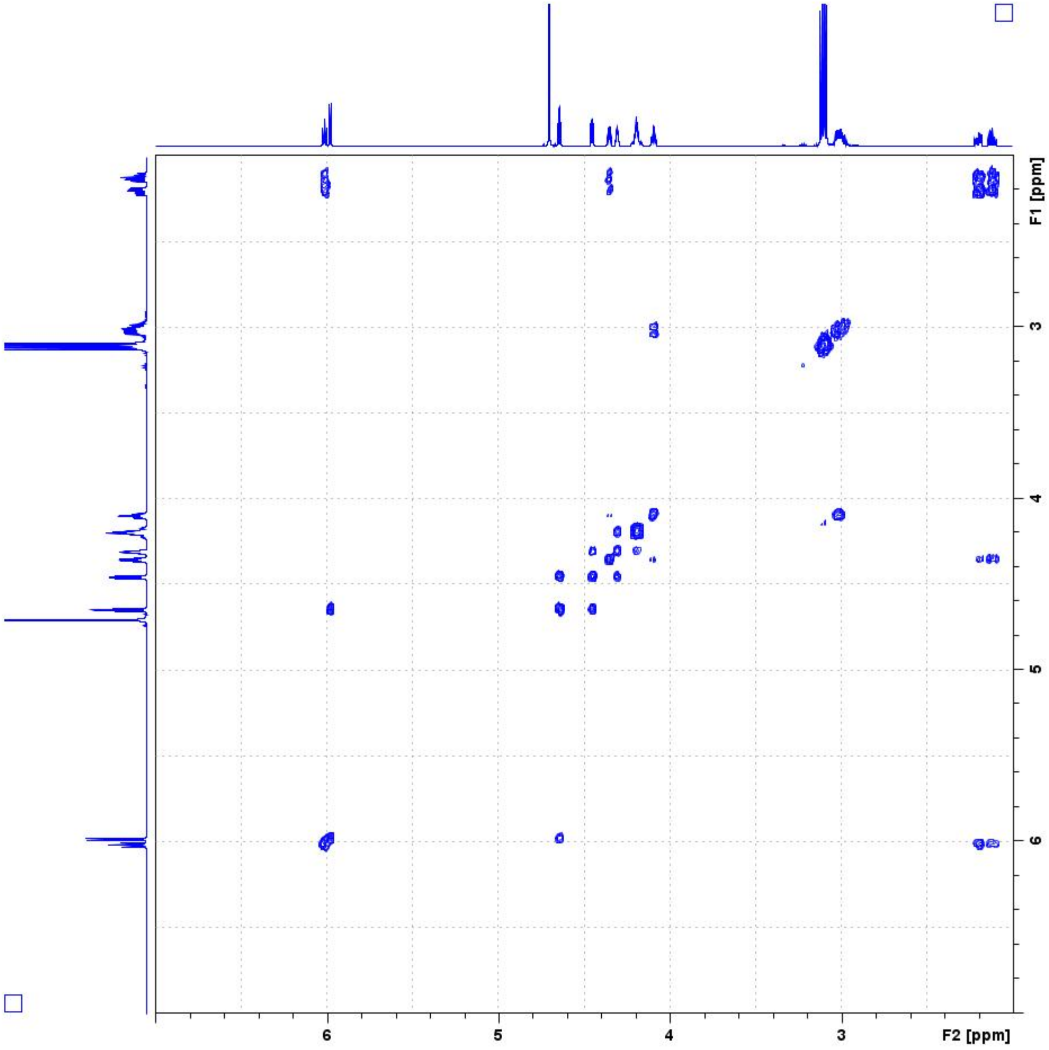

dTSeppA – 2b

^{13}C NMR 151 MHz

(with expansions of P-C couplings)

D_2O

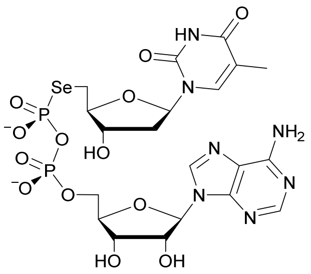

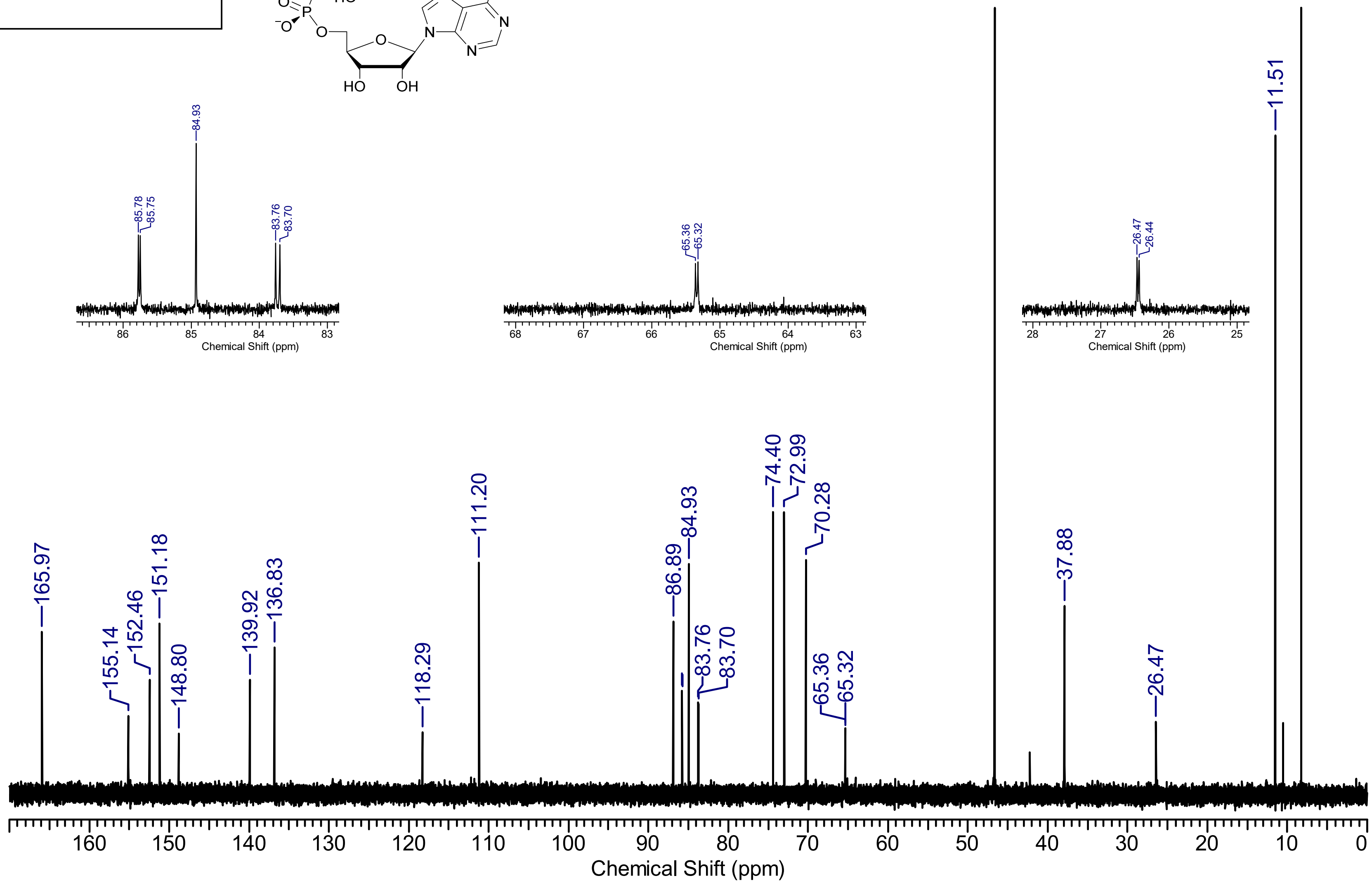

dTSeppA – 2b

^{13}C NMR DEPT135

151 MHz

D_2O

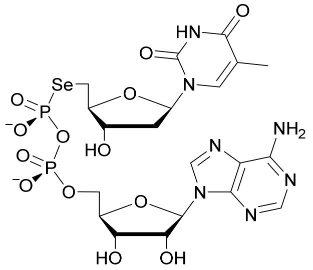

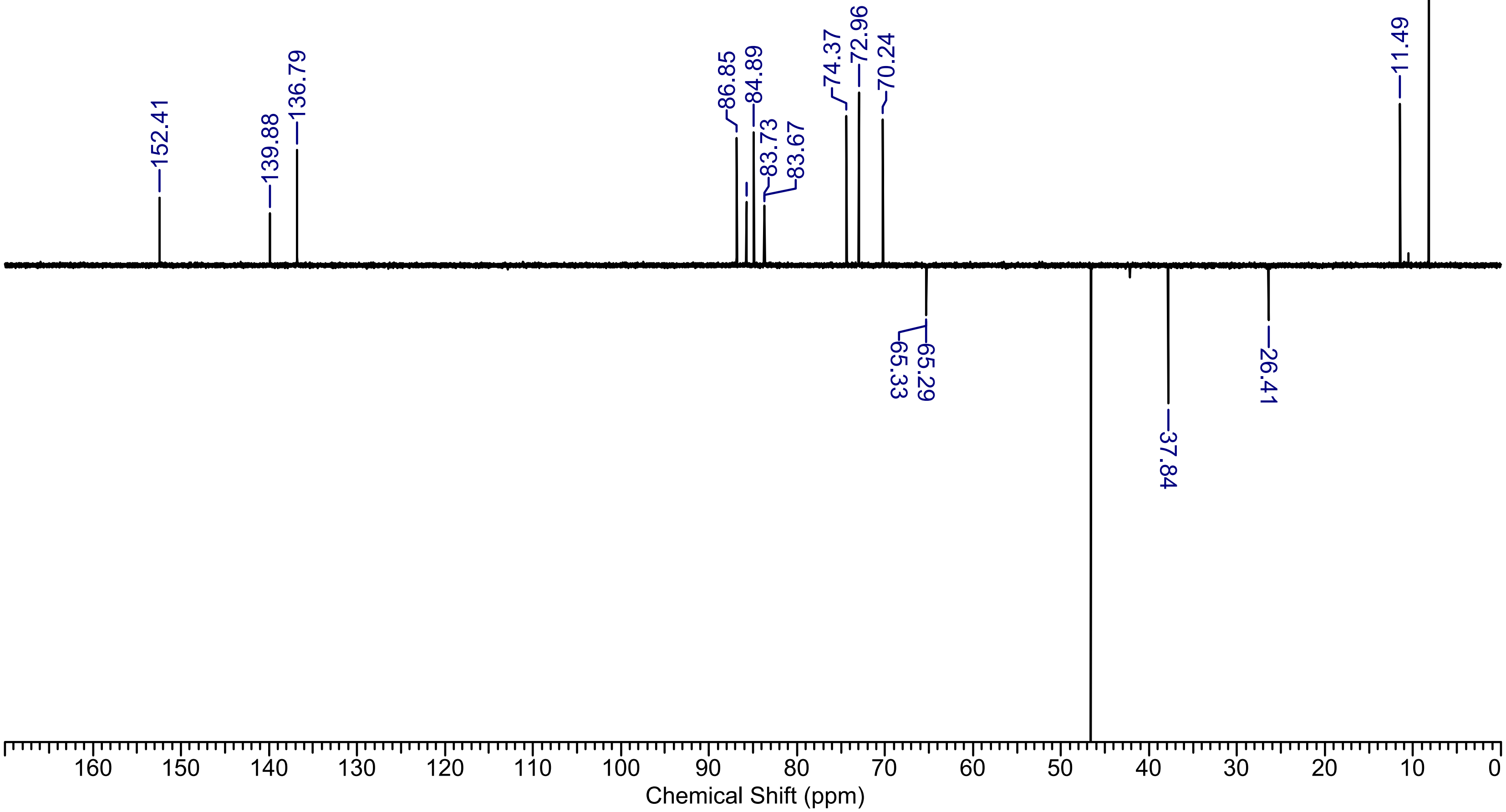

dTSeppA – 2b

^{13}C-^{1}H HSQC 600 MHz

D_2O

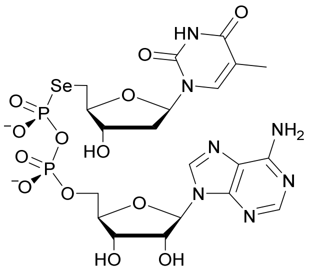

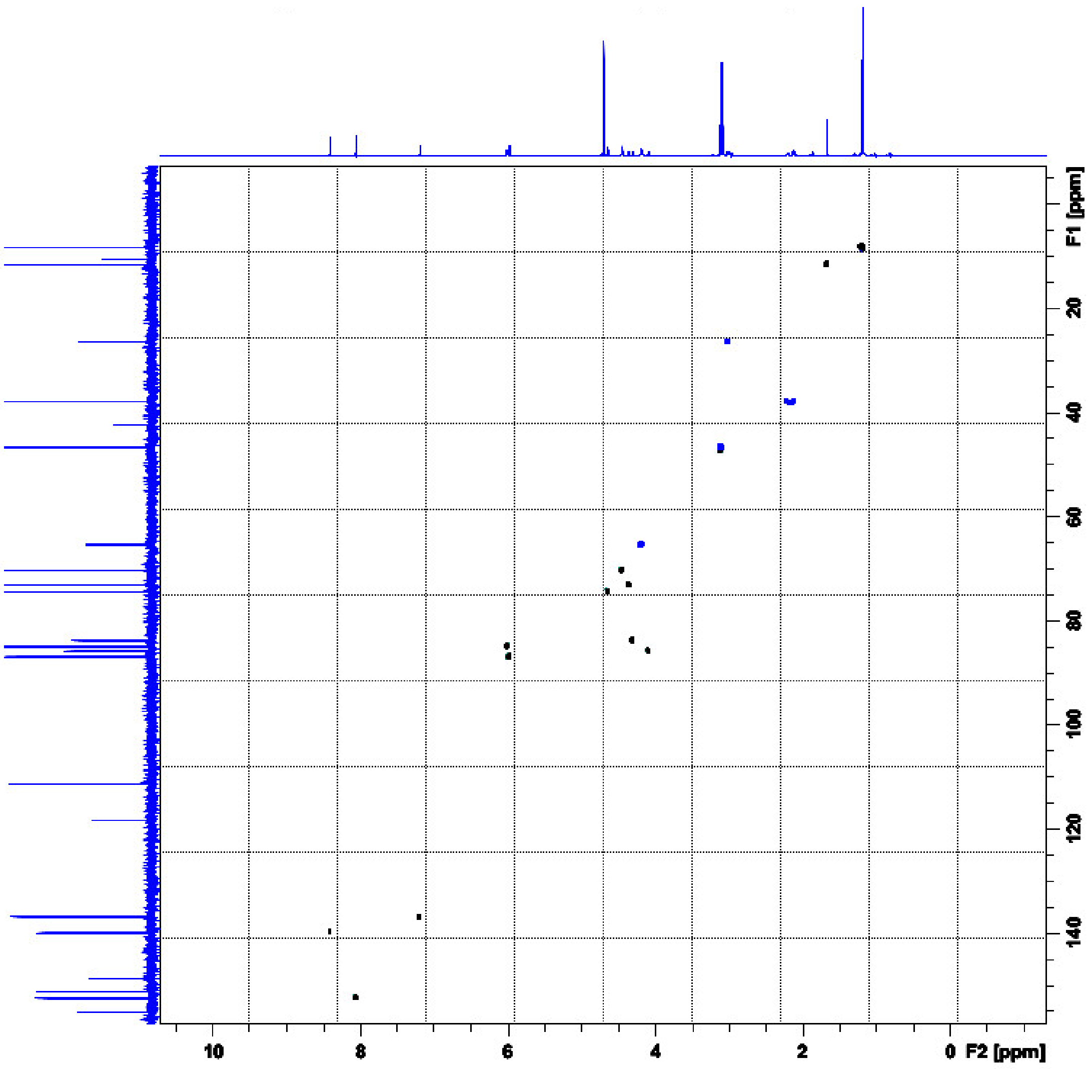

dTSeppA – 2b

^{31}P NMR 243 MHz

D_2O

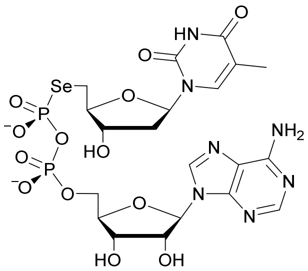

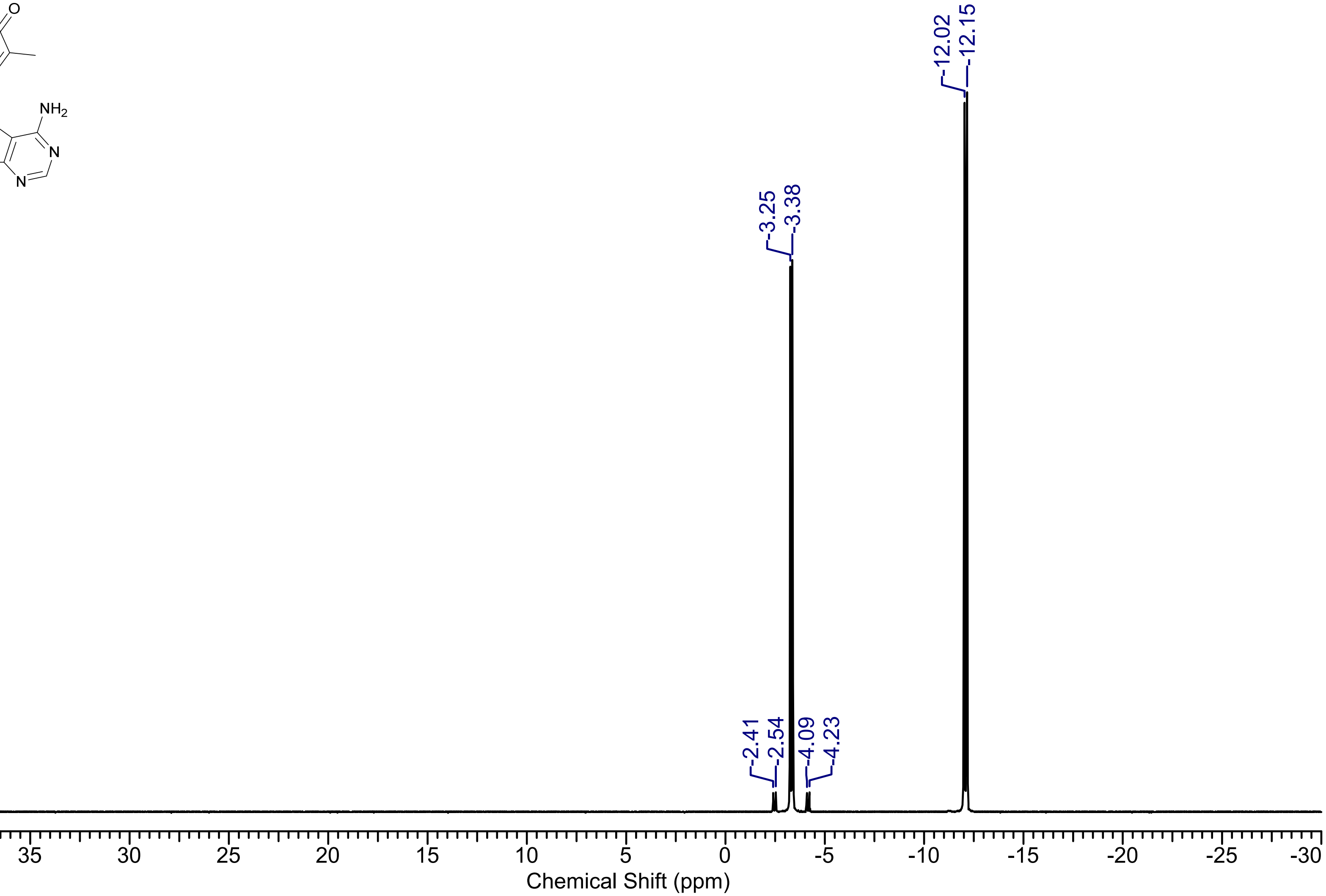

dTSeppA – 2b
(+0.25M KSeCN in D2O)
^{77}Se NMR 114 MHz
D2O

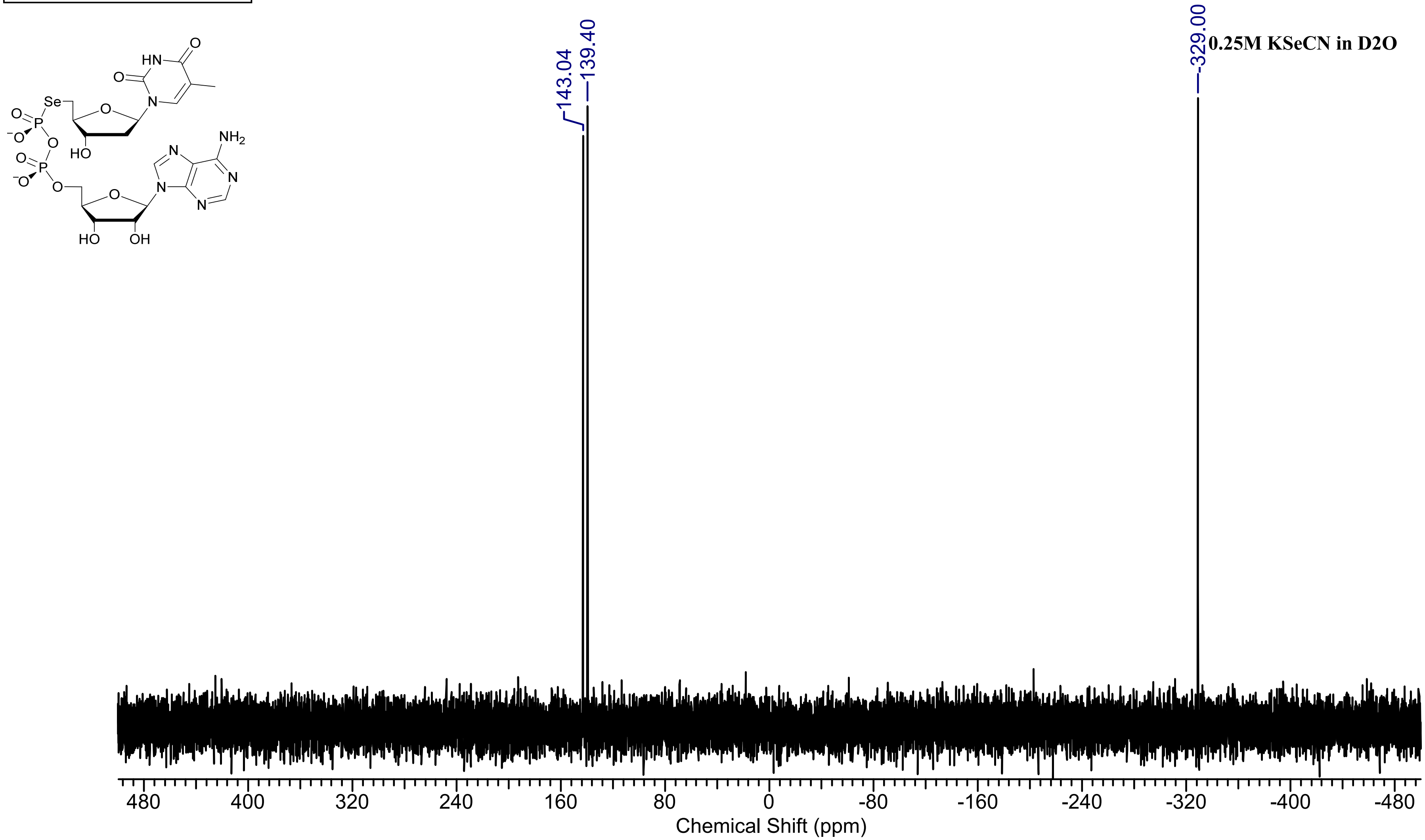

dTSeppA – 2b

HRMS (ESI, negative ion)

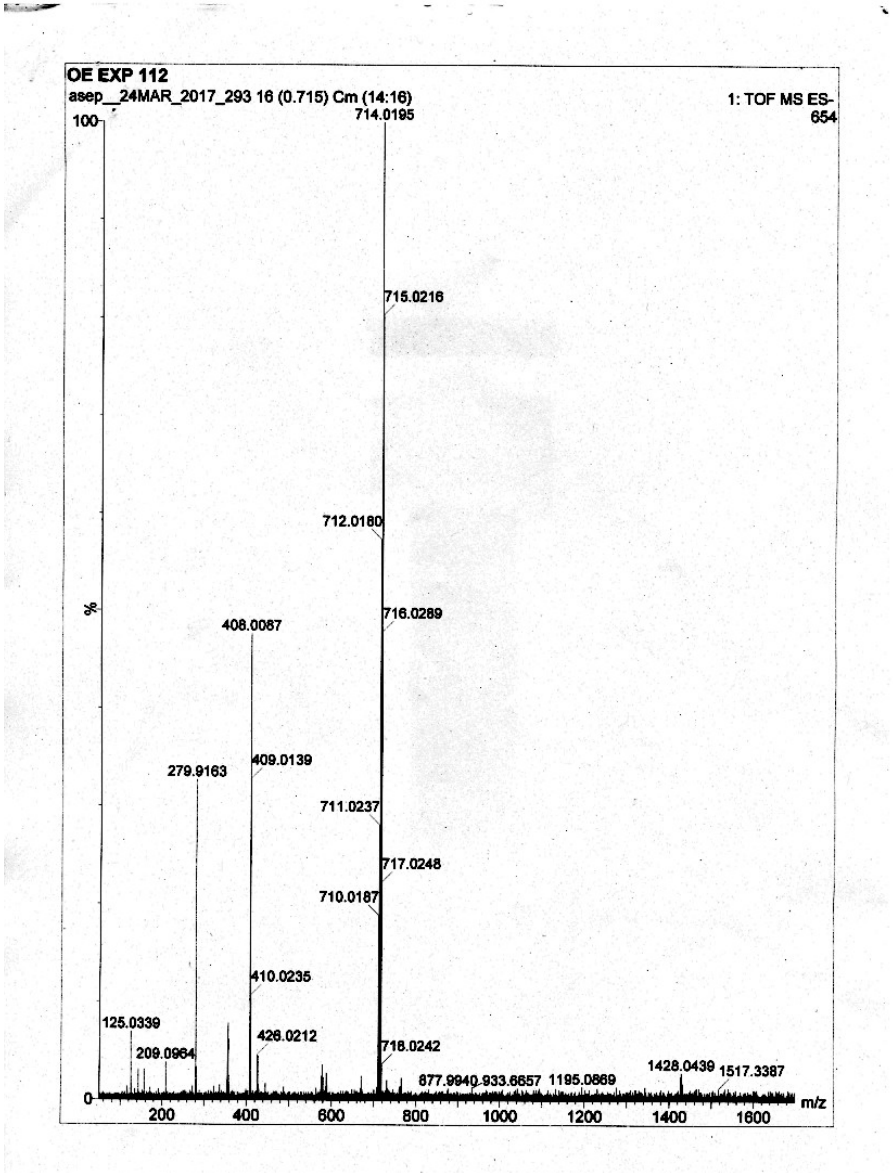

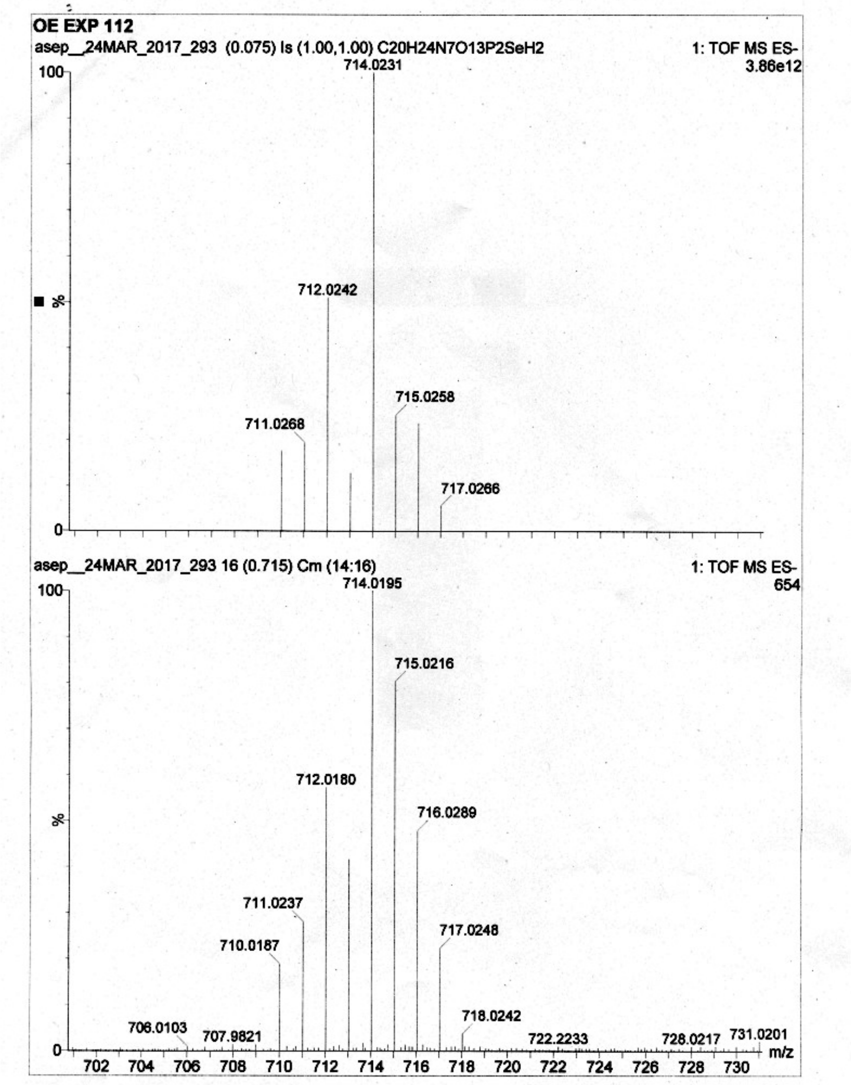

NPySSdT – 6a

^{1}H NMR 400 MHz

D_6-DMSO

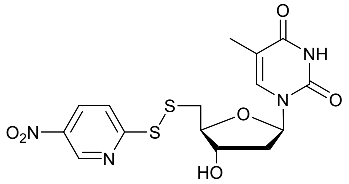

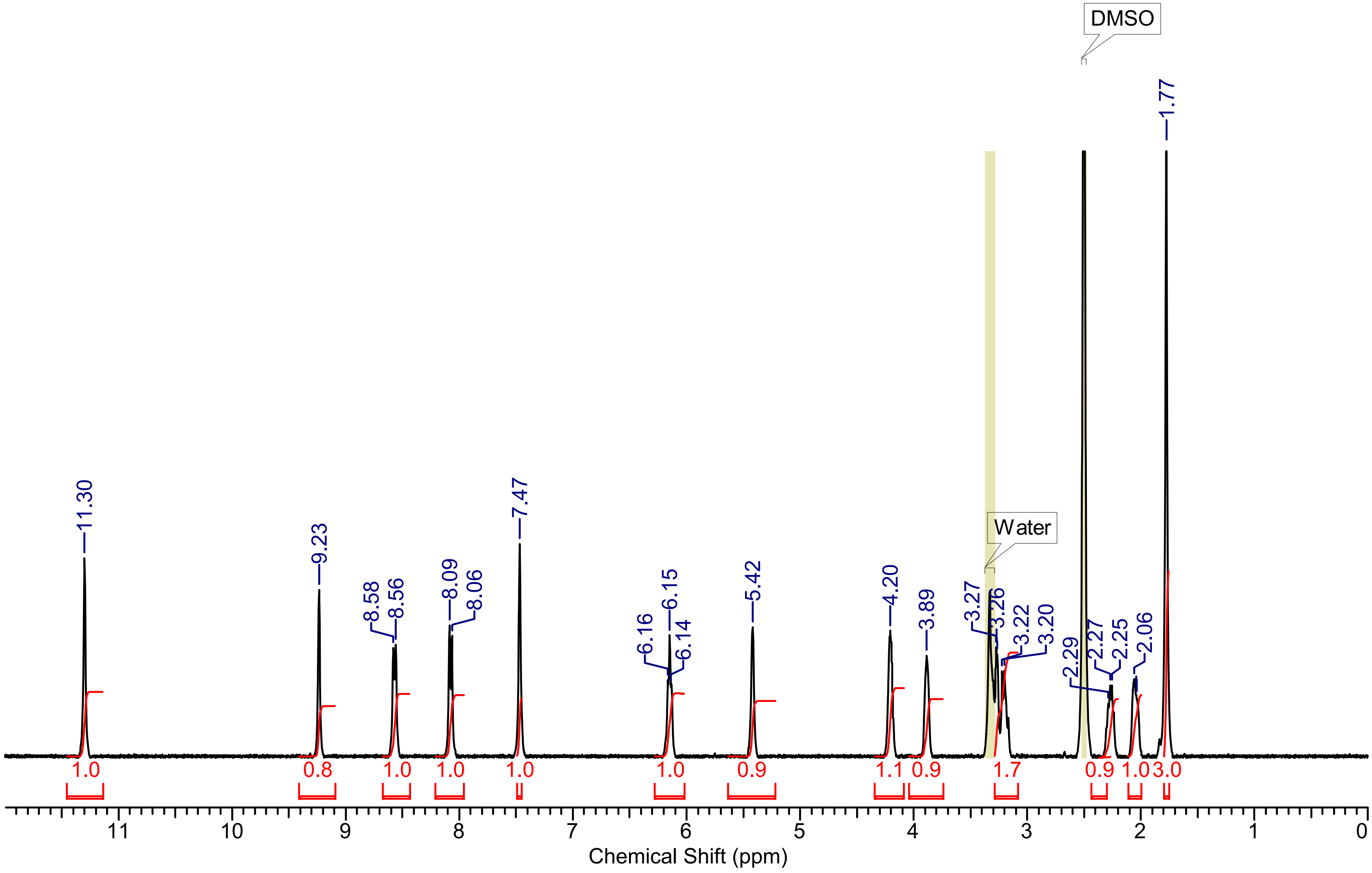

NPySSdT – 6a
^{13}C NMR 101 MHz
D_6-DMSO

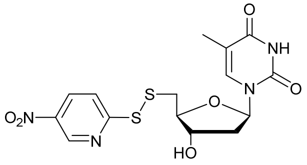

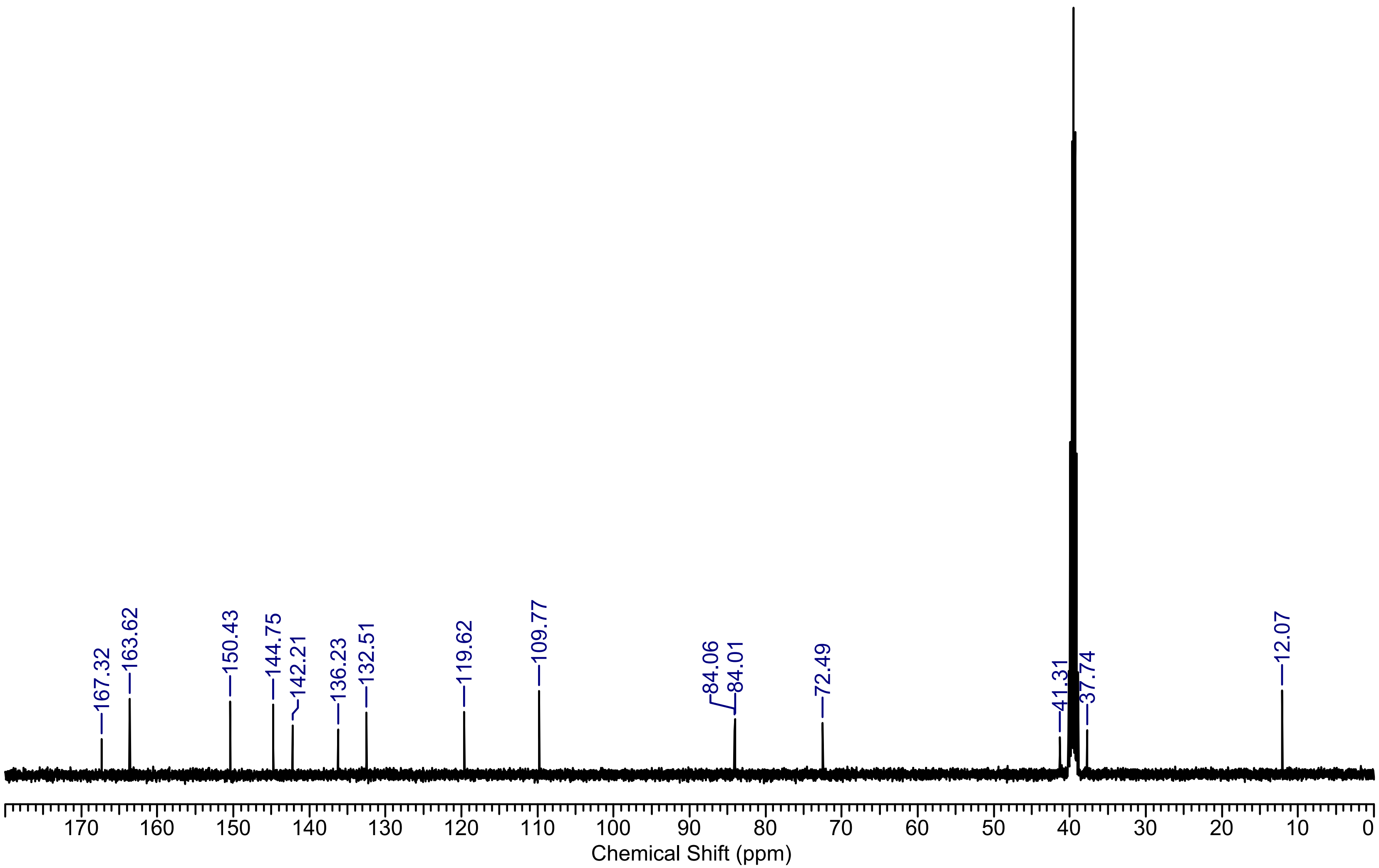

NPySSdT – 6a

HRMS (ESI, positive ion)

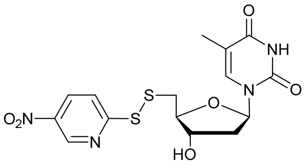

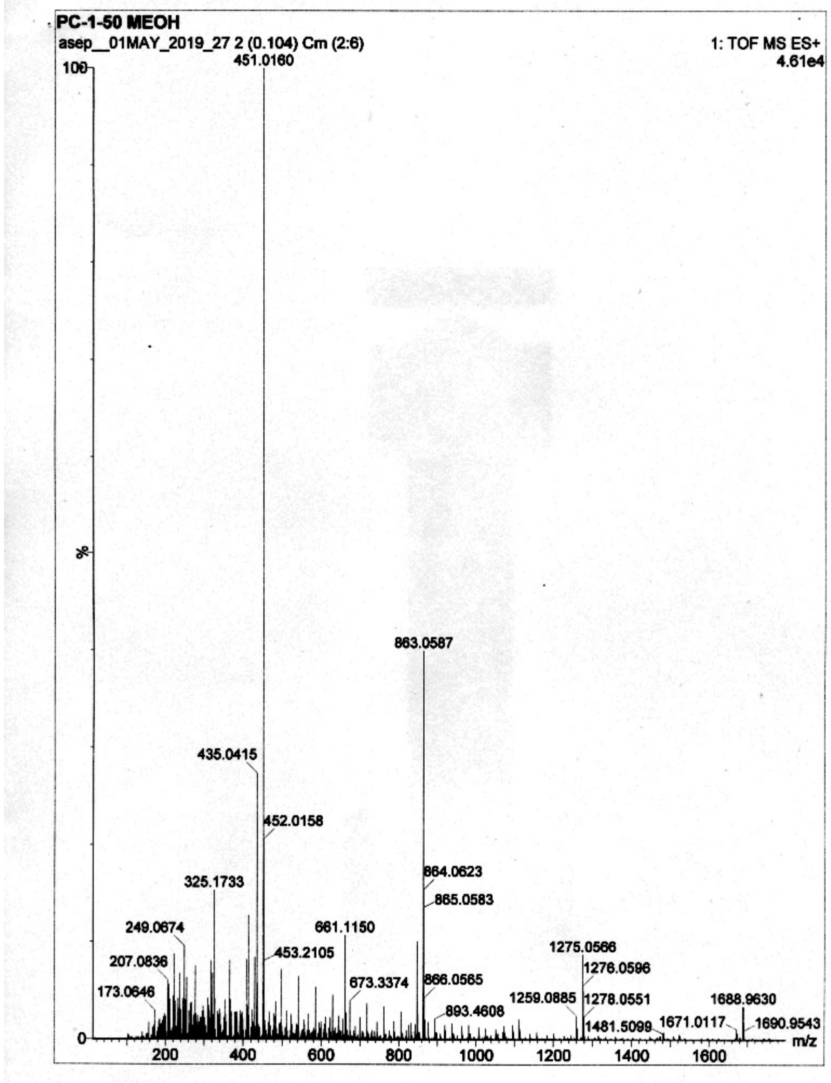

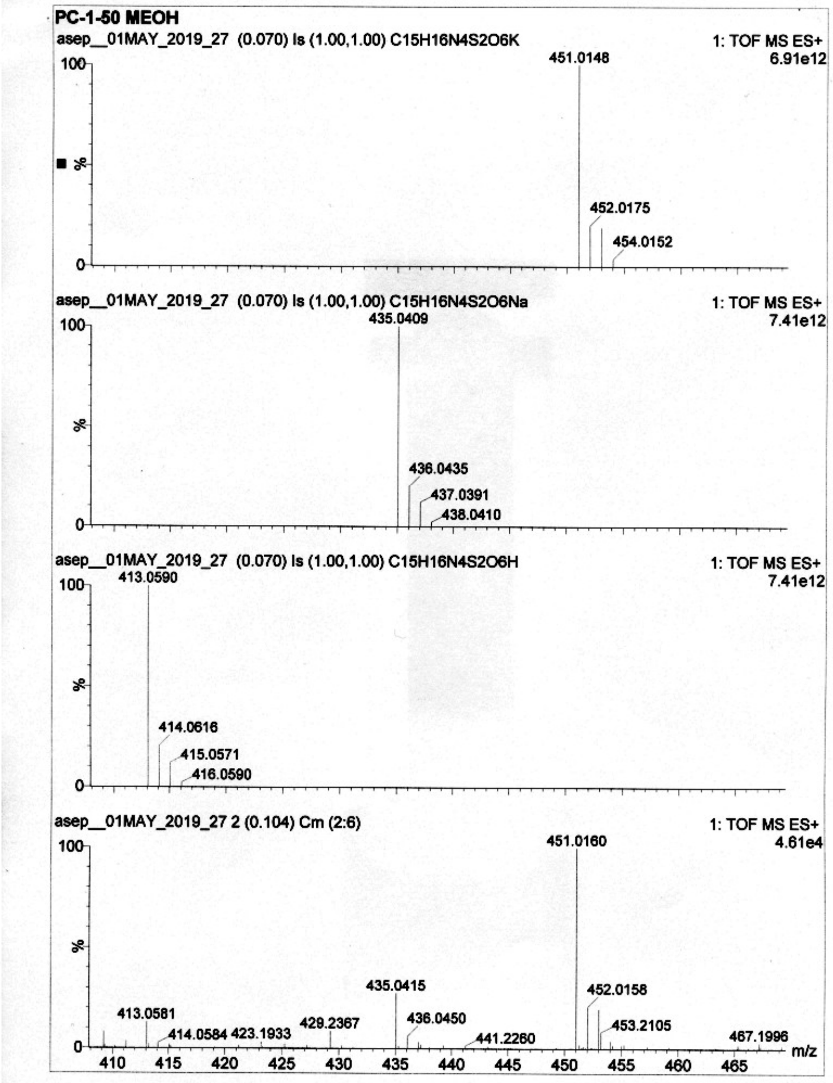

Table 2: Model fits to ^{31}P NMR data in nucleoside compounds dAChppA

dASppA 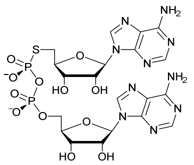 **1a**	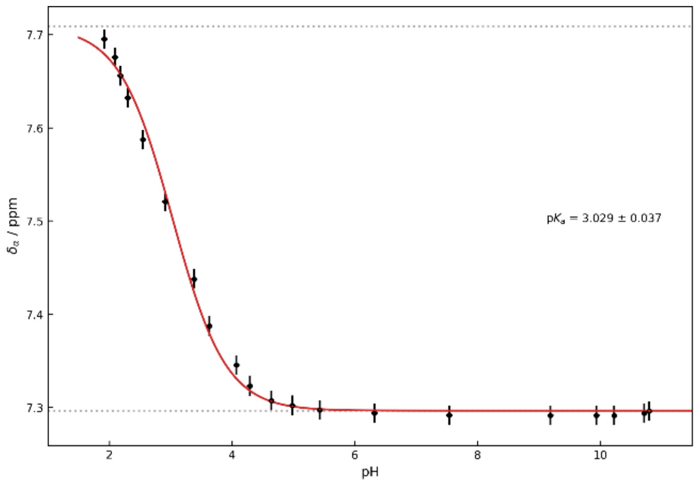	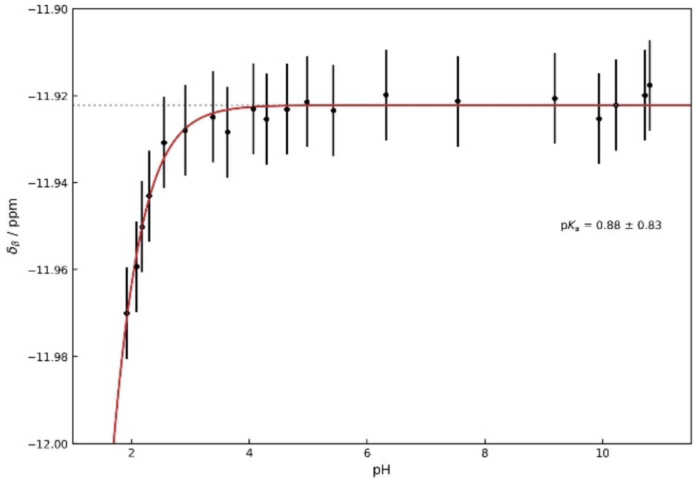
dASeppA 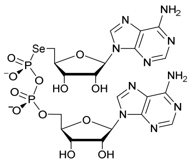 **1b**	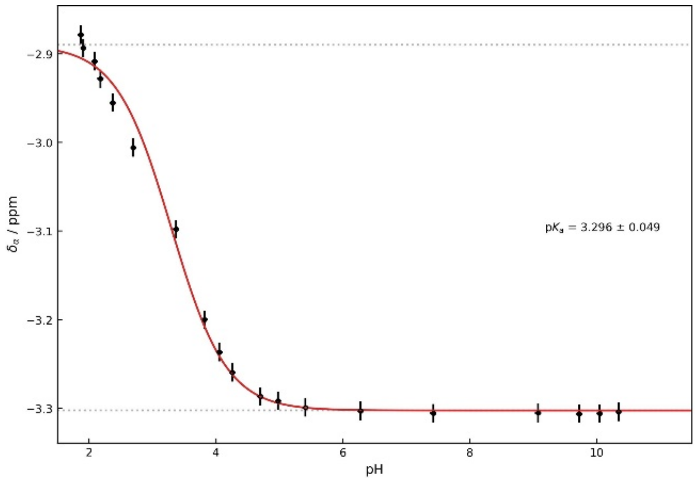	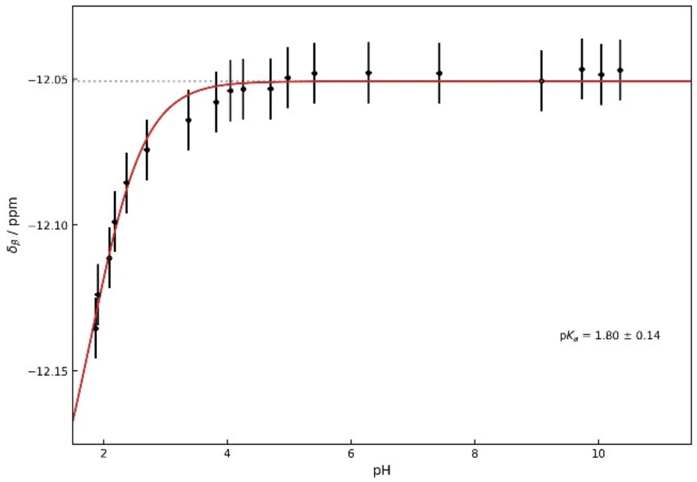

Table 2: Model fits to ^{31}P NMR data in nucleoside compounds dTChppA

dTSppA<br>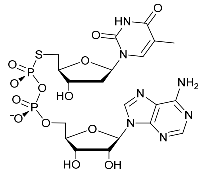<br>**2a**	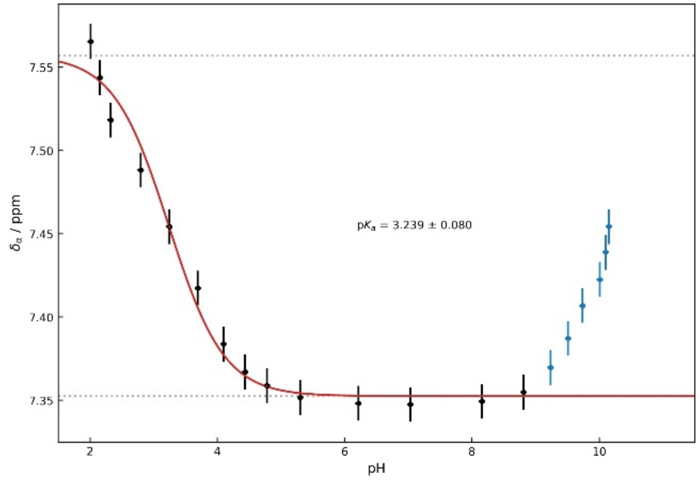 	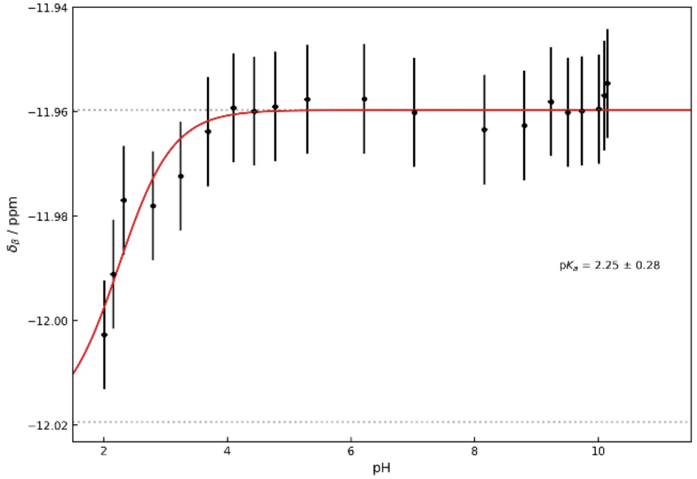
dTSeppA<br>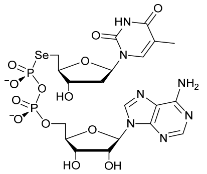<br>**2b**	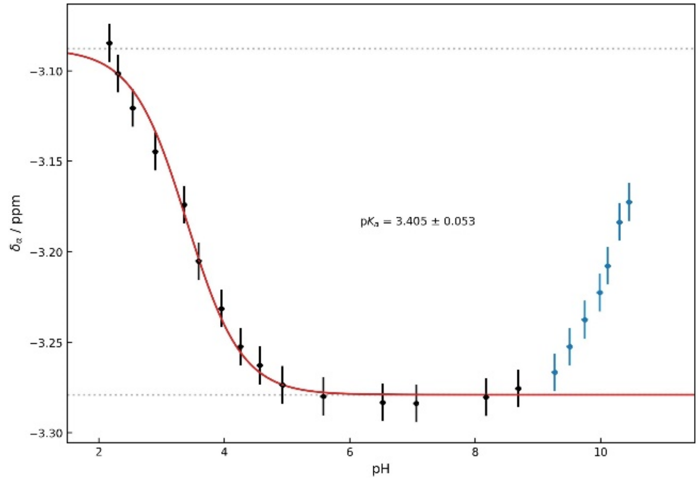 	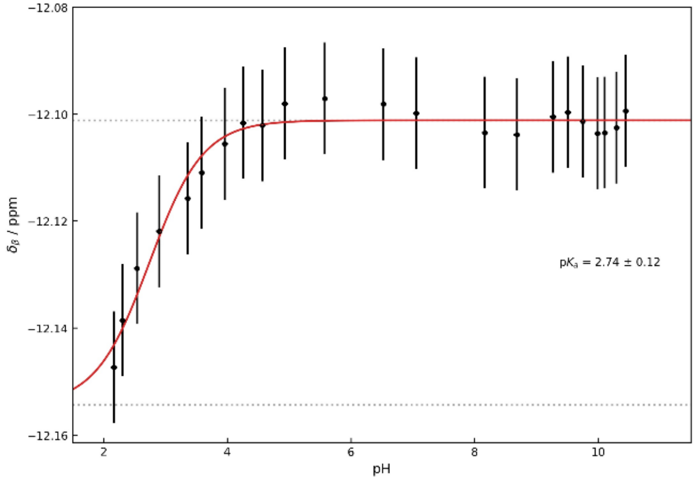

dASppA (1a) pH titration NMR series
^{31}P NMR 202 MHz

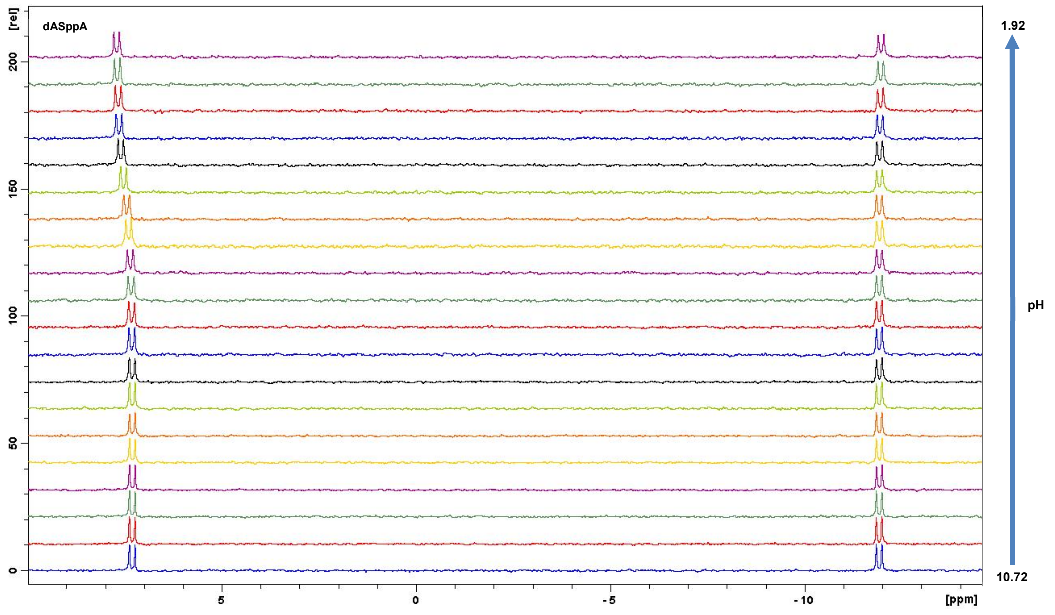

dASeppA (1b) pH titration NMR series
^{31}P NMR 202 MHz

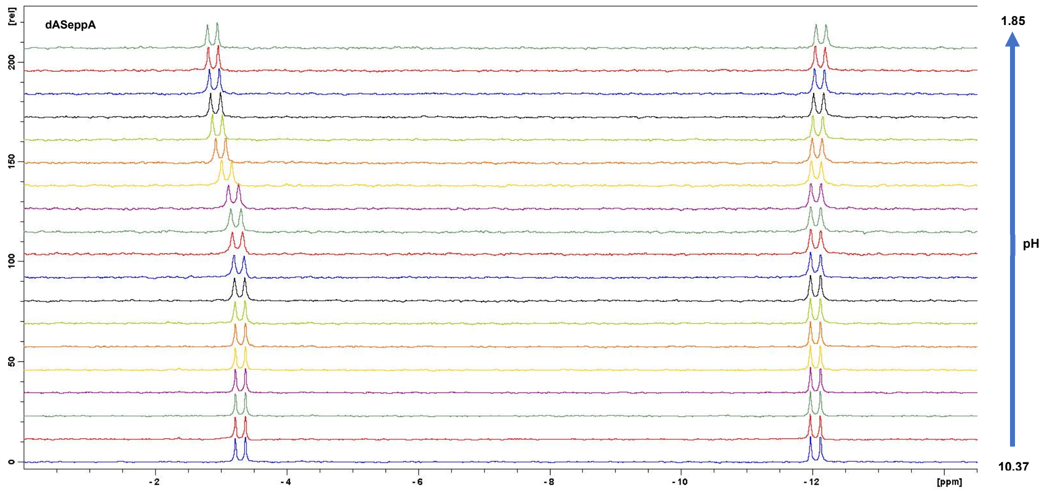

dTSppA (2a) pH titration NMR series

^{31}P NMR 202 MHz

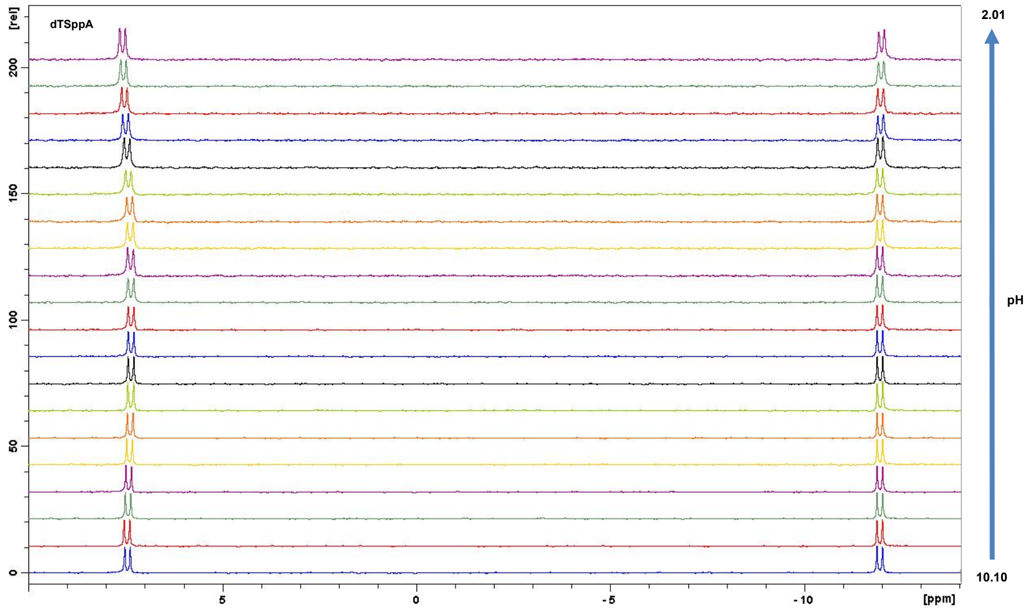

dTSeppA (2b) pH titration NMR series

^{31}P NMR 202 MHz

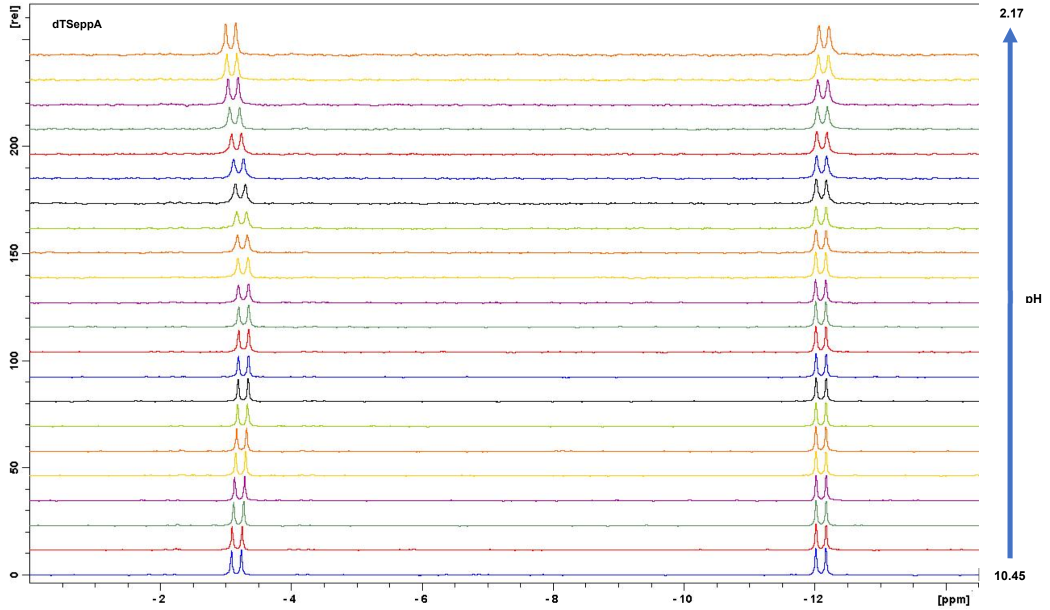

Table S1: Summary of rate law fitting data. The transition state activation values are included for each substrate, along with the R^2 corresponding to application of the Eyring equation with these values to all data for that substrate.

Substrate 5'-dTChMP	T / K	k / 10^{-6}s^{-1}	$[X]_0$ / mM	r^2	T.S. Val
Ch =					
S (**4a**)	298	5.19100	47.7111	0.99949	$\Delta H^‡$ = 126.50 kJ mol^{-1}
	308	17.13283	40.9903	0.99740	$\Delta S^‡$ =78.926 J mol^{-1} K^{-1}
	318	68.40103	41.7548	0.99910	R^2 = 0.98556
Se (**4b**)	298	16.81294	48.6925	0.99947	$\Delta H^‡$ = 110.64 kJ mol^{-1}
	308	75.08776	53.7846	0.99988	$\Delta S^‡$ = 34.988 J mol^{-1} K^{-1}
	318	297.44843	79.1783	0.99889	R^2 = 0.98343

Figure S1: Fits of first order rate equation to reaction data for: **a) 5′-dTSMP ; b) 5′-dTSeMP**

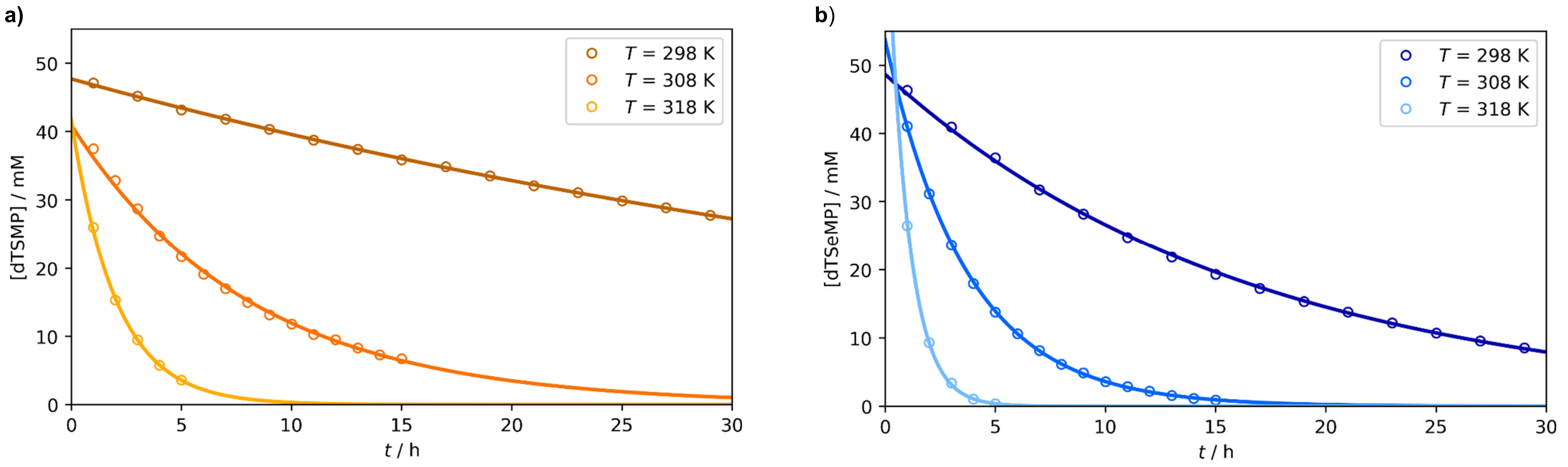

Table S2: Summary of first order rate law fits to pH-dependent reaction data for sulfur and selenium substrates.

Substrate	pH	k / $10^{-6}s^{-1}$	$[X]_0$ / mM	r^2
S	3	151.96065	54.3618	0.99884
Se	3	53.09372	48.7406	0.99949
S	7	5.19100	47.7111	0.99949
Se	7	16.81294	48.6925	0.99947
S	11	0.33763	43.1855	0.99455
Se	11	14.41176	44.4620	0.98994

Figure S2: **Fit of first-order rate laws to reaction data at varying pH values.**

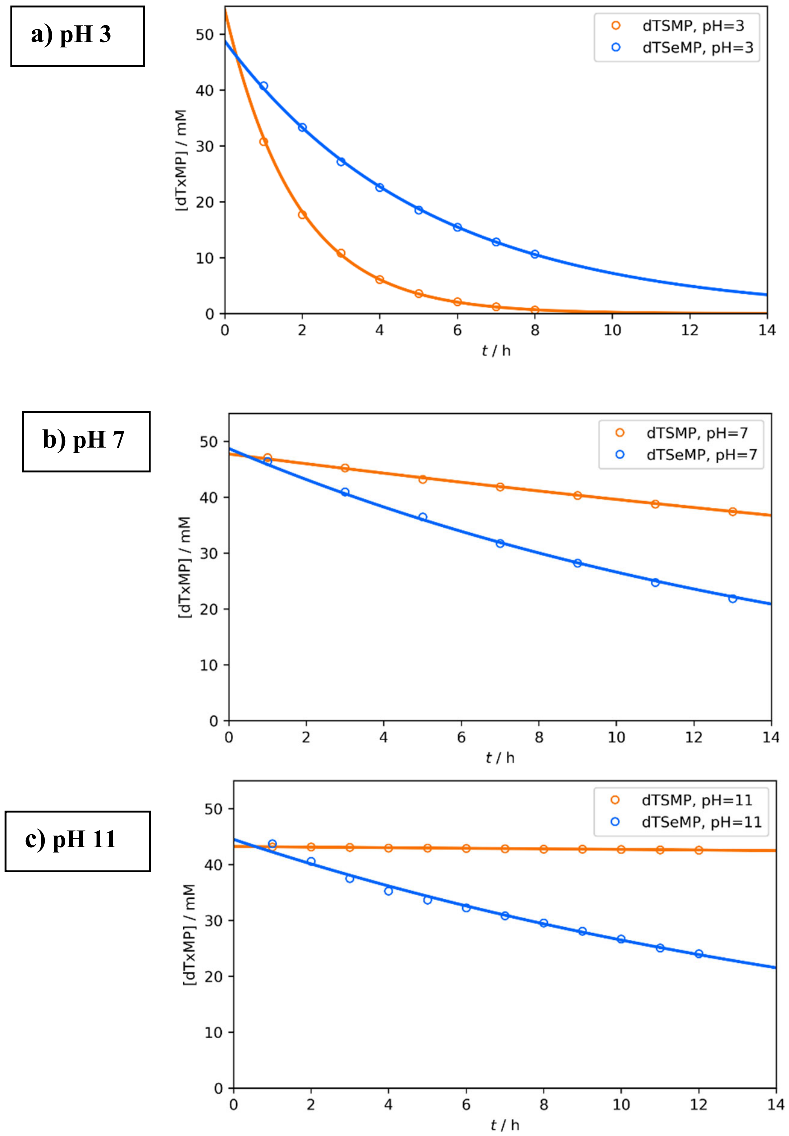

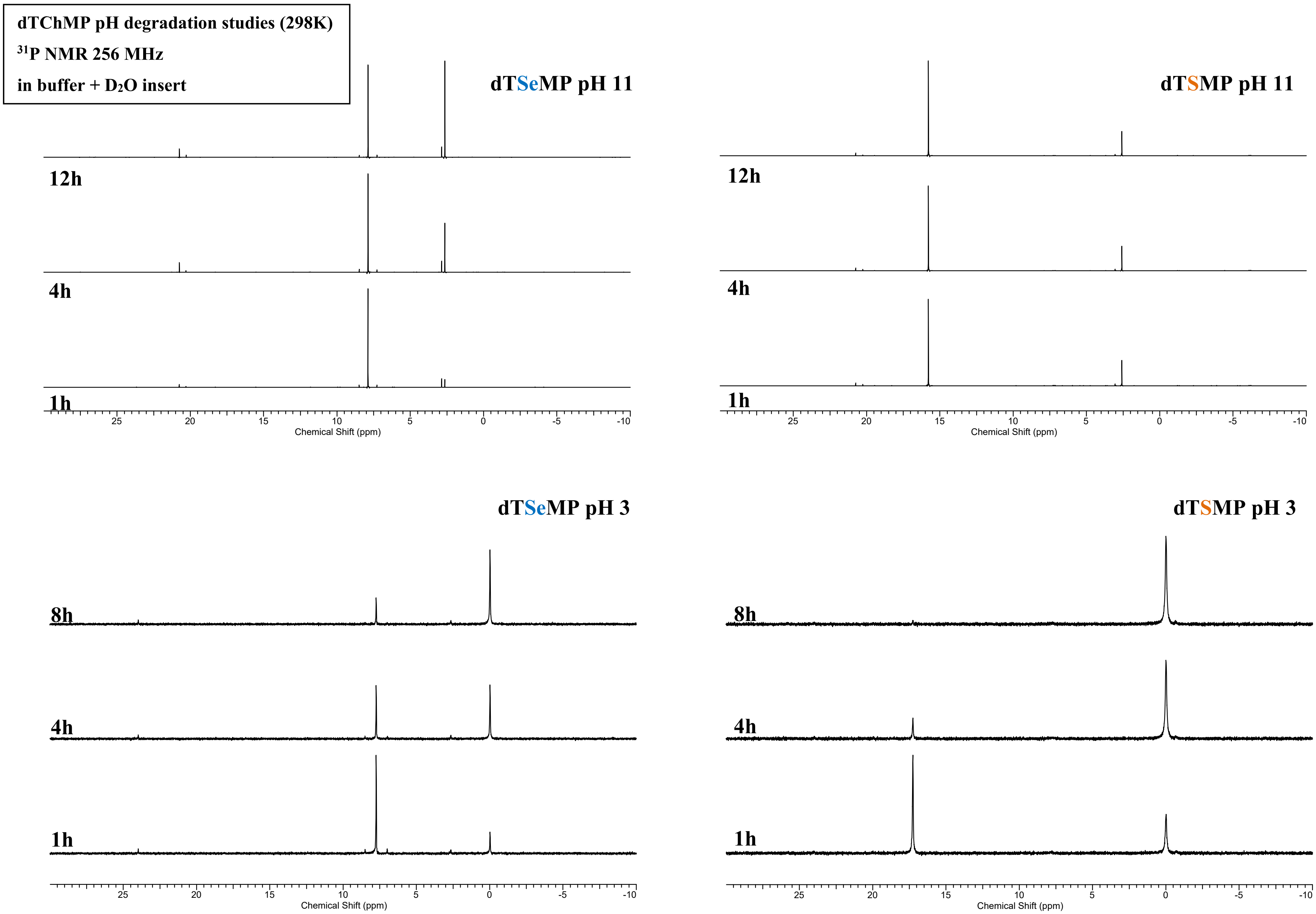
dTChMP pH degradation studies (298K)
31P NMR 256 MHz
in buffer + D2O insert
dTSeMP pH 11
12h
4h
1h
25 20 15 10 5 0 -5 -10
Chemical Shift (ppm)
dTSMP pH 11
12h
4h
1h
25 20 15 10 5 0 -5 -10
Chemical Shift (ppm)
dTSeMP pH 3
8h
4h
1h
25 20 15 10 5 0 -5 -10
Chemical Shift (ppm)
dTSMP pH 3
8h
4h
1h
25 20 15 10 5 0 -5 -10
Chemical Shift (ppm)

Snake venom digestion:

To a solution of 10 mM substrate (NADH/dTSppA (**2a**)/dTSeppA (**2b**)) in aqueous buffer (Tris·HCl (50 mM, pH 7.5), NaCl (50 mM), 1mM $MgCl_2 \cdot (H_2O)_6$) was added a solution of snake venom 0.2 mg/µL (10 µL). The reaction mixtures were incubated at 37°C for 30 minutes. Digestion was monitored by ^{31}P NMR.

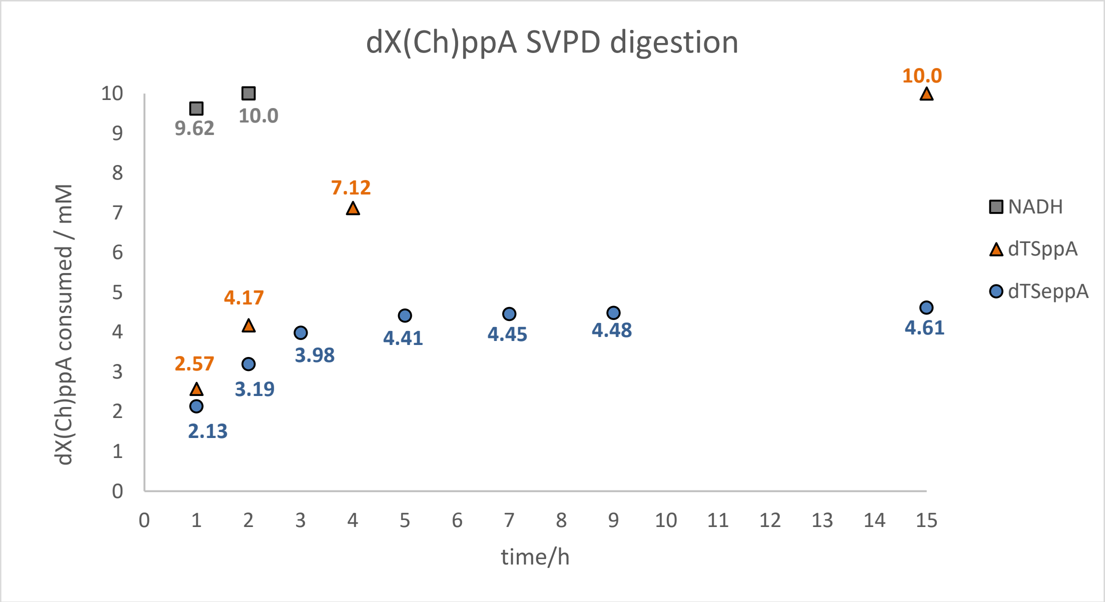

time/h	^{31}P NMR mM NADH consumed	time/h	^{31}P NMR mM dTSppA consumed	time/h	^{31}P NMR mM dTSeppA consumed
1	9.62	1	2.57	1	2.13
2	10	2	4.17	2	3.19
		4	7.12	3	3.98
		15	10	5	4.41
				7	4.45
				9	4.48
				15	4.61
